# A Theoretical Investigation of Novel Sila- and Germa-Spirocyclic Imines and Their Relevance for Electron-Transporting Materials and Drug Discovery

**DOI:** 10.3390/molecules28176298

**Published:** 2023-08-28

**Authors:** Marwan Dakkouri

**Affiliations:** Department of Electrochemistry, University of Ulm, D-89069 Ulm, Germany; marwan.dakkouri@uni-ulm.de

**Keywords:** spirocyclic imines (SCIs), spiro-conjugation, molecular structure, NBO analysis, QTAIM, HOMO-LUMO gap, spiro-aromaticity, drug discovery, charge transfer, functional materials

## Abstract

A new class of spirocyclic imines (SCIs) has been theoretically investigated by applying a variety of quantum chemical methods and basis sets. The uniqueness of these compounds is depicted by various peculiarities, e.g., the incidence of planar six-membered rings each with two imine groups (two π bonds) and the incorporation of the isosteres carbon, silicon, or germanium spiro centers. Additional peculiarities of these novel SCIs are mirrored by their three-dimensionality, the simultaneous occurrence of nucleophilic and electrophilic centers, and the cross-hyperconjugative (spiro-conjugation) interactions, which provoke charge mobility along the spirocyclic scaffold. Substitution of SCIs with strong electron-withdrawing substituents, like the cyano group or fluorine, enhances their docking capability and impacts their reactivity and charge mobility. To gain thorough knowledge about the molecular properties of these SCIs, their structures have been optimized and various quantum chemical concepts and models were applied, e.g., full NBO analysis and the frontier molecular orbitals (FMOs) theory (HOMO-LUMO energy gap) and the chemical reactivity descriptors derived from them. For the assessment of the charge density distribution along the SCI framework, additional complementary quantum chemical methods were used, e.g., molecular electrostatic potential (MESP) and Bader’s QTAIM. Additionally, using the aromaticity index NICS (nuclear independent chemical shift) and other criteria, it could be shown that the investigated cross-hyperconjugated sila and germa SCIs are spiro-aromatics of the Heilbronner Craig-type Möbius aromaticity.

## 1. Introduction

It is widely accepted that the bioisosteric substitution of carbon atoms by silicon in drugs and biologically active molecular systems could lead to a noticeable modification of their initial properties and, in most cases, to an appreciable enhancement of their biological activity and specific functionality. Aside from being less electronegative and possessing a larger atomic radius than carbon, silicon differs from carbon by its hypervalency [1,2] and its capability for forming 5- and 6-coordinated complexes.

Over the past six decades, a vast number of papers and review articles have been published emphasizing the importance of pursuing the development of bio-organosilicon chemistry and the discovery of new vital silicon-based drugs, pharmaceuticals, biocatalysts, functional materials, genetically modified agricultural products [3,4,5,6,7,8,9,10,11], and effective carriers of antibiotics. It is worthwhile to note that relatively little is known about the occurrence of the Si-N chemical bond in pharmacophores and its performance in medicinal chemistry and drug discovery [12]. In contrast, spirocyclic compounds play an important role in the determination of essential properties of drugs and their active agents and, thus, they prove to be crucial for drug discovery. In this context, it can be anticipated that the replacement of a carbo spiro center by a silicon atom with its larger atomic radius and lower electronegativity would lead to an evident reduction in the ring strain in spirocyclic compounds and a charge redistribution within this conformationally restricted three-dimensional scaffold. Based on these features it appeared of interest to incorporate the Si-N bond in a spirocyclic framework creating a new class of silaspirocyclic imines and, thus, enter a new area of novel compounds with multifaceted applications in various fields of applied sciences.

Cyclic imines and spirocyclic imines have attracted the interest of many research groups and have been the focal point of numerous publications [13,14,15,16]. Their occurrence as a pharmacophore in macrocyclic marine biotoxins, e.g., gymnodimine, spirolides, pinnatoxins, etc., imparts this class of compounds’ particular biological activity, like antiviral activity (for example, the portimine inhibition of HIV-1 replication [15] high toxicity, etc.). It is, meanwhile, well-established that these vital properties in these diverse cyclic imino compounds are attributed mainly to the presence of the imino functional group −N=C. Because of the eminent importance of spirocyclic imines for drug discovery and other applications in the fields of agriculture and medicinal chemistry, numerous research groups have focused their activity on developing a new series of this class of compounds.

Spirocyclic imines (SCIs) have been discovered in a group of marine biotoxins characterized by their fast-acting toxicity. Biotoxins are macrocyclic compounds with imine (carbon–nitrogen double bonds) and spiro-linked ethers or polyether moieties. They are grouped together due to the imino group functioning as their common pharmacophore. 

The comprehensive research work on a variety of spirocyclic imines and the key role of the imino moiety in coining the chemical and biological activity of these molecular systems inspired me to investigate a new series of spirocyclic imines (SCIs), consisting of a sequential arrangement of six-membered rings each with two imino groups (two π-bonds) of the type N=X, where X stands for C and Si and Ge for sila-imines and germaimines. It is worth indicating that to the best of my knowledge, nothing is known so far about the occurrence of the imino N=X group in synthetic spirocyclic compounds and its role in coining their chemical and physical characteristics. The distinctiveness of this new generation of SCIs is portrayed by various specific features, e.g., successively perpendicular arrangement of six-membered, cross-hyperconjugated rings (each of which contains two N=C bonds) fused together by a group 14 element, i.e., carbon or the isosteres silicon or germanium as a spiro center. The consecutive “flip-flop” arrangement of the rings within the spiro framework leads to the three-dimensional structure and, thus, to the three-dimensional charge distribution. 

Except for the carbospirocyclic imines, the rings in all other SCIs with Si or Ge spiro centers are planar and all spiro centers within a spirocyclic imine scaffold are colinear. Moreover, the rigidity of the SCIs and the simultaneous occurrence of nucleophilic electron donors (imino group as the Lewis base) and electrophilic charge acceptors (silicon/germanium as the Lewis acid) impart some particular chemical and physical properties. The incidence of imino π-bonds, cross-hyperconjugation (=spiro-conjugation), and the planarity of the six-membered rings in SCIs lead to the assumption that these molecular assemblies should imply aromatic character. This issue will be later closely discussed by invoking the magnetic shielding tensor components represented by the NICS (nuclear independent chemical shift) values. 

All these distinctive characterizations of the sila/germa SCIs testify to their versatility to be utilized in a diversity of advanced and multifunctional materials. Moreover, only little is known about the occurrence of the Si-N chemical bond (and nothing so far about the Si=N bond) in pharmacophores and its performance in medicinal chemistry and drug discovery.

To diversify and enhance some vital chemical and physical properties of the considered SCIs, strong electron-withdrawing and lone pair donating by resonance substituents, like the C≡N group or fluorine, have been introduced to a selection of the SCIs. The structural analysis and the application of various quantum chemical concepts furnished interesting results regarding the impact of these substituents on some crucial properties of the SCIs, e.g., electrophilicity, nucleophilicity, docking capability at molecular assemblies, and functional materials, such as active pharmaceutical ingredients (APIs), lipophilicity, etc.

## 2. Computational Details

For conducting the quantum chemical calculations, Grimme’s double-hybrid functional (DHF) B2PLYP method was predominantly used [17,18]. This computational method has been mainly applied because using higher levels of theory, such as the perturbation theory approach, can be costly and time-consuming. Moreover, as will be shown later, a comparison of the computed geometries of some SCIs as suggested by the double hybrid functional B2PLYP and MP2 methods have produced only marginal differences. 

To account for possible intra-molecular van der Waals dispersion attractive interactions within the sila- and germa-cyclic imines in this work, the double-hybrid functional B2PLYP in combination with the three-body London dispersion correction D3 scheme and Becke–Johnson (BJ) damping [19,20] was used. BJ damping repulsive interatomic forces at shorter distances are avoided, and more reliable and accurate results with respect to intra-molecular dispersion are found, which is of particular importance in the present study. Moreover, the application of the DFT-D3-BJ approach reduces the impact of the basis set superposition error (BSSE) since it is accounted for within the empirical dispersion potential.

An NBO analysis was conducted to obtain some decisive details about the nature of the mutual interaction between the spiro-conjugated cyclic imine skeletons and the reasons for the stability of these spirocyclic imine chains.

The topological properties of the charge density distribution in all spirocyclic imines that are presented in this study have been analyzed by applying the quantum theory of atoms in molecules (QTAIM), which was developed by Bader and others [21,22,23,24]. Bader’s QTAIM provides valuable information about bonding properties in molecules and the reasons for their structural stability. 

Most of the calculations were performed using “tight” optimization criteria and the “ultraFine” grid option. The Gaussian 16 Version B.01 package was used [25], and for processing the graphical representations, GaussView 6 [26] and CorelDraw were applied. 

To avoid frequently occurring convergence problems during the quantum chemical calculations, QC and XQC keywords were applied.

For an appropriate interpretation of the mutual nucleophilic (Lewis base) and electrophilic (Lewis acid) character of the silaspirocyclic imines, the 3D contour maps of the molecular electrostatic potential (MESP) and the HOMO-LUMO frontier molecular orbitals (FMOs) of these compounds have been closely probed. 

Furthermore, for the assessment of the aromatic character in the considered cross-hyperconjugated SCIs, GIAO-NMR calculations were carried out to determine the magnetic isotropic shielding tensor NICS_iso_ (nuclear independent chemical shift) and NICS*zz* the out-of-plane component of the NICS shielding tensor), utilizing the related routine available in Gaussian 16. In a further step, the dependency of the aromaticity descriptors NICS_iso_ and NICS*_zz_* on the distance of the ghost atoms, Bq, from a ring plane and a Si or Ge spiro center (up to 5 Å in increments of 0.5 Å), was investigated.

## 3. Results and Discussion

One of the challenges in this work was finding a proper and concise form for naming the investigated novel SCIs. In the literature, different concepts have been used to designate comparable larger molecular cyclic systems, including spiro frameworks. To avoid any confusion in this regard, only the IUPAC rules for naming similar spirocyclic compounds were utilized. For the sake of brevity, only abbreviations instead of lengthy names are stated. These abbreviations were arbitrarily chosen but are indicative as clearly as possible.

Before proceeding with this paper and discussing the gained results, it is important to explain the scheme that has been applied for the designation of the compounds appearing in this paper:

(1) In all cases, the numbers at the end of the applied acronyms refer to the total number of atoms within the addressed molecule.

(2) In those instances, two or more spirocyclic imines have the same number of atoms but differ only by the arrangement of the imine group with respect to the spiro center. For example, if the spiro center has the form =N-Si-N=, an italic bold ***N*** was added to the acronym chosen to define the SCI. Correspondingly, an italic bold ***C*** was added to the acronym if the spiro center exhibits the form =C-Si-C=. In cases where the spiro center has the form =N-Si-C=, an italic bold ***CN*** was added to the abbreviated name of the SCI. The same scheme was used for SCIs where the spiro atom is carbon or germanium.

**Figure 1:**
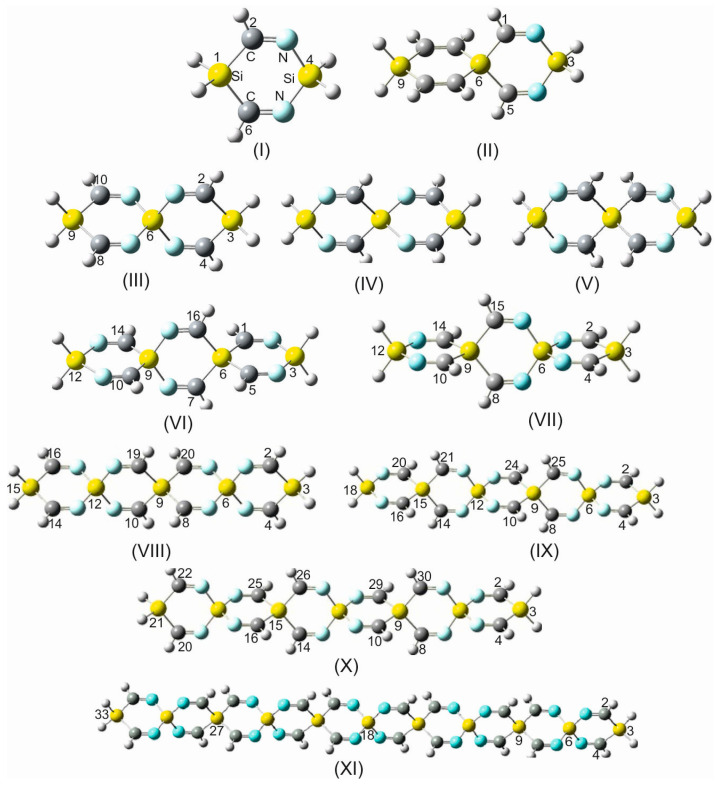
(**I**) 1,4-disilacyclohexa-2-6-diimine (DSCHDI-12). (**II**) 1,5-diimino-3,9-disila-6-silaspiro [5.5]undecane (DISS***C***U-19). (**III**) 2,4,8,10-tertraimino-3,9-disila-6-silaspiro [5.5]undecane (TISS***N***U-19). (**IV**) 2,4,7,11-tetraimino-3,9-disila-6-silaspiro [5.5]undecane (TISS***CN***U-19). (**V**) 1,5,7,11-tetraimino-3,9-disila-6-silaspiro [5.5]undecane (TISS***C***U-19). (**VI**) 1,5,7,10,14,16-hexaimino-3,12-disilsa-6,9-disilaspiro [5.2.59.26]hexadecane (HIDSS***CNC***H-26). (**VII**) 2,4,8,10,14,15-hexaimino-3,12-disila-6,9-disilaspiro [5.2.59.26]hexadecane (HIDSS***NC***H-26). (**VIII**) 2,4,8,10,14,16,19,20-octaimino-3,15-disila-6,9,12-trisilaspiro[5.2.2.512.29.26]henicosane (OITSSHC-33). (**IX**) 2,4,8,10,14,16,20,21,24,25-decaimino-3-18-disila-6,9,12,15,tetrasilaspiro [5.2.2.2.515.212.29.26]hexacosane (DITSSHC-40). (**X**) 2,4,8,10,14, 16,20,22,25,26,29,30-dodecaimino-3,21-disila-6,9,12,15,18-pentasilaspiro [5.2.2.2.2.518.215.212.29.26]hentriacontane (DODIPSSHT-47). (**XI**) 2,4,8,10,14,16,20,22,26,28,32,34,37,38,41,42,45,46,49,50-icosaimino-3,31-disila-6,9,12,15,18,21,24,27,30-nonasilsspiro [2,2,2,2,2,2,2,2,5,26,29,212,215,218,221,224,227,530]henpentaconta (IINSSHPC-75). (**II**) and (**V**) possess the C-Si-C spiro center, (**III**) N-Si-N spiro center, (**IV**) C-Si-N spiro center. (**VI**) C-Si-N and C-Si-C spiro centers. All remaining entities possess alternating N-Si-N and C-Si-C spiro centers.

To facilitate the understanding of the notion that has been utilized for naming the SCIs appearing in this paper and the acronyms that were chosen for characterizing these SCIs, there are some examples and additional examples in Figure 1.

2,4,8,10-tetraimino-3,9-disila-6-silaspiro [5.5]undecane (consisting of 19 atoms) is abbreviated as TISS***N***U-19.

1,5,7,11-tetraimino-3,9-disila-6-silaspiro [5.5]undecane (TISS***C***U-19).

2,4,8,10,14,16,19,20-octaimino-3,15-disila-6,9,12-trisilaspiro [5.2.2.5^12^.2^9^.2^6^]henicosane (consisting of 33 atoms) is abbreviated as (OITSSHC-33). 

(3) In cases where the substituents diiminosilole or diiminogermole are terminally incorporated in a sila or germa SCI, scaffold italic -***V***- is added to the acronym, referring to the vicinal positions of the nitrogen atoms of the imino groups with regard to the SCI framework (e.g., OITSSND-***V***-27 or OITGSND-***V***-27), and -***D***-is added the acronym indicating the distal positions of these nitrogens (e.g., OITSSND-***D***-27 or OITGSND-***D***-27). 

The intrinsic quasi-rigidity and three-dimensionality of the SCIs (including poly-spirocyclic imines) framework and hence the absence of conformational dynamics, in addition to the periodic fluctuation of the charge density distribution between high concentration at the imino fragment N = X (X = C, Si, Ge) and charge depletion at Si/Ge spiro centers, impart these sila and germa SCI-specific chemical and biological activities and qualify them to be particular complementary active agents for drug design, medicinal chemistry, and charge transporting materials. As will be later demonstrated, the addition of specific substituents, e.g., strained heterocyclic entities, like aziridine, azetidine, silole, or germole, introduces noticeable flexibility to the considered sila-/germa-cyclic imines and enhances charge fluctuation along the spiro scaffold. Obviously, depending on the nature of the introduced substituent, both the chemical reactivity and the biological activity are subject to sizeable variations. 

### 3.1. Structural Analysis of SCIs and Some Selected Derivatives

#### 3.1.1. Silaspirocyclic Imines

At this point, a general essential remark should be made. One fundamental strategy of the present study is to analyze the structural and electronic properties of an initial unit of a larger sila- or germa-cyclic imine and then fuse it successively to further subunits, forming the final spiro chain. The major aim of this proceeding is to learn more about the structural and electronic consequences of combining two electron-rich rings (comprising imino groups) joined by a group 14 element and the subsequent fragmentary enlargement of the spiro scaffold. 

Within the frame of this work, quite a wide variety of sila- and germa-spirocyclic imines and their derivatives up to a spiro scaffold consisting of ten six-membered spiro rings, twenty imine groups, and seventy-five atoms have been investigated. A selected variety of silaspirocyclic imines (SSCIs) is shown in Figure 1 (only SSCIs up to 47 atoms because larger scaffolds follow the same pattern). However, because of the complete analogy between the germa-spirocyclic imines (GSCIs) and their silicon counterparts, only some of these GSCIs are shown in Figure S1.

Owing to the periodicity of the structural units within these compounds and, thus, the monotonic recurrence of bond lengths and bonds angles, only the structural parameters of some representatives of these sila- and germa-spirocyclic imines are shown in Table 1, Table 2 and Table 3 and Table S1.

Table 1 reveals that generally, the corresponding bond lengths and bond angles remain almost invariant in all displayed silaspirocyclic imines (SSCIs), indicating that expanding the SSCI scaffold has no structural effect on the units composing its framework.

Only in the asymmetrical HIDSS***NC***H-26 is the Si_6_-N_1_= bond is shorter than the =N_11_-Si_12_ bond by 0.016 Å, and in DITSSHC-40 (Figure 1), the Si_6_-N_1_= and Si_18_-N_17_= bond lengths differ by 0.018 Å. Accountable for this disparity of the Si-N= bond lengths is the fact that the Si_6_-N_1_= is incorporated in a spiro center, while the =N_11_-Si_12_ and the Si_18_-N_17_= bonds are located in a terminal position. From this consideration, it can be concluded that spiro cross-hyperconjugation (spiro-conjugation) is an essential reason for such bond contraction. 

In this regard, it is worth pointing out that in HIDSS***NC***H-26, the natural charges, *q*_NPA_ (NPA stands for natural population analysis, as explained later) on Si_6_ and N_1_, amount to 2.261 e^−^ and −0.896 e^−^ and on Si_12_, N_11_ 1.536 e^−^ and −0.853 e^−^, respectively (as obtained from B2PLYP/aug-cc-pVDZ. Values predicted by B3Lyp//aug-cc-pVDZ were consistently slightly smaller). The reason for the clearly higher positivity of the spiro center Si_6_ in comparison to the second spiro center Si_9_ is that the former spiro center is surrounded by four quite electronegative nitrogen atoms (χ_N_ = 3.41) [27] and the latter is encompassed by four less electronegative carbon atoms (χ_C_ = 2.47) [27]. On the other hand, the electronegativity of the silicon atom is 1.91 [27]. 

As is mentioned in the computational details section above, the main computational method employed was Grimme’s double-hybrid functional B2PLYP. This choice was made because using higher levels of theory, such as the second-order Møller–Plesset perturbation (MP2) method, which considers exchange–correlation effects, is time-consuming. Moreover, the computational results obtained from the MP2 method are typically insignificantly different from those obtained from the B2PLYP method, at least in terms of computing the molecular structures of the investigated SCIs. 

To provide some evidence for the aforementioned statement two examples, TISS***N***U-19 and OITSSHC-33 are shown in Table 2. In this table, it is apparent that generally both the B2PLYP and MP2 computational methods in combination with the Dunning basis set aug-cc-pVDZ suggest very similar values for the geometrical parameters. However, only two exceptions are apparent in this table, i.e., the N_1_=C_2_ and N_7_=C_8_ bond lengths. In these instances, the perturbation method provides larger values, i.e., Δr = 0.013 Å for the former and Δr = 0.014 Å for the latter. The bond angles vary slightly between 1.1° and 1.4°. 

#### 3.1.2. Germa-Spirocyclic Imines (GSCIs)

To examine which role the spiro center plays by the determination of the geometry of the spirocyclic scaffold, the structures of a variety of germa-spirocyclic imines with alternating N-Ge-N and C-Ge-C spiro centers were analyzed.

The aim of this comparison is to examine the effect of including a silicon isostere within the group 14 element, which has a larger atomic radius (r_Si_ = 1.11 Å, r_Ge_ = 1.25 Å), higher electronegativity (χ_Si_ = 1.91, χ_Ge_ = 2.01), and an occupied 3D orbital as a spiro center on the geometrical behavior, including the planarity and reactivity (electrophilicity) of these cyclic imines. It can also be anticipated that the substitution of silicon by germanium in the considered SSCIs can modify some of their essential properties, e.g., the amplification of pharmacophoric characteristics, and has a perceptible effect on the chemical and biological properties of this class of compounds. It is worthwhile to note that drugs containing germanium have proven to possess antiviral and cytotoxic activities and mobilize effects on the immune system [28]. Moreover, in recent years, several papers have been published emphasizing the versatility of germa-spiro compounds as anticancer and antimalarial agents that possess a diversity of pharmacological activities [29,30,31,32]. Before discussing the structural results, which are shown in Table 3, it should be stressed that parallel to the SSCI compounds, all corresponding germa-spirocyclic imines (GSCIs) have also been investigated. 

As can be concluded from Table 3, the structural results of various germa-spirocyclic imines show a comparable trend of invariance upon expanding the size of the spirocyclic scaffold in SSCIs. In harmony with the features that were found in the silaspiro counterparts, alterations of relevant structural parameters occur in the asymmetric HIDGS***NC***H-26 (for reasons of brevity, DITGSHC-40 has not been included in this table). In HIDGS***NC***H-26, the Ge_6_-N_1_= bond as a participant in forming the spiro center is 0.016 Å shorter than the terminally positioned Ge_12_-N_11_= bond. The =N_1_-Ge_6_-N_5_= bond angle, as part of the spiro center, is larger than the terminal bond angle =N_11_-Ge_12_-N_13_= by 2.5°. Moreover, the H-Ge_12_-H angle is larger than the corresponding H-Ge_3_-H angle by 3.8°. 

#### 3.1.3. Structural Spiro Effect 

To investigate the exclusive contribution of the spiro arrangement to the variation of the geometrical parameters of the participating rings forming the spirocyclic scaffold, the following strategy was pursued. The structural parameters of 1,4-disilacyclohexa-3,5-diimine (DSCHDI-12), which represents the primitive subunit of the considered SSCIs in this paper, were compared with the results obtained from the optimized parameters of TISS***N***U-19 (consisting of two DSCDI-12 units fused by a silicon atom as a spiro center). 

**Figure 2:**
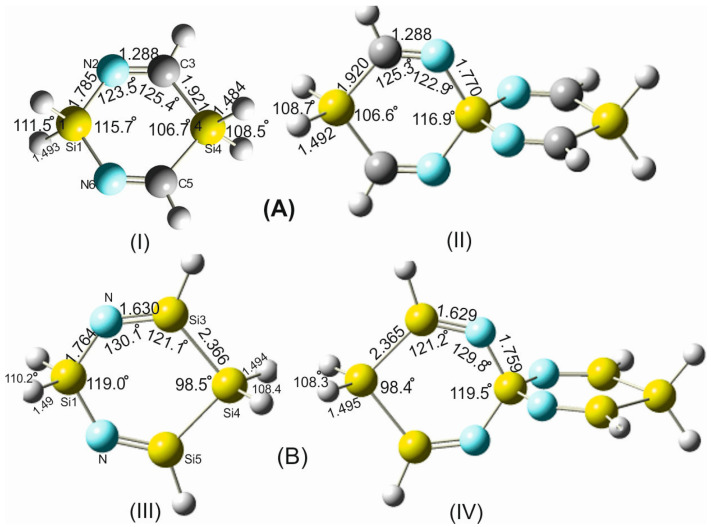
Structural parameters of the building blocks of: (**A**) silaspiro-cyclic imines: (**I**) 1,4-disilacyclohexa-3,5-diimine (DSCHDI-12, (**II**) TISSNU-19 and (**B**) Silaspiro-cyclic silaimines: (**III**) 1,4-disilacyclohexa-3,5-disilaimine (DSCHDSI-12; (**IV**) TSISSNU-19. B2PLYP/aug-cc-pVDZ was used.

From this comparison (Figure 2), it becomes apparent that the bond lengths and bond angles apart from the silaspiro center remain almost invariant. In contrast, the =N-Si bond shortens by 0.015 Å upon the fusion of the two DSCHDI-12 rings forming the spiro subunit TISS***N***U-19 (structural parameters are included in Figure 2). Furthermore, the bond angle incorporating the silicon spiro center in TISS***N***U-19, i.e., the =N-Si-N= bond angle, widens by 1.2°, and the C=N-Si valence angle decreases marginally by 0.6°. 

To gain additional support for these interesting findings, the structures of the analogous compounds 1,4-digermaacyclohexa-3,5-diimine (DGCHDI-12) and TIGS***N***U-19 have been analyzed (Figure S2). Comparison of the structural parameters of these two compounds has affirmed the above-cited alterations of the structural parameters around the spiro center upon the formation of the spiro unit. For example, the =N-Ge bond length in TIGS***N***U-19 shortens by 0.014 Å, the =N-Ge-N= bond angle widens by 1.9°, and the C=N-Ge bond angle declines by 1.1° in comparison to DGCHDI-12. 

All alterations of the structural parameters that have been discussed above are exclusively the consequence of the cross-hyperconjugative interactions within the spiro moiety. This analysis provides direct structural evidence for the formation and stability of an SCI spiro framework, which can be designated as the “structural spiro effect”.

#### 3.1.4. Carbon-Spirocyclic Imines 

**Figure 3:**
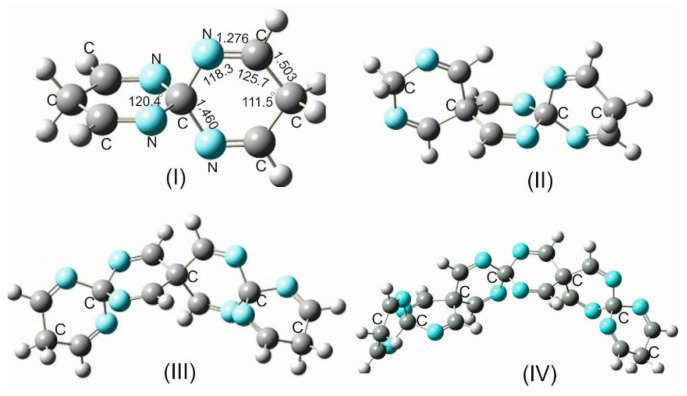
Carbospiro-cyclic imines: (**I**) TICSNU-19. (**II**) HIDCSNCH-26. (**III**) OITCSHC-33. (**IV**) DDIPCSHT-47.

For a systematic investigation of the impact of electronegativity and atomic radius of group 14 isosteres on the structural parameters and rigidity of the spirocyclic imines, the silicon spiro centers were replaced by carbon atoms. Computations at various levels of theory have shown that such a replacement has led in all carbospirocyclic imines (CSCIs) consisting of three rings (and higher number of rings) to a dramatical distortion of the strictly symmetrical linear arrangement of the spirocyclic imine framework and a considerable structural changes and ring deformation (Figure 3). From these results, it can be generally postulated that in all CSCIs (except for the building unit TICS***N***U-19), which are shown in Figure 3, the =C-C and N=C bond lengths, as well as the associated bond angles, are different due to a loss of symmetry within the spirocyclic imine skeleton. It is also worth mentioning that the rings within these CSCIs (except for TICS***N***U-19) are not more planar, and the puckering angle varies between 27° and 30°. 

The main reasons for the non-conformity of the CSIs with SSCs and GSCIs are the substantially smaller covalent radius of the carbon spiro center (r_c_ = 0.75 Å) than the covalent radii of the isosteres Si (1.16 Å) and Ge (1.21 Å), and its higher electronegativity χ_C_ = 2.47 in comparison to χ_Si_ = 1.91 and χ_Ge_ = 2.01 [27]. These factors, in addition to the appreciable repulsive interactions of the approaching electron-rich imino groups, provoke a noticeable increase in ring strain, which the spiro system tries to circumvent by the enforcement of all the geometrical alterations alluded to above. 

#### 3.1.5. Spirocyclic Silaimines and Germaimines (the Si=N and Ge=N bonds)

In a further step aiming to learn more about the relationship between the structural alteration of the investigated SCIs and the associated amendment of their chemical and physical behavior, some representatives of silaspirocyclic silaimines (SSCSIs), involving Si=N functional groups and silicon as spiro centers, have been investigated. Similarly, germa-spirocyclic germaimines (GSCGIs) with Ge=N functional groups and germanium as spiro centers were investigated. Both sila- and germa-spirocyclic imines consist of exclusively silicon/germanium, nitrogen, and hydrogen atoms; see Figure 2 and Figure S3.

It is worthwhile mentioning that silaimines with their strongly polar Si=N bond have been the focal point of several experimental and theoretical investigations, and their relevance for chemical research and other applications has been emphasized [33,34,35]. In contrast, very little is known about the Ge=N bond and germaimines [36,37], and to the best of my knowledge, cyclic germaimines have not yet been investigated. 

As it was alluded to in the introduction, the fundamental relevance of spirocyclic compounds for various fields of applied sciences and functionalized materials has been widely recognized. However, as far as the author knows, no molecular systems, like the novel silaspirocyclic silaimines (SSCSIs) and germa-spirocyclic germaimines (GSCGIs), have been studied so far. Moreover, this unique cross-hyperconjugative spirocyclic class of compounds with their quasi-rigid linear arrangement and alternating electrophilic and nucleophilic (acceptor–donor) centers could be considered as potential building blocks for intra-molecular charge transfer functional conductive and semiconductive materials (perhaps it is best to designate then as “spiro-metallo-organic semiconductors (SMOSC)”) and make them applicable, e.g., for solar cells, optoelectronics, etc., like other related charges transfer spirocyclic molecular systems [38,39,40,41,42]).

Table 4 reveals that the variations of the bond lengths and bond angles upon moving from TSISS***N***U-19 to HSISS***NC***H-26 to OSITSSHC-33 are almost negligible, except that the H-Si_12_-H bond angle in the asymmetric HSISS***NC***H-26 is 1.5° larger than the H-Si_3_-H angle, which can be rationalized by charge withdrawal by the neighboring electronegative nitrogen atoms in the former case.

It is worth mentioning that in life science, the main objective of a bioisosteric replacement (physicochemically or topologically) is to create new molecules with similar biological properties to the parent compounds but with accentuated physiological activity and different pharmacokinetics. For comparison, the structural results provided by B3PLYP/aug-cc-pVDZ (Table S2) for the same spirocyclic-silaimines differ only slightly (the largest deviations are r_N1=Si2_ and r_N7=Si8_, which are smaller by 0.01 Å, and the angles Si_6_-N_1_=Si_2_ and =N_1_-Si_6_-N_5_=, which are larger by 1° and smaller by 1.2°, respectively) from those that are shown in Table 4. 

Similarly, Table S3, in which the structural parameters of the corresponding GSCGIs are listed, shows the same tendency regarding the alteration of the bond lengths and bond angles upon enlargement of the size of the GSCGI skeleton. Likewise, the H-Ge_12_-H bond angle in HGIGS***NC***H-26 is larger (by 2.6°) than the H-Ge_3_-H angle. Certainly, the explanation that has been postulated for the justification of the equivalent bond angle difference in HSISS***NC***H-26 is also here applicable. For comparison, the corresponding geometrical parameters that were obtained from the B3LYP/aug-cc-pVDZ are presented in Table S4.

Perhaps it is worth noting that all symmetrical spirocyclic sila/germa imines and spirocyclic imines (including those with symmetrically substituted derivatives such as fluorine or cyano groups) exhibit no dipole moment. This observation further confirms the rigid nature, complete symmetry, and planarity of the six-membered rings form these spirocyclic molecular systems. Obviously, only the asymmetrical representatives have markedly dipole moments, e.g., HSISS***NC***H-26 has a dipole moment of μ = 1.96 D and TC HSISS***NC***H-30 μ = 2.65 D. 

## 4. Substitution of Spirocyclic Imines 

### 4.1. With Cyano and Isocyano Functional Groups

For the purpose of a systematic investigation of the structural response of SSCIs to the substitution by cyano groups, the terminal hydrogen atoms of the smallest building block of the silaspirocyclic imines 1,4-disila-2,6-diimino-cyclohexane (DSDICH-12) have been replaced by geminal cyano groups, and the computed structural parameters are displayed in Figure S4.

The optimized structural parameters of the tetracyano derivatives of a variety of SSCIs (Figure S5) that are shown in Table 5 unveil the large similarity of these values, regardless of the size of the SSCI scaffold. This tendency parallels the behavior of the structural parameters in the non-substituted parent SSCIs (Table 1). 

The effect of the substitution by the cyano groups emerges from the comparison between the values in Table 1 and Table 5. From this comparison, it is evident that the substitution by the cyano groups has little effect on the bond lengths in these SCIs, except in the case of the asymmetrical HIDSS***NC***H-26 and DITSSHC-40, where the terminal bonds (=N_11_-Si_12_ in the former and =N_17_-Si_18_ in the latter) are shortened by 0.023 Å and 0.024 Å, respectively. In contrast, the bond angles exhibit more response to the cyano substitution, which is demonstrated by the following alterations of the bond angle. The bond angle N_1_=C_2_-Si_3_ declines by 1.7° to 2.0° and the apex angle =C_2_-Si_3_-C_4_= widens by 1.8° to 2.0° on moving from compound (**I)** to (**IV**) in Table 1 and Table 5. The largest increase in bond angles, however, shows the terminal =N_11_-Si_12_-N_13_= and =N_17_-Si_18_-N_19_ in TCHIDSS***NC***H-30 and TCDITSSHC-44 by 2.9° and 3.1°, respectively.

It is noteworthy that in all tetracyano SSCIs included in Table 5, the Si-C≡N chain in the geminal Si_3_-(C≡N)_2_ moiety is bent outwards by 2.6°, whereas this chain in Si_15_-(C≡N)_2_ and Si_18_-(C≡N)_2_ in the asymmetric (**II**) and (**IV**) (Table 5) is bent outwards by 2.1°. Accountable for the non-linearity of the Si-C≡N fragments is the accentuated polarity of the C≡N triple bond and the charge accumulation on the nitrogen atom and, thus, the resulting repulsive interactions between the nitrogen atoms of the geminally substituted cyano groups.

A comparison between the tetracyano and tetraisocyano analogs in Table S5 reveals that, surprisingly, there are no significant alterations of the bond lengths and bond angles, except for the Si_3_-C_2_= bond length. This bond shortens in the isocyanide derivatives TICSSTI***N***U-23 and TICSSOIHI-37 by 0.015 Å, whereas this bond remains unaffected by attachment to the cyano group. This is not particularly surprising because the group electronegativity of the isocyano group amounts to 3.30 and 2.69 for the cyano group [43]. Therefore, the stronger electron withdrawal by the N≡C group leads to a natural charge population of +1.84 e^−^ on Si_3_ (in comparison to +1.38 e^−^ in the case of the C≡N substitution), which is responsible for the contraction of the Si_3_-C_2_= bond.

Parallel to the behavior of the substitution by cyano groups, the geminal isocyano Si-(N≡C)_2_ group in TICTISS***N***U-23 and TICOITSSHC-37 is bent outwards by 4.1°. This nonlinearity of the Si-N≡C groups can be rationalized by the repulsive interactions between the geminal Si-N^+^≡C^−^ groups because of the ionicity of the N^+^≡C^−^ bond and the presence of lone pair electrons on the carbon atoms. 

In consistency with the effect of the geminal substitution of the SSCIs by cyano and isocyano functional groups, the replacement of the terminal hydrogen atoms in various GSCIs by cyano groups shows only a marginal structural influence on the endocyclic bond lengths. A comparison between the structural results of the non-substitute germa-spirocyclic imines GSCIs (Table 3) and their tetracyano derivatives (Table S6) confirms this finding. However, analogous to the bonding behavior in TCHIDSS***NC***H-30, the terminal =N_11,13_-Ge_12_ bonds in the asymmetric TCHIDGS***NC***H-30 present an exception. These bonds shorten by 0.020 Å upon substitution by the cyano groups. This bond contraction can be reasonably explained by applying the same justification that has been described above in the case of the silicon counterpart. Parallel to the pattern that was found in the tetracyano SSCIs, the bonding angles in GSCIs are more sensitive to the influence of the strong charge-withdrawing cyano group. As a result of the withdrawal of electrons from the germa-spiro ring by the geminal cyano moieties, the bond angle =C_2_-Ge_3_-C_4_= involving the anchor atom Ge_3_ widens by 2° in all three displayed tetracyano GSCIs (in comparison to the non-substituted species). As compensation for this angle widening the endocyclic bond angle, N_1_=C_2_-Ge_3_ narrows by 2.0°, 2.2°, and 2.4° in TCGSTI***N***U-23, TCHIDGS***NC***H-30, and TCOITGSHC-37, respectively. Furthermore, the bond angle Ge_6_-N_1_=C_2_ comprising the spiro pivot atom Ge_6_ increases by 1.1°, 1.7°, and 2.0° when moving from the tetracyano GSCI (**I**) to (**II**) and (**II**I) in Table S6. The most apparent impact of the geminal substitution by the cyano groups is reflected by the bond angles Ge_12_-N_11_=C_10_ and =N_11_-Ge_12_-N_13_= in TCHIDGS***NC***H-30, where the former bond angle narrows by 2.4° and the latter widens by 3.5° in comparison to the non-substituted HIDGS***NC***H-26. The main reason for this angle deformation is the loss of charges on Ge_12_ as a result of the simultaneous charge attraction by the adjacent nitrogen atoms and the cyano groups, leading to a noticeably positively charged Ge_12_ (natural charge *q*_NPA_ = 1.79 e^−^) in comparison to Ge_3_ (*q*_NPA_ = 1.37 e^−^). 

It remains to be mentioned that analogous to the behavior of the geminal cyano groups in TC-SSCIs, the Ge-C≡N groups in the Ge (C≡N)_2_ fragment are bent outwards in all TC-GSCIs in Table S6. The nonlinearity of these Ge_3_-C≡N groups amounts uniformly in all these TC-GSCIs to 3.0° (the Ge_12_-C≡N chain in the asymmetric TCHIDGS***NC***H-30 is bent by 2.6°). Obviously, this deviation from linearity is explicable by employing the same scheme of rationalization that has been used above in the case of TC-SSCIs.

At this point, it is worth making the following general remark regarding the substituent effect of the cyano and isocyano groups in the contemplated SCIs. The major impact of the C≡N group as a strong σ- and π-electron acceptor and the N≡C group as σ-donor and π-electron acceptor is predominantly a local effect (bond angles are more affected than bond lengths) and does not propagate through the spiro scaffold of the discussed SSCIs and GSCIs of any size. 

In summary, the substitution of the strained sila- and germa-spiro CI scaffolds by strong electron-attracting moieties, like the cyano or isocyano groups, has a moderate effect on the geometrical parameters (bond angles are more affected than bond distances), which demonstrates once again the rigidity and stability of this class of spirocyclic compounds. The main target of adding such polar substituents is to manipulate the charge distribution along the spiro framework and, therefore, modify the physicochemical properties of such molecular systems, e.g., the enhancement of bond polarity, and accordingly, the accentuation of the electrophilicity and nucleophilicity, as well as the lipophilicity (solubility in fats, lipids, and non-polar solvents) of this class of compounds. Furthermore, such strong polar (and polarizing) substituents fashion the host SSCIs and GSCIs to become most appropriate for docking on other drug active ingredients and charge transferring molecular assemblies and, thus, to qualify them for a diversity of applications in drug research and optoelectronics. These conclusions are also applicable to the impact of the substitution by methyl groups and fluorine atoms, as will be shown later. 

### 4.2. Substitution with Methyl Groups

It is commonly accepted that the methyl group donates electrons via the inductive effect or by means of hyperconjugation. Depending on the electronic properties of the attached moieties, the methyl group can exert a substantial effect on these neighboring fragments.

In addition to the substitution of the SCIs by electron-withdrawing substituents, the hydrogen atoms in some representatives of SSCIs (TISS***N***U-19 and OITSSHC-33) were successively replaced by electron-donating methyl groups (Figure S6), and the effect of such substitution on the geometry of the SSCI scaffold has been scrutinized. In this paper, for the sake of brevity, only the most significant resulting alterations of the geometrical parameters as predicted by the B3LYP and B2PLYP functional methods in combination with the aug-cc-pVDZ basis set will be briefly addressed.

Astonishingly, all bond lengths in the 3,3,9,9-tetramethyl-TISS***N***U-19 remain unaffected (vary only by 0.001 Å) in comparison to the non-substituted TISS***N***U-19. Only the neighboring bond angles C_2_-Si_3_-C_4_ and Si_3_-C_2,4_=N_1,5_ (and, by symmetry, the corresponding angles C_8_-Si_9_-C_10_ and Si_9_-C_8,10_=N_7,11_) (Figure S6) differ by 1° upon substitution (narrowing in the former case and widening in the latter). On the other hand, the attachment of the methyl entities to the four imino groups (2,4,8,10-tetramethyl-TISS***N***U-19) engenders more substantial alteration of the bond lengths and bond angles. As a result, the =C_2_-Si_3_ bond (by symmetry, all =C-Si bonds) prolongates by 0.11 Å upon substitution, and the =N_1_-Si_6_ bond (all four =Si-N bonds) shortens by 0.006 Å. More drastically are the variations of the bond angles. While the bonding angle N_1_=C_2_-Si_3_ (C_2_ is the anchor atom for the methyl group) decreases by 2.3°, the adjacent bond angles C_2_-Si_3_-C_4_ and C_2_=N_1_-Si_6_ widen by 1.8° and 1.6°, respectively. 

Interestingly, the variation of the bond lengths and bond angles on moving from OITSSHC-33 to its tetramethyl derivative 3,3,15.15-tetramehyl-OITSSHC-33 are similar to fluctuations that were obtained by comparing TISS***N***U-19 with 3,39,9-tetramethyl-TSS***N***U-19. This finding indicates that apparently, the influence of the geminal terminal substitution of SSCIs by methyl groups is always comparable, irrespective of the size of the SSCI scaffold, to examine the structural consequences of the substitution of the hydrogen atoms at the central imino groups for electron releasing (via inductive or hyperconjugative interactions) methyl groups.

The structure of 8,10,19,20-tetramethyl-OITSSHC-45 (Figure S6) was computed. Inspection of the collected results has revealed that such a “crowd” of methyl groups has, in consistency with the above-mentioned cases of methyl-SSSCIs, has only a local effect. While all other bond distances in this molecule show almost no fluctuations in comparison to the parent molecule OITSSHC-33, the C_8_-Si_9_ bond distance (C_8_ is the anchor atom for the CH_3_ substituent) expands by 0.012 Å. As has been repeatedly accentuated, the major influence of the substitution of SCIs is manifested by the alteration of the bond angles. Similarly, in 8,10,19,20-tetramethyl-OITSSHC-45, the bond angles Si_6_-N_7_=C_8_ and C_8_-Si_9_-C_20_ increase by 1.0° and 1.6°, respectively, and the N_7_=C_8_-Si_9_ bond angle decreases by 2.1° compared to the non-substituted form. For the purpose of comparability, the molecular structure of 3,3,8,10,15,15,19,20-octamethyl-OITSSHC-57 (where the CH_3_ groups are attached simultaneously in terminal and central positions) was optimized (Figure S6). The predicted structural results have revealed that all alterations of the bond lengths and bond angles are almost identical to those found in 3,3,15.15-tetramehyl-OITSSHC-33 and 8,10,19,20-tetramethyl-OITSSHC-45. This interesting finding represents an additional substantiation for the statement that the substituent effect of the methyl group is a local effect. 

### 4.3. Substitution with Fluorine 

As indicated earlier, the main objectives of introducing particularly strong charge-attracting/donating substituents, like C≡N, N≡C, and CH_3_ functional groups and fluorine, to the electron-rich and quasi-rigid scaffold of the discussed sila/germa SCIs, is to investigate the effect of the rearrangement of the electron density distribution within these molecular systems and the modification of their physicochemical properties. The induced charge redistribution engenders perceptible structural changes and has a noticeable effect on some crucial properties of this class of compounds (in accordance with the structure-function, structure-property relationships) like polarity, intra- and inter-molecular charge transfer characteristics, electronic chemical potential, and global hardness/softness, as will be shown later in this paper.

Owing to its high electronegativity, fluorine exerts substantial field and inductive effects on atoms to which it is attached, i.e., Si-F and C-F bonds. On the other hand, fluorine is capable to donate lone pair electrons to a neighboring antibonding (non-Lewis) 6*orbital. 

In numerous papers, the pivotal role of fluorine substitution is in drug research [44] and charge transport materials. In addition, the concerted multiple fluorinations of frequently used active ingredients contribute to the enhancement of their biological and pharmacological activity, the influence of cell metabolism, and the permeability of cell membranes [45]. Also, the impact of fluorination on charge transfer dynamics and its efficiency across self-assembled monolayers (SAMs) has been investigated [46]. In this work, it was also indicated that the number of incorporated fluorine atoms, as well as their position on the carrier molecule, decisively affect the extent of their induced dipole moment and electron transport dynamics. Additionally, the relevance of fluorination for the enhancement of charge transport mobility of charge transfer complexes has been addressed [47]. It has also been shown that the systematic successive fluorination of silicon-based small molecule semiconductors appreciably influences their push–pull charge transfer capability and, therefore, contributes to the enhancement of their performance and the increase in their versatility [48]. From the above-mentioned, it can be anticipated that the incorporation of fluorine in the sila-/germa-spirocyclic imine framework would modify some of their essential properties, e.g., charge density distribution and charge mobility, the promotion of their already existing lipophilic character as silicon-/germanium-based cyclic imines, and their tendency for inter-molecular interactions and docking at active pharmaceutical ingredients (APIs). Furthermore, most likely the partial or total substitution of fluorine for hydrogen in such spirocyclic imines with their intrinsic three-dimensionality would markedly enhance the lipophilicity of affixed APIs. 

Based on these fundamental perspectives, the hydrogen atoms in some silaspirocyclic imines were successively replaced by fluorine atoms, and the structures of the fluorinated derivatives were analyzed. In this regard, the smallest unit (the building block) of the silaspirocyclic imines 1,4-disila-2,6-diimino-cyclohexane (DSDICH-12) has been gradually fluorinated, and the obtained structural results are shown in Table 6. In this table, it is apparent that the N_2_=C_3_ bond shortens significantly by 0.033 Å upon the substitution of fluorine for the imino hydrogens and by 0.029 Å with the additional replacement of the hydrogens on the silicon atoms.

The strengthening and shortening of the C=N bond will be rationalized later in conjunction with the discussion of the NBO results. Another striking example is the evident shortening of the Si_1_-N_2_= bond by 0.024 Å and 0.035 Å in (**II**) and in (**IV**) in Table 6 upon the substitution of all hydrogens on the silicon atoms. This bond shortening is obviously due to the strong electron-withdrawing effect of the fluorine atoms. 

As mentioned earlier, the introduction of charge withdrawing substituents to SCIs results in more pronounced changes in bond angles compared to bond distances. The most notable alterations are observed in the bond angles N_2_=C_3_-Si_4_ and =C_3_-Si_4_-C_5_=, with the former expanding by 4.6° and the latter contracting by 5.9°. These modifications are attributed to the strong electron withdrawal caused by fluorination on the imine carbon atoms C_3_ and C_5_ (compound **III** in Table 6).

Table 6 also reveals that the variation of bond lengths and bond angles during the successive fluorination of TISS***N***U-19 parallels, albeit to a lesser extent, the changes observed in these structural parameters in fluorinated DSDICH-12. Conspicuous examples of this similarity are the variations of the N_1_=C_2_-Si_3_ and =C_2_-Si_3_-C_4_= bond angles upon the substitution of fluorine atoms for the imine hydrogens ((**VII**) in Table 6). Whereas the N_1_=C_2_-Si_3_ angle increases by 4.6°, the =C_2_-Si_3_-C_4_= bond angle narrows by 5.8° on introducing the fluorine substituents at the indicated positions in Table 6. This behavior testifies that fusing two DSDICH-12 units forming the initial spiro scaffold unit plays a subordinate role by affecting the geometrical parameters in comparison to the dominant role of the strong electronegative fluorine.

It is worth noting that here and elsewhere in this work, the distinct angle deformations upon the insertion of strong electron-withdrawing substituents into SCIs are plausibly explainable to be the consequence of a partial circumvention of Baeyer’s angle strain due to the lack of flexibility of the rigid rings forming the spirocyclic scaffold.

**Figure 4:**
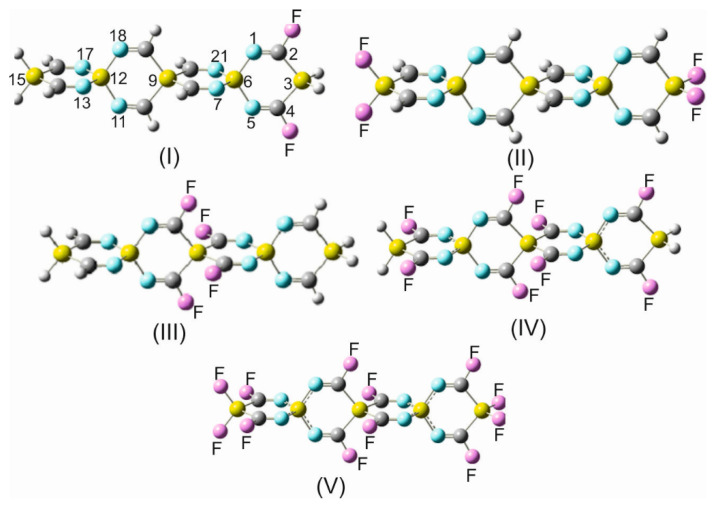
Fluorine derivatives of trisilaspiro-octaimine (OITSSHC-33): (**I**) 2,4-difluoro- (F2-). (**II**) 3,315,15-tetrafluor- (term-F4-). (**III**) 8,10,19,20-tetrafluoro- (cent-F4-). (**IV**) 2,4,8,10,14,16,19,20-ctafluoro- (F8-). (**V**) 2,3,3,4,8,10,14,15,15,16,19,20-dodecafluoro- (F12-).

The substitution of OITSSHC-33 by fluorine at different positions (Table S7) and Figure 4) has led to various noticeable alterations of the geometrical parameters. 

Before discussing the repercussions of the fluorine substitution on these parameters, it is importat to note that (i) OITSSHC-33 has been chosen as a representative for all other SSCIs occurring in this paper. It has been proven that larger silaspirocyclic imines provide negligibly different structural variations on fluorination in comparison to OITSSHC-33 fluorine derivatives. (ii) The main objective for the successive addition of fluorine atoms to the OITSSHC-33 framework is to systematically investigate the impact of the gradual fluorination on the redistribution of the charge density along the spirocyclic scaffold and charge mobility, as well as the accepting/donating efficiency and, thus, the electrophilicity and nucleophilicity of the considered SSCIs. A brief comparison of the structural parameters of the fluorinated OITSSHC-33 in Table S7 with the non-substituted parent entity (Table 1) unveils that the N_1_=C_2_ (N_5_=C_4_) bond shortens by 0.033 Å in the F2- derivative and the Si_3_-C_2,4_= bond lengths decrease by 0.026 Å in the *term*-F4 derivative. In the *cent*-F4 derivative (for the explanation of the prefix *term*- and *cent*-, see the legend for Table S7), the N_7_=C_8_ (N_21_=C_20_) bond shortens by 0.034 Å and the Si_9_-C_8_= bonds by 0.012 Å. Obviously, by increasing the number of fluorine substituents, the sphere of substituent effects increases, and accordingly, additional structural parameters are affected. In the case of the F8 derivative, the N_1_=C_2_, Si_6_-N_1_=, N_7_=C_8_, and Si_9_-C_8_= bond lengths decrease by 0.030 Å, 0.016 Å, 0.032 Å, and 0.11 Å, respectively. Similarly, in the perfluoro OITSSHC-33 derivative (F12- in Table S7), the N_1_=C_2_, Si_3_-C_2_=, Si_6_-N_1_=, N_7_=C_8_, and Si_9_-C_8_= bonds shorten by 0.030 Å, 0.020 Å, 0.012 Å, 0.031 Å, and 0.010 Å, respectively. As was previously recurrently pointed out in this work, the insertion of electron-accepting substituents, like C≡N or fluorine, into the framework of an SCI molecular system leads to a more accentuated effect on the bond angles than the bond lengths. In this context, a comparison between OITSSHC-33 in Table 1 and F12-OITSSHC-33 in Table S7 discloses the following alterations of the bond angles. (i) The bond angles N_1_=C_2_-Si_3_ and N_7_=C_8_-Si_9_ widen perceivably by 2.1° and 3.1°. (ii) In contrast, the angles incorporating a spiro center, e.g., =C_2-_Si_3_-C_4_=, N_1_-Si_6_-N_5_=, =N_7_-Si_6_-N_21_, and =C_8_-Si_9_-C_20_=, narrow by 2.5°, 1.5°, 1.6°, and 4.1°, respectively. 

To examine the dependency of the geometrical parameters of the above-discussed fluorine derivatives of OITSSHC-33 on the applied DFT method, the B3LYP density functional algorithm in combination with the aug-cc-pVDZ basis set was used to calculate these parameters. The collected results, which are displayed in Table S8, show that this conventional DFT method produces similar but mostly slightly smaller values. 

In the same context, the hydrogen atoms in the cyclic sila-imino subunit 1,4-disila-3,5-disilaiminocyclohexane (DSDSICH-12) and the smallest silaspirocyclic sila-imine (SSCSI) unit TSISS***N***U-19 were gradually substituted by fluorine atoms (Figure S7). As is apparent in Table S9, the diverse fluorination of TSISS***N***U-19 as the smallest scaffold unit of the silaspirocyclic silaimine (SSCSI) series reveals the following structural features. (1) The replacement of the hydrogen atoms of the silaimine groups by fluorine atoms (designated as *cent*-F4-TSISS***N***U-19 (**VII**) in Table S9) shortens the N_1_=Si_2_ bond by 0.021 Å and the =Si_2_-Si_3_ bond by 0.014 Å in comparison to the non-substituted parent molecule. This simultaneous bond shortening can be explained by the contraction of the covalent Si_2_ radius, leading to a shortening of both the N=Si_2_ and Si_2_-Si_3_ bonds. (2) The substitution of fluorine atoms for the hydrogens in terminal positions ((**VI**) in Table S9) has a marginal effect on the neighboring bond lengths. One possible elucidation for this striking invariance of the =Si_2_-Si_3_ bond length in comparison to the non-substituted TSISS***N***U-19 is the involvement of the Si_2_ atom in a π-bond and, hence, it is strongly bound to the electronegative nitrogen atom. (3) The per-fluorination of TSISS***N***U-19 ((**VIII**) in Table S9) produces a shortening of the N_1_=Si_2_ bond by 0.023 Å and the =Si_2_-Si_3_ bond by 0.010 Å (almost no alterations of these bond lengths in comparison to compound (**VII**)). (4) In contrast to the bond lengths, the bond angles vary significantly on fluorination (Table S9) to compensate for the slight alterations of the bond lengths and, thus, to account for a partial reduction in the ring strain. For instance, the bond angle N_1_ = Si_2_-Si_3_ narrows by 4.1° and widens by 3.8° upon moving from (**V**) to *term*-F4-TSISS***N***U-19 (VI) and to *cent*-F4-TSISS***N***U-19 (**VII**) in Table S9, respectively. Furthermore, the apex angle Si_2_-Si_3_-Si_4_ increases by 4.4° and decreases by 4.0° in (**V**I) and (**VII**), respectively. (5) Interestingly, the variations of the bond angles in the fluorinated TSISS***N***U-19 derivatives parallel those that were found in the analogous fluorinated DSDSICH-12 derivatives (except for the structural parameters associated with the spiro center Si_6_). 

Scrutiny of the structural results emerging from the fluorination of DSDSICH-12 reveals that the Si_1_-N_2_= bond length shortens by 0.032 Å in (**II**) on the substitution of fluorine for the terminal hydrogen atoms in DSDSICH-12 (Table S9). In accordance with the expectation, the fluctuations of the bond angles, however, are significantly more evident than the bond lengths. The following selected examples corroborate this finding. (i) The N_2_=Si_3_-Si_4_ bond angle in (**II**) (Table S9) is 4.4° smaller and the bond angle Si_3_-Si_4_-Si_5_ is 4.1° larger than in (**I**). (ii) The former angle in (**III**) is 4.1° larger and the latter bond angle is 4.1° smaller than in (**I**). (iii) In (**II**), the bond angle N_2_-Si_1_-N_6_ is 3.1° larger than in (**I**). 

It is worth emphasizing the importance of the three-dimensional spirocyclic compounds as a basic component (building block) in pharmaceutics and drug research or affixed to active pharmaceutical ingredients (APIs). Additionally, the role of fluorine substitution in the enhancement of biological activity, lipophilicity, and accelerating metabolic processes has been thoroughly investigated.

The main rationale for introducing fluorine as a small-sized and highly electronegative atom into compounds is either to enhance their chemical reactivity, to alter their physicochemical properties, or to improve the binding affinity of these compounds.

It should be generally pointed out that adding strong electronegative (or electropositive) substituents to the framework of a spirocyclic imine entity with its duality as nucleophilic (imino nitrogen atoms) and electrophilic (spiro centers) character resembles the application of a steering mechanism, which allows for selectively conducting chemical reactions (electrophilic or nucleophilic attack) under controlled conditions. Moreover, appropriate substituents, e.g., the C≡N group or fluorine, facilitate the charge transfer and specific docking of spirocyclic imines on biologically active agents in drug design and appropriate functional materials.

### 4.4. Substitution with Heterocycles: Aziridine, Azetidine, Silole, and Germole

**Figure 5:**
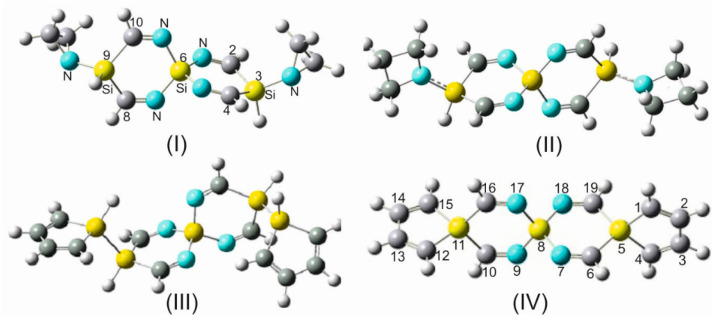
Some silaspiro-cyclic imine derivatives: (**I**) 3,9-diaziridine-2,4,8,10-tetraimino-3-9-disila-6-silaspiro [5.5]udecane (DAZITISSNU-31). (**II**) 3,9-diazetidine-2,4,8,10-tetraimino-3,9-disila-6-silaspiro [5.5]udecane (DAZETISSNU-37). (**III**) 3,9-disilole-2,4,8,10-tetraimino-3,9-disila-6-silaspiro [5.5]undecane (DSTISSNU-37). (**IV**) 6,10,16,19-tetraimino-5,8,11-trisilaspiro [5.2.2.511.28.25]nonadeca-1,3,12,14-tetraene (TITSSNDTE-31).

To increase the functionality and charge transfer mobility of SCIMs and expand the range of applications and versatility of these compounds, the terminal hydrogens in a selection of SSCIs and GSCIs were replaced by particularly vital and chemically active heterocycles, which have been excessively studied and have proven to exert considerable influence on the electronic structure and reactivity of host molecules. The heterocyclic compounds aziridine, azetidine, silole, and germole are part of this category of subunits (Figure 5, Figures S8 and S9).

The addition of such substituents on both ends of an SCI scaffold (single or geminal substitution, as shown in Figure 5, Figures S8 and S9, is anticipated to increase the molecular dynamic of these quasi-rigid molecular assemblies as a consequence of the occurrence of different conformers. Aziridine (ethylene imine) and azetidine (trimethylene imine), as small nitrogen-containing heterocycles, possess multifunctionality, and most of their properties and chemical activities are controlled by the substantial ring strain and the electron crowd on the nitrogen atom. The versatility and the high reactivity of these nitrogen heterocycles have attracted the attention of many researchers working in various fields of chemistry, biochemistry, and physics [49,50,51]. 

The unique electronic structure of the heterocyclic substituents silole and germole and their energetically facilitated charge exchange have drawn special attention. Silole, as well as germole rings, are strong electron acceptors due to their intrinsically low-lying LUMO orbitals, which entitle them to be an essential ingredient in drug research and act as an active component in charge transferring materials (e.g., electron-transporting layers). These decisive properties of silole and germole have been the focal point of interest and the subject of diverse discussions in the literature [52,53,54,55]. The accentuated low LUMO energy is a result of an σ*–π* conjugation interaction between the σ*orbital of the exocyclic σ bonds on silicon/germanium and the adjacent π* orbitals on the doubly bonded carbon atoms. 

This discernible peculiarity of the electronic interactions in silole/germole qualifies them to be an essential building block in electron-transporting materials [53]. In addition to the terminal single and geminal substitution of SCIs by various heterocyclic moieties, silole and germole were incorporated into the spiro scaffold of some of the silaspirocyclic imines. 

**Figure 6:**
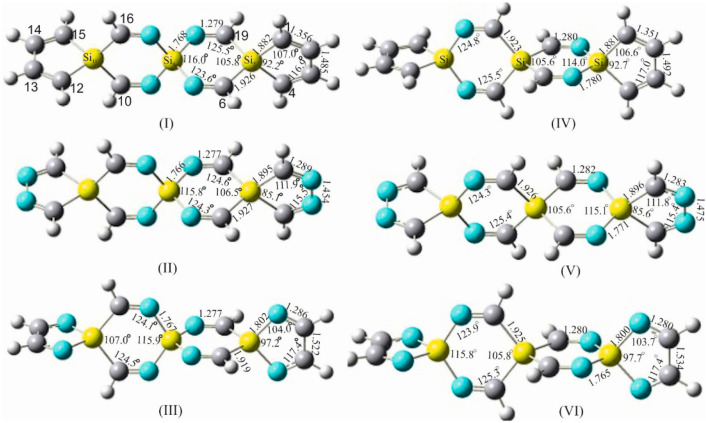
Structural parameters of some sila-cyclic imine derivatives with terminally incorporated silole and diiminosilole in the spirocyclic scaffold. Left: (**I**–**III**) with a N-Si-N spiro center and right: (**IV**–**VI**) with a C-Si-C spiro center. (**I**) 6,10,16,19-tetraimino-5,8,11-trisilaspiro [4.2.2.411.28.25]nonadeca-1,3,12,14-tetraene (TITSSNNDTE-31). (**II**) 1,4,6,10,12,15,16,19-octaimino-5,8,11-trisilaspiro [4.2.2.411.28.25]nonadecane (OITSSNND-***D***-27). (**III**) 2,3,6,10,13,14,16,19-octaimino-5,8,11-trisilaspiro [4.2.2.411.28.25]nonadecane (OITSSNND-***V***-27). (**IV**) 7,9,17,18-tetraimino-5,8,11-trisilaspiro [4.2.2.411.28.25]nonadeca-1,3,12,14-tetraene (TITSSCNDTE-31). (**V**) 1,4,7,9,12,15,17,18-octaimino-5,8,11-trisilaspiro [4.2.2.411.28.25]nonadecane (OITSSCND-***D***-27). (**VI**) 2,3,7,9,13,14,17,18-octaimino-5,8,11-trisilaspiro [4.2.2.411.28.25]nonadecane (OITSSCND-***V***-27). B3LYP-aug-cc-pVDZ was applied.

Moreover, the substitution of the carbon–carbon double bonds in these integrated heterocyclic by C=N- imino groups, as shown by OITSSND-***V***-27 and OITSSND-***D***-27 (and the corresponding germa analogs) in Figure 6 and Figures S10–S12, increases the charge density within the electron-rich silaspiro chains and enhances their π-electron delocalization, and, thus, their electron-transporting efficiency and reactivity. Furthermore, these newly substituted spirocyclic imines represent a novel class of potential building units in drug discovery and medicinal chemistry.

As a representative example for all SCIs, TISS***N***U-19 was chosen and was successively and terminally substituted, either singly (Figure 5) or geminally (Figure S9), with the above-mentioned heterocycles. The structural results, as suggested by the B3LYP/aug-cc-pVDZ method, are listed in Table S10. For reasons of brevity, only 3,9-disubstituted TISS***N***U-19 derivatives are included in this table. Additionally, the structural parameters of TITSS***N***NDTE-31 and TITGS***N***NDTE-31 are shown, where a silole or germole ring is incorporated into the SCI framework (Figure 6). It should be indicated that only noticeable variations of the geometrical parameters are closely discussed. For instance, the bond lengths and angles of the spiro rings are marginally affected by the orientation of the aziridine and azetidine substituents in the 3,9-position, e.g., the difference between the two =C_2_-Si_3_ and =C_4_-Si_3_ bond distances amounts to 0.006 Å, and between the =N_1_-Si_6_ and =N_5_-Si_6_ bonds is 0.004 Å. As is evident in Table S10 that the geometrical parameters of the spiro framework are hardly affected by the terminal substitution with the above-mentioned fragments; rather, the bond distances and bond angles of the substituents themselves vary and, in some cases, even markedly. It is worth indicating that all corresponding substituted germanium analogs have also been investigated but for shortness reasons and because of the strict analogy of the structural variations, they have been left out.

In contrast to the marginal effect on the structural parameters, the substitution by these heterocycles results in a revocation of the strict symmetrical structure of the spiro skeleton. The rings are not perpendicular to each other, and the linear arrangement of the spiro centers is revoked, which demonstrates the considerable impact of this kind of substitution on the internal molecular dynamics and the spatial orientation of the individual component in the spiro framework. This kind of effect of substituents is reflected by the drastic distortion of the planarity of the diamino rings in (**II**)–(**V**) in Table S10, which is visualized by the puckering angle *Θ* ranging from 14.0° to 19.0°. A parallel response to the terminal substitution by the above-cited heterocycles is also manifested in larger representatives of the discussed SCIs. For example, in 3,15-diaziridine-OITSSHC-33 (Figure S8), whereas the bond lengths and bond angles within the silaspiro framework are hardly affected upon substitution, the departure from the linearity of the spiro centers and the distortion of the planarity of the cyclic imine rings are sizable. For example, the Si_3_····Si_6_····Si_9_ angle (equally the Si_9_····Si_12_····Si_15_ angle) deviates by 13° from linearity, and the two external spiro rings to which the aziridine rings are attached pucker by 15° and 22° (depending on the orientation of the aziridine rings in space).

Such geometrical alterations caused by the substitution with heterocycles are corroborated by 3,15-diazetidine-OITSSHC-33. In this case, the deviation from the linear arrangement of the spiro centers amounts to 11°, and the puckering angle of the spiro rings to which the substituents are appended reaches 14°. 

Interestingly, this type of substituent effect is mutual, which means that except for the rigid aziridine ring, all other heterocyclic substituents are influenced by the spirocyclic unit and vice versa. So, for instance, the isolated azetidine ring is puckered by 26.0°, as obtained from B3LYP/aug-cc-pVDZ, whereas this ring is puckered by only 13.0° when bounded to TISS***N***U-19 ((**III**) in Table S10) and is planar in 3,15-diazetidine-OITSSHC-33. Additional examples are compounds (**IV**) and (**V**), in which the silole and germole substituents are puckered by 2.0°, while these heterocycles are planar in the isolated form. 

It is crucial to point out that depending on the orientation of these heterocyclic substituents (silole and germole) in the conformational space, the deviation of the silicon/germanium spiro centers from the linear alignment in the substituted SCIs is differently accentuated. For example, the departure from the linear alignment of the three spiro centers Si_3_····Si_6_····Si_9_ in DAZITISS***N***U-31, DAZETISS***N***U-37 (Figure 5 and Figure S9), in TAZITISS***N***U-43 and TAZETISS***N***U-55 (Figure S9) reaches values of 15°, 16°, 21°, and 28°, respectively. 

To probe the geometrical consequences of the integration of silole into the framework of an SCI entity by fusing it with silicon atoms on both ends of, e.g., TISS***N***U-19, the compounds that are shown in Figure 5 and Figure 6, and Figures S10–S12 have been investigated. From the compared data in Table S10, it is evident that generally, the structural parameters of the central spiro bicyclic tetraimine scaffold in (**IV**) vary only slightly in comparison to those in TISS***N***U-19 (**I**). 

To learn more about the mutual structural and electronic interplay between the incorporated silole/germole fragments and the central spirocyclic imine scaffold, the carbon atoms incorporated in the two C=C bonds in the attached silole/germole rings were successively replaced by nitrogen atoms, forming imine groups in different positions within the five-membered rings (Figure 6 and Figures S10–S12). One of the main reasons for such a replacement was to increase the nucleophilic centers in the spiro system and, thus, to accentuate the nucleophilicity and electrophilicity of the entire resulting spiro framework, including the terminally integrated diamino-silole with the nitrogen atoms in vicinal or distal positions with respect to the spirocyclic imine skeleton (forming N-Si-N or C-Si-C spiro centers), as shown in Figure 6 and Figure S11. Such an introduction of electron-rich diamino-siloles/germoles to an SCI molecular system increases their versatility and expands their capability for docking on other chemically or biologically active agents and their ability to act as mediators for electron transfer processes. In a similar manner, germole and diamino germole moieties (also having vicinal/distal positions of the imino nitrogen) were incorporated in a TIGS***C***U-19 scaffold, and the predicted structural parameters by using the B3YP/aug-cc-pVDZ computational method are shown in Figure S10. 

In summary, in this chapter, it could be shown that the inclusion of various electron-donating r-accepting substituents, i.e., C≡N, N≡C, CH_3_, F, and heterocyclic 3–5-membered rings with nitrogen, silicon, or germanium as hetero atoms into an SCI scaffold plays a decisive role by changing the charge distribution and, therefore, affecting the chemical and physical properties of the host SCI. 

It is worth emphasizing that the three-dimensionality of the building blocks in pharmaceutics and drug design has proven to offer fundamental advantages in the effectivity and targeted functionality, which are significantly more than planar carbon-based analogs. Accordingly, in recent years, pharmacologists and medicinal chemists have emphasized investigating three-dimensional biologically active agents with spiro skeletons. Such entities have been demonstrated to possess considerably higher biological activity and fewer side effects due to their three-dimensional spatial arrangement and charge mobility.

In this context, SCIs with their three-dimensionality, the simultaneous occurrence of internal nucleophilic electron donors (imine groups) and electrophilic charge acceptors (the silicon/germanium spiro centers) and the cross-hyperconjugative interactions that provoke electrons to shuttle back and forth (enhancement of charge mobility) along the spirocyclic framework, demonstrate the wide range of their potential application fields. 

Finally, all these distinctive characterizations of the novel family of sila-/germa-spirocyclic imines certify their versatility to be utilized in a diversity of pharmaceutical, bioactive, and advanced multifunctional materials.

## 5. Dispersion Corrections (Grimme’s D3-BJ Damping)

London dispersion interactions play a prominent role by stabilizing molecular assemblies via inter- and/or intra-molecular attractive interactions. To account for correlation effects and London dispersion energy corrections, Grimme’s atom pair-wise D3 approach [56,57,58], in which the individual contributions from each atom pair are summed up to provide the total dispersion energy, was employed. For a more accurate consideration of the attractive and repulsive interactions and comparison reasons, the D3 Becke–Johnson (D3-BJ) dispersion correction scheme [19] was used. The BJ damping (finite damping instead of zero-damping) is based on the derivation of dispersion coefficients from an exchange dipole moment, which comprises less empiricism than D3 dispersion correction. BJ damping has also the advantage of avoiding repulsive interatomic interactions at shorter distances (for instance, Pauli repulsion) and furnishes better results for non-covalent bonds and intra-molecular dispersion interactions. Moreover, the DHF-D3-BJ method accounts for a substantial part of the basis set superposition error (BSSE) within the empirical potential. 

Using the DHF-D3-BJ method, the geometries and the dispersion corrections of some of the SCIs have been computed, and the results are summarized in Table S11. In this table, it is apparent that with the growing molecular size of the spiro scaffold the stabilizing dispersion energy E_D3-BJ_ increases significantly. For instance, when moving from TISS***N***U-19 to OITSSHC-33, the E_D3-BJ_ value rises by 14.8 kcal·mol^−1^. This is not particularly surprising because these corrections represent the sum of the individual contributions from each atom pair to the total dispersion energy. Interestingly, the E_D3-BJ_ value shows the same tendency within the tetracyano derivatives. Also, in this case, the increase in the E_D3-BJ_ value amounts to 14.8 kcal·mol^−1^ when going from TCTISS***N***U-23 to TCOITSSHC-37. Additionally, in Table S11, the terminal substitution of the sila- and germa-spirocyclic imines with geminal cyano groups leads to an increase in the E_D3-BJ_ value uniformly by approximately 6.0 kcal·mol^−1^, regardless of the size of the spiro framework. Moreover, it is noteworthy that regardless of the degree of fluorination of OITSSHC-33, the dispersion correction increases only marginally, and the increment upon the successive fluorination is steady but relatively small. From these details, the following short conclusions can be derived. (i) The D3-BJ dispersion correction is substantial when terminal hydrogen atoms in the smallest spirocyclic imines are replaced by cyano groups. (ii) The successive substitution of fluorine for the hydrogen atoms in OITSSHC-33 results in small variations of the dispersion damping. (iii) Grimme’s D3-BJ dispersion corrections for OITSSHC-33, its fluoro-derivatives, and all tetracyano-derivatives in Table S11 are overestimated and, to some extent, are even unrealistic (21–36 kcal·mol^−1^). It is conceivable that the prevailing dispersion interactions in this new series of spiro compounds cannot be adequately described by such a concept (D3-BJ) of empirical dispersion corrections. Perhaps the more advanced Grimme’s D4 dispersion correction scheme [59] or other dispersion correction approaches could provide more reasonable and pertinent dispersion corrections for this class of spiro molecules. (iv) Nevertheless, it should be noted that additional sila-/germa-spirocyclic imines of different sizes and constitutions should be investigated in order to gain deeper insight into the role and systematic behavior of the attractive stabilizing dispersion corrections in these novel SCIs. 

## 6. NBO Analysis 

### 6.1. NPA versus MPA Charge Population Analyses

An additional tool that is, in many respects, helpful in the analysis and rationalization of structural results and substituent effects is the charge distribution analysis.

In the literature, there is a variety of approaches aiming to derive more reasonable definitions and reliable values for atomic charges [60,61].

Among these methods are MPA (Mulliken population analysis), NPA (natural population analysis), AIM (atoms in molecules), atomic polar tensor (APT) [62], and the Merz–Kollman (MK) scheme [63], in which semiempirical methods were applied to obtain atomic point charges from the electrostatic potential (ESP). In this work, the focus will be exclusively on the two most popular approaches: the natural population analysis (NPA) [64] and the Mulliken population analysis [65]. Table 7 shows the NPA charges in some selected SCIs and their tetracyano derivatives. Particularly interesting is the behavior of the distribution of charges between atoms participating in bonds involving silicon or germanium spiro centers (Si_6,12_ in (**V**) and (**VI**) and Si_6/_Ge_6_ in all remaining SCIs. As is apparent from this table, the largest positive charges are located on Si_6,12_ (*q*_Si6,12_ = 2.26 e^−^), where the silicon atoms are surrounded by four nitrogen atoms. This is obviously because the electronegativity of nitrogen is considerably higher (χ_N_ = 3.41) [27] than that silicon (χ_Si_ = 1.91 [27]. In (**III**) and (**IV**) where the spiro center Si_6_ and similarly Si_9_ in (***V***) and (***VI***) are surrounded by four carbon atoms (C-Si-C centers), the positive charges on those silicon spiro centers drop down to 1.46 e^−^, which can be anticipated because the electronegativity of the carbon atom (χ_C_ = 2.471) [27] is clearly smaller than nitrogen. For the same reasons and based on the indicated difference between the electronegativities of N and C on one hand, and Si on the other hand, the positive charges on the terminal silicon atoms (Si_3,9_ in (**I**) and Si_3,15_ in (**V**) amount to 1.10 e^−^, whereas in (**III**), where the terminal silicon atoms are adjacent to nitrogen atoms, the positive charge on Si_3,9_ is 0.44 e^−^ larger than in (**I**) and (**V**). A comparison of the NPA atomic charges in TISS***N***U-19 and its germanium counterpart TIGS***N***U-19 shows that Ge_3,9_ and Ge_6_ are slightly less positively charged (by 0.13 e^−^ and 0.14 e^−^, respectively) than the corresponding Si_3,9_ and Si_6_, which is explicable by the slightly higher electronegativity of the germanium atom (χ_Ge_ = 2.01) [27].

It is also evident in Table 7 that the terminal geminal substitution of the addressed SCIs with a strong electron-accepting group, like the C≡N group, leads to an anticipated depletion of charges on the anchor silicon atoms and, thus, an increase in the positivity of these atoms. Nonetheless, the influence of the C≡N substituent on the charge distribution is predominantly a local effect and affects only marginally the charge population on the remaining atoms in the spirocyclic framework.

To scrutinize the performance of the Mulliken charge and its comparability with the natural charges, the Mulliken atomic charges for TISS***N***U-19, OITSSHC-33, and their tetracyano derivatives were calculated, and the predicted results are summarized in Table 8. 

In this table, it is apparent that the atomic charges deviate considerably from the corresponding NPA charges in Table 7. The most striking inconsistency is demonstrated by the negative charges on Si_3,9_ and H(-C) and the positive charges on C_2,4_. This charge inversion reveals that the Mulliken scheme clearly fails to produce the correct charges on most of the participating atoms within the selected SCIs.

This deficiency and all other flaws of the Mulliken concept have been repeatedly discussed in numerous papers expressing fundamental criticism regarding the occasionally quite poor performance of this charge population concept [64]. The main criticism is directed toward the accentuated dependency of the Mulliken population analysis on the quality of the applied basis set, particularly its augmentation with diffuse functions. Moreover, in many examples, it was shown that the Mulliken assignment of negative and positive charges to atoms is sometimes interchanged and contradicts the requirement of their electronegativities. The major reasons for these drawbacks originate from its initial formalism, which arbitrarily suggests an equal partitioning of electron density between participating atoms, and the assumption that diffuse functions assigned to one atom in a chemical bond reside only on this atom. 

To gain more insight into the response of the charge distribution to the attachment of substituents at various positions of an SCI skeleton, the NPA, as well as the Mulliken charge populations within the fluorinated derivatives of OITSSHC-33, were calculated, and the obtained results are shown in Tables S12 and S13. 

A brief comparison between the NPA charge distributions within OITSSHC-33 in Table 7 and its fluorinated derivatives in Table S12 unveils that (1) the charges on the nitrogen atoms in the imino groups are only slightly affected by the addition of fluorine substituents on the neighboring carbon atoms. (2) In accordance with the expectation, the electron depletion on the anchor atoms where the fluorine atoms are attached to is considerable. (3) Strikingly, the positively charged silicon spiro centers, Si_6,12_ and Si_9_, remain almost unaffected by the addition of fluorine substituents at various positions in the spiro scaffold, in contrast to the corresponding Mulliken atomic charges that are listed in Table S13. This table shows fluctuating and, in some cases, irrational values (among them, negative charges on silicon atoms). 

To demonstrate the notorious dependency of the Mulliken charge distribution and the large independency of the NPA charges on the quality of the applied computational method and basis set, both population analyses were conducted on a variety of substituted and non-substituted SCIs by employing the B3LYP and B2PLYP functions and Dunning’s aug-cc-pVDZ basis set (in some cases the non-augmented version of this basis set was also employed). A comparison of the collected results, which are shown in Tables S14–S16, with those that were obtained from the B2PLYP/aug-cc-pVDZ computational method (Table 7 and Table 8) confirms the apparent dependency of the Mulliken atomic charges on the level of the utilized method and basis set. For instance, the double-hybrid functional scheme in combination with the basis set aug-cc-pVDZ predicts the following Mulliken atomic charges: 0.284, −0.204, 2.493, 0.356, and 0.915 electrons on the atoms C_2_, Si_3_, Si_6_, C_8_, and Si_9_ (Table 8) in OITSSHC-33 using the B3LYP/cc-pVDZ method. However, it also provides the values −0.024, 0.322, 0.614, −0.044, and 0.449 electrons, whereas the B3LYP/aug-cc-pVDZ level of theory suggests 0.390, −0.700, 1.898, 0.421, and 0.771 electrons (Table S14) for the same order of atoms. From these apparent variations (signs and magnitudes) of the Mulliken charges, it is evident how strongly this charge population procedure is dependent on the chosen level of theory. 

Another example showing this inherent methodical deficiency of the Mulliken population analysis (at variance with the NPA charge population scheme) is the investigation of the response of both addressed charge population analyses to the choice of the employed computational routine, which is illustrated by the fluoro-derivatives of OITSSHC-33 (Fx-OITSSHC-33). The predicted results by the B3LYP/aug-cc-pVDZ method are presented in Tables S15 and S16 with brief comments and a comparison of these data with those that were produced by the B2PLYP/aug-cc-pVDZ method (Tables S12 and S13). 

To obtain additional knowledge about the behavior of the charge population in a spiro system, an NPA analysis was carried out on the cyclic sila-imines TSISS***N***U-19 and OSITSSHC-33 (in analogy to TISS***N***U-19 and OITSSHC-33) by applying the B3PLYP and B2PLYP functionals in combination with the basis set aug-cc-pVDZ. A cursory comparison between the natural charges in Table S17 discloses that the positive charges on the terminal silicon atoms Si_3,15_ in OSITSSHC-33 are almost negligible (0.04 e^−^), and the central silicon Si_9_ is negatively charged (−0.667 e^−^). Perhaps the unique position of this silicon atom as a spiro center surrounded by four silicon atoms that are attached to nitrogen atoms is responsible for this peculiarity. Attracting electrons from silicon by nitrogen in the N=Si bond results in a lower effective electronegativity of this silicon atom in comparison to the electronegativity of the silicon spiro center. This striking finding prompted me to perform an NPA charge distribution analysis on two silaspiro compounds, in which the silicon spiro center is surrounded by four silicon atoms, i.e., 1,5,7,11-tetrasilaimino-3,9-disila-6-silaspiro [5.5]undecane (TSIDSS***Si***U-19) and 1,4,7,10-tetradisilene-3,9-disila-6-silaspiro [5.5]undecane (TDSEDSS***Si***U-23), which consist of two unsaturated six-membered rings (two Si = Si double bonds in each) fused by a silicon spiro center that exclusively contain silicon atoms. The applied B2PLYP/cc-pVDZ and B3LYP/aug-cc-pVDZ provided NPA charges of −0.216 e^−^ and −0.236 e^−^, respectively, on the silicon spiro center Si_6_. In addition, the NPA charges on the spiro center Si_6_ in the tetrasilaimino analog (TSIDSS***Si***U-19) amount to −0.661 e^−^ and −0.665 e^−^, as predicted by B2PLYP/cc-pVDZ and B3LYP/aug-cc-pVDZ, respectively. Another example in this regard is 1,5,7,11-tetrasilaethylene-3,9-disila-6-silaspiro [5.5]undecane (TSESS***Si***U-23), where the spiro silicon atom Si_6_ is negatively charged with −0.445 electrons. Furthermore, an NPA analysis was carried out on the acyclic tetradisilene-silane (Si (-SiH=SiH_2_)_4_) and tetradisilane-silane (Si (SiH_2_-SiH_3_)_4_), and it turned out that in these cases, negative charges are concentrated on the central Si atom (−0.229 e^−^ in the former compound and −0.238 e^−^ in the latter). Interestingly, the NPA charge distribution in the germanium counterparts to all the above-cited compounds has exhibited the same pattern, i.e., the central germanium atom is always negatively charged when it is surrounded by Ge atoms. This remarkable behavior of the NPA charge distribution (the silicon/germanium spiro center is positively charged when the surrounding bonded atoms are doubly bonded nitrogen or carbon but negatively charged when the Si/Ge spiro center is surrounded by Si(Ge)=X double bonds (X=C, Si, Ge)) or Si-Si (Ge-Ge) single bonds will be the subject of a future investigation.

### 6.2. Bond–Antibond (Lewis Non-Lewis) Interactions 

For the evaluation of the criteria for the stabilizing bond–antibond NBO interactions, the NBO computational routine [66], which is incorporated in the Gaussian 16 program, has been utilized. Table 9 summarizes the values of the donor–acceptor delocalization energy, which are E(2) for sila-/germa-spirocyclic imines TISS***N***U-19 and TIGS***N***U-19 and their tetracyano derivatives, and Table 10 presents some relevant E(2) *values* for OITSSHC-33 and its fluorine derivatives, as predicted by the second order perturbation theory (SOPT) in the NBO basis [67]. 

These energy values depict the extent of charge delocalization from Lewis- to non-Lewis (bond–antibond) NBOs and, thus, indicate the strength of bond–antibond conjugative or hyperconjugative interactions between NBOs.

Scrutiny of the values comparing the second-order perturbation energies (SOPE) of some important intra-molecular donor–acceptor charge delocalization in Table 9 and Table 10 leads to the following intrinsic conclusions (only values larger than 2.0 kcal∙mol^−1^ are listed in these tables, and donor/acceptor interactions involving C-H bonds have not been considered): 

(i) Perhaps the most decisive Lewis to non-Lewis charge transfer is the π_(2)N1=C2_ → σ*_Si6-N7=_ donor–acceptor interaction in TISS***N***U-19 and TC-TISS***N***U-23 (and similarly in the germa counterparts) with a stabilization energy of 3.3 kcal mol^−1^ and 3.2 kcal mol^−1^, respectively. Such kind of NBO interaction can be considered clear evidence for the cross-hyperconjugative interaction in this class of sila spirocyclic imines. This essential assessment will be affirmed later in this work by discussing the spiro-aromaticity of SCIs. For the sake of brevity and because the E(2) values for the analog germanium compounds in Table 9 parallel those that have been presented above, a consideration of the E(2) values for the latter compounds will be renounced. It should only be noted that except for some values, the SOPT analysis provides mostly larger delocalization energies for the germanium compounds than the silicon counterparts. 

(ii) The considerably high stabilization energy for the delocalization from the donor orbitals of the lone pairs on the imino nitrogen N_1_ into the acceptor NBOs σ*_Si3-C2=_ and σ*_Si6-N5=_ undoubtedly play a prominent role by stabilizing the rigidity of the spiro skeleton. 

(iii) The NBO analysis furnishes pertinent evidence for charge transfer and redistribution of the entire electronic structure of a specific molecular entity. In this respect, structural and electron density alterations provoked by the introduction of substituents are clearly detectable by applying the perturbation theory energy analysis on the NBO basis. The validity of such valuation is illustrated by the NBO analysis of the cyano- and fluorine-derivatives of some SCIs (Table 9 and Table 10). The addition of such substituents to SCIs engenders perceptible structural changes and has a noticeable effect on some crucial properties of this class of compounds like increased polarity, intra-, and inter-molecular charge transfer dynamics, electronic chemical potential, and global hardness/softness, as will be shown below. The SOPT analysis of TCTISS***N***U-23 (Table 9) has shown that the interaction between the nitrogen lone pair in the cyano group and the localized Si-C≡ antibond, (LP)_N≡17_ → σ*_Si3-C≡16_), possesses an appreciable delocalization energy of 7.9 kcal mol^−1^. From this analysis, it also emerges that charge transfer occurs from the Lewis-type donor NBO σ_C16≡N17_ to the non-Lewis acceptor NBO σ*_Si3-C≡16_ (E(2) = 2.6 kcal mol^−1^), and a donation of electrons from the occupied π_(2)C16≡N17_ bonding NBO to the antibonding acceptor σ*_Si3-C≡18_ orbital results in a stabilization energy of 2.4 kcal mol^−1^. These two orbital interactions manifest the hyperconjugative interaction within the N≡C-Si-C≡N fragment (electrons are commuting between the terminal nitrogen atoms), which rationalizes the abovementioned shortening of the Si_3_-C≡ bond and, at the same time, they confirm the specific bonding character of the cyano group as a simultaneous strong 6 and π acceptor. To add more evidence to the justification of this kind of internal orbital interaction between the geminal cyano groups, a combined structural and NBO analysis on 3,9-dicyano-TISS***N***U-21 and 3,9-dicyano-TIGS***N***U-21 (only one cyano group on each side) has been performed. Interestingly, the elimination of the geminal arrangement of the cyano groups has led to the following consequences. (1) The Si_3_-C≡ bond lengthens by 0.01 Å in comparison to the Si_3_-C≡ bond in TCTISS***N***U-23. (2) The C≡N bond length remains unaffected (1.174 Å). (3) The endo cyclic bond angle =C_2_-Si_3_-C_4_= decreases by 1.6°. Similarly, the Ge_3_-C≡ bond length in 3,9-dicyano-TIGS***N***U-21 increases by 0.01 Å in comparison to the corresponding bond in TCTIGS***N***U-23, and the endo cyclic bond angle =C_2_-Ge_3_-C_4_= narrows by 3.2° due to the absence of the electronic coupling, which was prevailing in the case of the geminal substitution. 

(iv) In Table 9, it is also apparent that the most energetically favorable Lewis to non-Lewis charge delocalization occurs by the donation of the electron lone pairs on the nitrogen atoms in the imino and cyano groups. In this respect, Table 9 indicates that the largest charge delocalization energy of 12.2 kcal mol^−1^ in TISS***N***U-19 andTCTISS***N***U-23 (and correspondingly, 13.1 and 13.4 kcal mol^−1^ in TIGS***N***U-19 and TCTIGS***N***U-23) is associated with the (LP)_N1_ → σ*_Si3-C2=_ (and (LP)_N1_ → σ*_Ge-C2=_) donor–acceptor orbital interaction, leading to a strengthening of the Si_3_-C_2_= (and Ge_3_-C_2_=) bond. The markedly large value of the stabilization energy of 8.6 kcal mol^−1^ for the (LP)_N≡17_ → σ*_Si3-C≡16_ (and obviously for the (LP)_N≡19_ → σ*_Si3-C≡18_) charge delocalization contributes to an additional strengthening of the Si_3_-C≡ bond and simultaneously to a slight weakening of the C≡N bond. 

Among the essential information provided by the NBO analysis is the occupancy of bond orbitals and their departure from the idealized Lewis structure. Table S18 reveals that all 6- and π-bonding orbitals in the C=N and C≡N bonds are obviously polarized toward the higher electronegative nitrogen atom. For instance, the 6_N1-C2_ NBO consists of 60.7% electrons localized on a nitrogen natural atomic hybrid and 39.3% on a carbon natural hybrid (this partitioning of the NBO is in TISS***N***U-19 and TCTISS***N***U-23, which are almost alike). The accentuated polarity of the =N-Si bond is demonstrated by the large polarization coefficient ξ(N_1_) of 0.898 (80.6% of the NBO) on the nitrogen atom and ξ(Si) = 0.44 (19.4% of the NBO) on silicon. As is also shown in Table S18, the Si-C≡ bond in TCTISS***N***U-23 is less polar than the =N-Si bond. There is a distinct decrease in the nitrogen (all nitrogen atoms surrounding the silicon spiro center) and lone pair NBO occupancy down to 1.88 and 1.87 electrons in TISS***N***U-19 and TCTISS***N***U-23, respectively. This reduction in the lone pair occupancy on each of the nitrogen atoms, N_1_, N_5_, N_7_, and N_11_, around the spiro silicon atom Si_6_ is due to the donation of electrons mainly into the non-Lewis antibond orbitals σ*_Si6-N15,7,11_ (0.095 e^−^ on each). Moreover, the loss of occupancy on the cyano nitrogen (1.97 electrons) is caused by the donation of charges into the localized antibonding NBOs σ*_Si3-C≡16,18_ (0.05 e^−^ on each).

Before analyzing the consequences of the fluorine substitution on the donor–acceptor interactions, in OITSSHC-33, a few remarks should be addressed. As was alluded to in the introduction, the replacement of a carbon by an isostere, like silicon, in pharmacologically active agents usually has a marked impact on its pharmacodynamics and pharmacokinetics. Tacke and his research group [5] have thoroughly investigated this interesting isosteric substitution and its relevance for developing new classes of drug-active ingredients with higher efficiency than their carbon counterparts. Moreover, in numerous papers, it was shown that the C-Si-N bonding arrangement possesses a distinctive biological activity and chemical reactivity [68,69]. Provided that the new category of sila-/germa-spirocyclic imines possess the predicted pharmacological activity, the attachment of a unique substituent, like fluorine, with its small size and high electronegativity to these compounds would add some novel physicochemical properties comprising increased lipophilicity, charge mobility, and affinity for interacting with adjacent molecular entities. The main rationale for a successive fluorine addition (Table 10) is to systematically investigate the gradual impact of such systematic substitution on the internal donor–acceptor interactions and charge distribution along the framework of a spirocyclic imine unit. Additionally, it can be anticipated that the incorporation of fluorine substituents in an SCI unit will lead to the rearrangement of the electrophilic and nucleophilic centers, geometrical alterations, and the influence of the charge transport mobility and ability for docking efficacy to other molecular assemblies. The impact of fluorination on charge transfer dynamics and its efficiency across benzonitrile-based self-assembled monolayers has been investigated [46]. 

To gain more a pertinent rationalization of the fluorine substituent effect on the spirocyclic imines, a full NBO analysis was conducted, and the emerging delocalization energy values as estimated by the second-order perturbation theory (SOPT) are listed in Table 10. It is worth indicating that further representatives of the sila-/germa-spirocyclic imine series have been also fluorinated and analyzed by means of the NBO routine but for the sake of brevity, they have not been included in this paper. Rather, the focus has been placed on discussing the effect of the multiple fluorine substitution in SCIs by choosing OITSSHC-33 as an example for all other representatives of this class of compounds. An inspection of Table 10 reveals that the perturbative stabilization energy E(2) for a cross-hyperconjugative interaction symbolized by the π_(2)N1-C2_ → σ*_Si6-N7,11_ donor–acceptor interaction amounts to 3.1 kcal mol^−1^ in OITSSHC-33 and is slightly higher in the fluorinated derivatives, indicating a tendency for the enhancement of this kind of interaction along with the increase in the attached fluorine atoms. Furthermore, in accordance with the expectation of the strong polar Si-N= bonds with a high charge concentration on the nitrogen atoms, a donation of electrons into the unoccupied non-Lewis NBOs of the adjacent =C-F antibonds was made. Remarkably, however, are the particularly large delocalization energy values of 14.6 kcal mol^−1^ (and a value for the off-diagonal element in the NBO Fock matrix, F_i,j_ of 0.098 a. u.) in (**III**) and 14.0 kcal mol^−1^ in (**IIV**) for the σ_Si6-N1,5_ → σ*_C2,4-F_ donor–acceptor NBO interaction. Similarly, appreciable stabilization energy values of 15.9, 15.4, and 15.1 kcal·mol^−1^ (and F_i,j,_,= 0.100 a.u. for each) are affiliated with the donor–acceptor NBOs σ_Si6-N7_ → σ*_C8-F_ in (**II**), **(III**), and (**IV)**, respectively (Table 10). The reason for such a large E(2) value is the accentuated electron withdrawal by the fluorine atoms. Probably, the most decisive valence shell Lewis and non-Lewis charge delocalizations are those in which the lone pairs on both imine nitrogen and fluorine atoms are participating. For instance, the significant deviation from the idealized Lewis occupancy is related to the valence lone pair NBO (LP)_N7_ (and equally the valence lone pairs on the imino nitrogen atoms N_11,18,21_), which is occupied by 1.879 electrons in OITSSHC-33 and 1.832 e^−^ in the fluorine derivatives F4-, F8-, and F12- (Figure 4). The depletion of occupancy is due to charge distribution into the acceptor NBOs 6*Si_6_-N_7,21_ (each occupied by 0.103 e^−^), 6*C_8,20_-F (each occupied by 0.128 e^−^), and 6*C_8_-Si_9_ (0.094 e^−^). One further interesting charge delocalization occurs in F12-OITSSHC-33, where the occupancy of the valence (LP_3_)_F23_ NBO decreases to 1.945 e^−^, losing charges to the acceptor non-Lewis NBOs 6*Si_3_-C_2,4_ and 6*Si_3_-F_23_. The latter donor–acceptor interactions (LP_3_)_F23_ → 6*Si_3_-F_24_ (and obviously (LP_3_) _F24_ → 6*Si_3_-_F23_) indicate that the geminal terminal Si-F bonds are stabilized by means of a negative hyperconjugation effect with an appreciable stabilization energy E(2) of 11.8 kcal mol^−1^ (Table 10). This table also shows that in F12-OITSSHC-33, only moderate energy of 3.9 kcal·mol^−1^ is required for the delocalization of charges from the σ_C2,4-Si3_ donor NBO to the acceptor antibonding σ*_Si3-F_ NBO. 

These peculiar details, which have been derived from the NBO and SOPT analyses, confirm once more the relevance of the Si-N bond for modifying the fundamental physicochemical properties of molecular assemblies by incorporating such a vital bond. As can be anticipated, the successive addition of fluorine substituents to SSCIs forming either a C-F or Si-F bond as in OITSSHC-33 most likely will contribute to a drastic alteration or accentuation of most of the physicochemical properties that have been repeatedly addressed above. Furthermore, the partial or total fluorination of a sila-/germa-spirocyclic imine framework would promote their ablation capability on surfaces of functional carriers and manipulate the interfacial interactions of such functionalized surfaces. 

To examine the response of the charge distribution in spirocyclic silaimines (SCSIs) to fluorination, an NBO analysis was conducted on various fluorine derivatives of TSISS***N***U-19 (Figure S7). A review of the most prominent Lewis-occupied NBOs and non-Lewis-localized NBOs in F8-TSISS***N***U-19 (Table S19) leads to the following brief conclusions. The charge delocalization π_N1=Si2_ → 6*_Si6-N7_ is stabilized by 5.0 kcal mol^−1^, confirming the role of the cross-hyperconjugation in this class of SCSIs. Mainly by virtue of the strong electron withdrawal by the attached fluorine atoms, the occupancy of the Si_2_-Si_3_ bonds drops down to 1.856 e^−^ (at the B3LYP-aug-cc-pVDZ level of theory), as shown in Table S19. This perceptible departure from the idealized Lewis occupancy is provoked by the donation of charges into the acceptor antibond orbitals 6*_Si2-F_ and 6*_N1-Si2_ that are associated with delocalization energies of E(2) = 10.5 kcal mol^−1^ and 9.9 kcal mol^−1^, respectively. 

In accordance with the expectation, the NBO routine has also shown that the lone pairs on nitrogen and fluorine donate electrons into various non-Lewis antibond NBOs, like, for instance (LP)N_1_ → 6*_N5-Si6_, with a delocalization energy of (E(2) = 10.2 kcal·mol^−1^ and (LP_3_)F_15_ → 6*_Si8-N7_ (E(2) = 14.3 kcal·mol^−1^), leading to a reduction in the occupancy of the (LP)N_1_ NBO down to 1.843 e^−^ and the occupancy of the (LP_3_)F_15_ to 1.935 e^−^ (Table S19). Of particular interest is the donation of charges from the occupied (LP_3_)F_17_ NBO into the non-Lewis NBO 6*_Si3-F18_ (and similarly the delocalization of charges from (LP_3_) F_18_ into 6*_Si3-F17_), resulting in a loss of occupancy of the (LP_3_)F_17_ NBO (1.946 e^−^ in Table S19) with a stabilization energy E(2) of 10.3 kcal mol^−1^. These mutual donor–acceptor NBO interactions symbolize, once more, the negative hyperconjugative stabilizing effect between the geminally substituted fluorine atoms. For comparison reasons, the NBO occupancy values for the counterpart TISS***N***U-19 have been included in Table S19. A brief comparison of the data in this table reveals that the Si_2_-F bond in F8-TSISS***N***U-19 is clearly more polar than the C_2_-F bond F8-TISS***N***U-19, which is apparent from the polarization coefficient values of ξ(Si_2_) = 0.364 and ξ(F) = 0.932 in the Si_2_-F bond and ξ(C_2_) = 0.517 and ξ(F) = 0.856 in the C_2_-F bond. Moreover, it is interesting that the terminal Si_3_-F bond is equally polar in both F8-TSISS***N***U-19 and F8-TISS***N***U-19, which is discernible from the equal polarization coefficients of Si_3_ on one hand and the fluorine atom in the Si_3_-F bond on the other hand.

Finally, it is worth noting that some caution is advised when trying to derive decisive conclusions from comparing perturbative E(2) values originating from quantum chemical calculations carried out by applying different methods and basis sets. In this respect, a full NBO analysis on TISS***N***U-19 and TIGS***N***U-19 was performed by varying the computational method and using the same basis set to verify the effect of the applied method on the NBO occupancies and the donor–acceptor second-order stabilization energy E(2). Furthermore, the same computational method but in combination with different basis sets (with and without augmentation with diffuse functions) was applied. Table S20 visualizes the dependency of the E(2) values on the applied method and basis set for some important donor–acceptor interactions in the above-indicated SCIs. In Table S20, it was found that (1) using the B3LYP method in combination with basis sets aug-cc-pVDZ and cc-pVDZ suggests E(2) values that are by 6–13% larger, as provided by using the former basis sets than those that were obtained by employing the non-augmented basis set cc-pVDZ, demonstrating the effect of the inclusion of diffuse functions and, therefore, the variation of the basis set. (2) A comparison between the E(2) values, as predicted by B3LYP and B2PLYP in combination with the basis set aug-cc-pVDZ, provides an increase in the E(2) energy values by 7–21% when applying the double hybrid functional method, certifying the effect of the applied computational method. (3) In the final step, the wave function HF/6-311G(d, p) was used to inspect the consequence of the variation of both the utilized computational method and the applied basis set. In this case, the alterations of the proposed perturbative delocalization energy E(2) values in comparison to those resulting from the above-mentioned methods were nonuniform (some are smaller and others are larger), as is apparent in Table S20. Additionally, the calculated E(2) values for TIGS***N***U-19 exhibit the same trend as those that were found in TISS***N***U-19 by utilizing the same level of theory, but they are generally smaller (Table S20).

## 7. Frontier Molecular Orbital (FMO) Analysis

### 7.1. HOMO- LUMO Energy Gap

**Figure 7:**
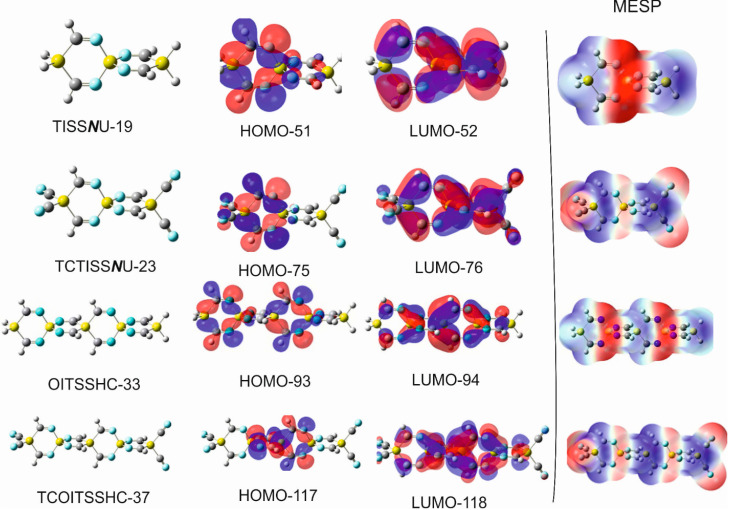
A comparison between frontier molecular orbitals HOMO and LUMO of the sila spirocyclic imines TISSNU-19 and OITSSHC-33 and their tetracyano derivatives. The right column shows the corresponding molecular electrostatic potentials (MESPs).

The FMO analysis [70] has disclosed that in OITSSHC-33 (and all other symmetrical SCIs), the HOMO and LUMO orbitals are uniformly distributed along the entire spirocyclic scaffold (Figure 7). This finding reflects the spiro-conjugative character of SCIs and indicates the facility of charge shuttling along the spiro molecular system, which, in turn, explains the essential role of SCIs for intra- and inter-molecular charge transfer. Obviously, the substitution of the SCIs with strong electron-withdrawing and/or electron-accepting substituents (e.g., F, C≡N) has a decisive effect on the distribution of the highest occupied and lowest unoccupied HOMO and LUMO frontier molecular orbitals. Conspicuously, while the LUMO wave function is extended all over the SCI framework in terminally substituted entities and perfluorinated OITSSHC-33 (like the non-substituted parent molecules), the HOMO wave function is consistently restricted to definite regions on the SCI skeleton (Figure 7 and Figure S13). 

**Figure 8:**
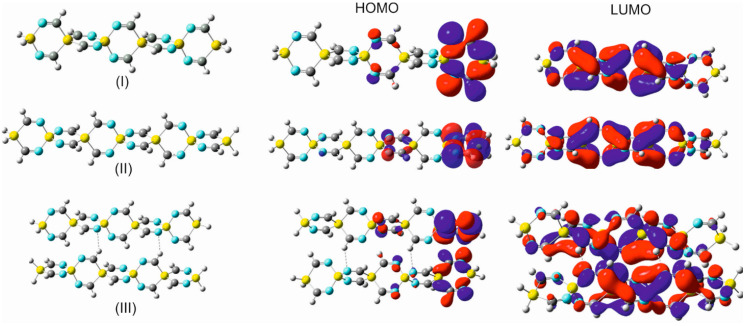
(**I**) 2,4,8,10,14,16,20,21,24,25-decaimino-3-18-disila-6,9,12,15,tetrasilaspiro [5.2.2.2.515.212.29.26]hexacosane (DITSSHC-40). (**II**) 2,4,8,10,14, 16,20,22,25,26,29,30-dodecaimino-3,21-disila-6,9,12,15,18-pentasilaspiro [5.2.2.2.2.518.215.212.29.26]hentriacontane (DODIPSSHT-47). (**III**) Dimer of (DITSSHC-40).

In the carbo spirocyclic imine, however, both the HOMO and LUMO orbitals are uniformly distributed along the spiro scaffold (Figure S14). In this respect, a structural analysis of the dimer of DITSSHC-40 has been performed, revealing that this dimer is stabilized by the formation of an inter-molecular nitrogen–hydrogen bond (N····H bond), as shown in Figure 8. 

The subsequent FMO analysis found that the computed HOMO and LUMO frontier orbitals show a peculiar “crowd” of LUMO orbitals and terminally localized HOMO orbitals (Figure 8). This preliminary inspection of the expected inter-molecular attractive interactions between stacked SCI units will be intensified and extended to comprise larger SCI units with molecular stacking aggregates consisting of multiple layers (stabilized via hydrogen bonding or attractive π-π interactions), enabling three-dimensional charge transport. One further conceivable application for SCI stacking materials could be their utilization for coating surfaces and modifying their electronic properties and interfacial chemistry. 

The main objectives of the frontier molecular orbital (FMO) approach are to focus on the local reactivity centers, the highest occupied molecular orbital, HOMO, and the lowest unoccupied molecular orbital, LUMO, of a molecular system instead of invoking the total electron density and the molecular electrostatic potential. The FMO theory furnishes insight into the consequences of the interaction between the nucleophile donor HOMO orbitals and the electrophile acceptor LUMO orbitals and, thus, into the chemical reactivity of the reactants. The HOMO-LUMO energy gap (E_gap_) is the most crucial parameter for the prediction of the chemical reactivity of a certain chemical entity. 

The magnitude of the HOMO-LUMO energy gap in a single molecule (e.g., a silaspirocyclic imine) represents the barrier for charge transfer within such a molecule and, therefore, determines its optical properties and chemical reactivity. Furthermore, the variation of the HOMO-LUMO energy gap upon substitution by an electron-donating or -accepting substituent provides valuable information about the impact of substituent effects and their contribution to lowering (smaller energy gap) or elevating (larger energy gap) the activation energy of a chemical reaction or the absorption of photons. 

On this background, the HOMO and LUMO frontier molecular orbitals of some representatives of the discussed spirocyclic imines and their cyano- and fluoro-derivatives were calculated by applying the B2PLYP/aug-cc-pVDZ functional method. The FMO energy values, as well as the E_gap_ values, are shown in Table 11, and the corresponding three-dimensional mapping of the HOMO and LUMO orbitals are illustrated in Figure 7 and Figure S13. 

Before discussing the results, which were obtained from the FMO theory, two essential remarks should be pointed out. (1) All E_gap_ values and therefrom-derived descriptor values (see details below) are valid for a single isolated molecule (intra-molecular HOMO-LUMO orbital interactions), which implies that these values are only applicable to molecular entities in the gas phase. (2) As soon as the condensed phase is under consideration, mutual electronic interactions comprising protic and aprotic solvent effects (polarization effects including distortion of the charge density distribution, hydrogen bond formation, crystal lattice-packing forces, etc.) would substantially affect the E_gap_ energy and lead to more complicated molecular interactions, which are no more describable by the formalism and equations that have been presented by the FMO theory.

In Table 11, it is apparent that the successive addition of fluorine substituents to OITSSHC-33 leads to a gradual increase in the E_gap_ value. For instance, this value rises by 1.63 eV (37.59 kcal·mol-1 on moving from OITSSHC-33 to its F12- derivative). On the other hand, the terminal geminal substitution of the different sila- and germa-spirocyclic imines by fluorine or cyano groups seems to have negligible influence on the HOMO-LUMO energy gap (Table 11). 

To explore the dependency of the E_gap_ values on the utilized level of theory, the B3LYP/aug-cc-pVDZ method was chosen to calculate the energy gaps for various SCIs and compare the obtained results (Table 12) with those that were provided by applying the B2PLYP/aug-cc-pVDZ method.

This comparison unveils the following findings. (i) The variation of the E_gap_ values provided by the B3LYP/ag-cc-pVDZ method parallelled those that were suggested by the B2PLYP/aug-cc-pVDZ calculations. (ii) The E_gap_ values produced at the DFT/B3LYP/aug-cc-pVDZ level of theory are appreciably lower than those that were predicted by the B2PLYP/aug-cc-pVDZ method. (iii) Surprisingly, the substitution of the terminal hydrogens in TISS***N***U-19 for the heterocyclic fragments aziridine, azetidine, silole, or germole has little effect on the E_gap_ values. Additionally, the prolongation of the silaspirocyclic imine skeleton to molecular systems having six and eight rings with twelve and sixteen imino groups leads to a discernible lowering of the HOMO-LUMO energy gap, which ascertains the stability of these molecular systems due to the reduction in the barrier for the electrons to undergo internal HOMO-LUMO (donor–acceptor) interactions and shuttle along the spirocyclic scaffold. 

From the viewpoint of the FMO approach, strong electron donating or accepting substituents are anticipated to exert a noticeable effect on the donor/acceptor behavior of the spiro-conjugated SCIs. Depending on the chemical nature and the positions of the attached substituents and their impact on the HOMO-LUMO energy gap, it is likely that the HOMO of the donor possesses the same orbital symmetry as the acceptor LOMO orbital, and charge transfer (CT) occurs. Such cross-conjugated CT (internal HOMO-LUMO interaction) takes effect along the entire spiro chain. This unique kind of CT process was closely studied more than three decades ago by Maslak and his group [71].

To explore more thoroughly this apparent dependency of the computed HOMO and LUMO orbital energies (and consequently the energy gap E_gap_) on the employed level of theory, additional calculations were performed utilizing the commonly used exchange-correlation hybrid functional B3LYP and the wavefunction-based Hartree–Fock (HF) along with the Pople double and triple zeta basis sets (with diffuse function augmentation and without). Furthermore, the Møller–Plesset MP2 wave function in combination with the cc-pVDZ basis set has been used. The collected results are summarized in Tables S21 and S22. 

As is visible from these tables, the B3LYP functional method suggests (irrespective of the quality of the applied basis set) reasonable values for the energy gap E_gap_ and consistently negative E_LUMO_ values. Strikingly, however, whereas B2PLYP/cc-pVDZ predicts moderate E_gap_ values (Table 11), the HF wave function in combination with Pople’s double and triple zeta (also with diffuse functions), as well as the MP2 wave function along with cc-pVDZ and aug-cc-pVDZ basis sets, provide substantially larger (perhaps unrealistic) E_gap_ values (Table S21) than those that have been predicted by the B3LYPfunctional method. It is also noteworthy that the wavefunction-based methods, HF and MP2 (and partially the B2PLYP function), provided positive E_LUMO_ values (Table S21). 

The following few examples demonstrate this distinct dependency of the FMO energy values on the utilized computational method. The E_gap_ for OITSSHC-33 is predicted by B3LYP/aug-cc-pVDZ to be 3.7 eV (Table 12), while B2PLYP/aug-cc-pVDZ (Table 11), MP2/aug-cc-pVDZ, HF/6-311+(d,p), and B2PLYP-cc-pVDZ (Table S21) suggest 6.7, 10.4, 10.7, and 6.9 eV, respectively, for this energy gap. One further example is provided by TISS***N***U-19, where the aforementioned computational methods lead to the following E_gap_ values: 4.1, 7.1, 10.3, 10.7, and 7.4 eV. Based on these inconsistent results, it is worth emphasizing that caution is required when the calculated HOMO-LUMO energy gaps are considered for the deduction of crucial and reliable conclusions. One possible way for avoiding this confusion is to compare these computed values with those that have been gained from the experiment, e.g., by employing the UV-cyclic voltammetry technique [72], the determination of ionization potential (IP) [73], and electron affinity (EA) [74]. This conspicuous unpredictable dependency of the E_gap_ values on the applied computational method and basis set obviously leads to an appreciable uncertainty to a priori choosing the most appropriate combination of a quantum chemical method and basis set to obtain reliable E_gap_ values and, therefore, more meaningful descriptors derived from them. Various attempts have been made to achieve this goal and, among them, the proposal states that only those computational methods and basis sets that produce negative LUMO energy values are appropriate for the calculation of the E_gap_ values [75]. This dependency of the computed E_gap_ either on the applied quantum chemical method or/and the basis set has been the subject of controversy in many publications. One conceivable explanation for this discrepancy could be that the theoretical results in this respect also depend on the nature and electronic structure of the investigated class of molecules. Perhaps the unique spiro cross-hyperconjugated nature of the spirocyclic imines has led to the above-depicted behavior of the E_gap_ on the variation of the level of theory. 

Perhaps the most plausible rationalization for the appreciable dependency of the HOMO-LUMO energy on the chosen level of theory is that the energy required for moving an electron from an occupied molecular orbital to a virtual unoccupied orbital cannot be simply considered as the energy difference between these two orbitals. This is because moving an electron from an occupied molecular orbital to a virtual LUMO orbital results in a redistribution of the electrons and, thus, in different subsequent energy levels. The uncertainty by the determination of virtual LUMO orbital energy is particularly accentuated by using computational methods based on wave functions, like MP2 and double hybrid functions, regardless of the quality of the applied basis set, more than on applying classical density functional methods. This is because wave functions, in addition to basis sets augmented with diffuse functions, expand the electronic molecular space of a chemical entity, resulting in the generation of a vast number of excited states (virtual orbitals and Rydberg states), which makes the localization of a LUMO and LUMO+ energy levels quite uncertain. 

In this regard, a reasonable approach was presented by Schmidt et al. [76] to circumvent the uncertainty in calculating and localizing the virtual LUMO orbitals by introducing valence virtual orbitals (VVOs) instead of canonical orbitals. A straightforward scheme was applied to extract all valence-like orbitals from the large empty canonical orbital space leading to the VVOs. Eventually, it was shown that these valence virtual orbitals are practically independent of the employed basis set. 

### 7.2. Chemical Descriptors

Koopmans’ Theorem [77] and its further development by many others [78,79,80] have paved the way for the application of the ionization potential and the electron affinity for closed-shell molecular systems (which was later revised to be also applicable to ions and radicals) and their association with the HOMO and LUMO orbital energies. This concept has eventually helped to evaluate new effective chemical descriptors, which allow for estimating essential parameters affecting the chemical reactivity of a molecule.

The underlying formalism of Koopmans’ Theorem is based on the correlation between the highest occupied molecular orbital energy E_HOMO_, the lowest unoccupied molecular orbital energy E_LUMO_, and the ionization potential (**I**) and the electron affinity (**A**) of the form E_HOMO_ = −I and E_LUMO_ *=* −A. Accordingly, the energy gap between these frontier orbitals is E_gap_ = E_HOMO_ − E_LUMO_ ≈ (I) − (A). Based on this relationship between an electron-donating HOMO orbital and an electron-accepting LUMO orbital, the smaller E_gap_ is, the easier electrons transfer between molecular orbitals occurs. One essential consequence of Koopmans’ Theorem and its subsequent extension ultimately enabled the experimental determination of the HOMO-LUMO energy gap by measuring the ionization potential and the electron affinity, as was indicated previously in this work. Aside from that, it is perhaps of interest to indicate that in solid-state physics, the HOMO-LUMO energy gap correlates with the Fermi energy level (E_F_) by the equation = E_gap_/2. Based on the above-cited basic definitions, the following important descriptors for characterizing the readiness of a chemical species (molecule, ion, or a radical) for donating or accepting electrons (or the ability for polarization) have been derived:

(1) The global chemical hardness: η^−^ = I − A/2. The hardness of a molecular entity (comprising molecules, ions, and radicals) quantifies its resistance to polarization by an external electric field (e.g., the approach of a potential reactant). Accordingly, molecular entities with large HOMO–LUMO energy gaps are hard since the barrier for their electron density redistribution is relatively high (E_gap_ =2 η). In contrast, molecular systems with a small HOMO–LUMO energy gap (small excitation energy) are easily polarizable and, therefore, are designated as soft. Softness is defined as the reciprocal of the hardness (*S*) = 1/η, and it mirrors the reactivity of these systems. Moreover, a donor atom with low polarizability and high electronegativity is called a “hard base”, and a donor atom with high polarizability and low electronegativity is called a “soft base”. Similarly, an acceptor atom with a highly positive charge and small size (and does not have easily excited outer electrons) is denoted hard acid. Furthermore, an acceptor atom with a low positive charge and large size (and has several easily exited outer electrons) is termed soft acid. Based on these definitions, it can be concluded that hard acids prefer to coordinate with hard bases and soft acids with soft bases [78], 

(2) The molecular electronegativity, χ = I + A/2, is defined as the first derivative of the total electronic energy, E*,* with respect to the number of electrons, *N* (χ) = −(∂*E/*∂*N*)_Z_ (the nuclear changes Z are kept fixed). According to Mulliken, χ is related to the ionization potential, I, and electron affinity, A [81]. Molecular electronegativity (in terms of Pauling’s definition of electronegativity) is the tendency of molecules to attract electrons.

Based on the above-indicated relationship between the molecular electronegativity and the ionization potential and electron affinity, the experimental determination of I and A (and the comparison of their values with the calculated HOMO and LUMO energies) allows for the evaluation of more reliable value for the electronegativity, χ. Additionally, the χ value is a helpful tool for the recognition of Lewis acids (electron acceptors) and bases (electron donors) and their strengths.

(3) The electronic chemical potential: μ = −(I + A/2). The electronic chemical potential, μ, is defined as the affinity of an electron to escape and is defined as the first derivative of the total energy with respect to the number of electrons in a molecule: μ = −(∂*E/*∂*N*)_z_ (the nuclear charges Z are kept fixed) [79]. As was indicated above, negative χ is equal to the electronic chemical potential, μ (μ = −χ), and, according to Parr and Pearson [78], the molecular electronegativity, χ, and the electronic chemical potential, μ, are interrelated to the chemical hardness according to the relationship 2η = (∂*μ/*∂*N*)_z_ = −(∂χ*/*∂*N*)_z_ = (∂^2^*E/*∂*N^2^*)_z_. It is also worthwhile to note that in contrast to the global chemical hardness, which differs from one atom to the other, the electronic chemical potential is consistent everywhere in a molecule. Furthermore, the chemical potential (μ) is also the driving force for electrons to transfer from a molecular species of low electronegativity (high chemical potential) to another species of higher electronegativity (low chemical potential). It is of interest to point out that the chemical potential and the absolute electronegativity are molecular properties and not orbital properties [82]. However, based on the Koopmans’ Theorem and the relationship between the ionization potential, I, and the HOMO energy on one side and between the electron affinity, A, and the LUMO energy on the other side, a quantitative connection between the molecular and the orbital properties has been established. 

(4) The global electrophilicity index (GEI), which is defined as ω = μ^2^/2η. This is perhaps the most meaningful descriptor that was introduced by Parr et al. in 1999 [79]. Due to these authors, although the GEI and the electron affinity, A, are related to each other since both ω and A indicate the capability of a molecular entity to attract electrons, the electron affinity, A, mirrors the ability to accept precisely only one electron from the environment, whereas the global electrophilicity index, ω, defines the energy lowering due to maximal electron flow between donor and acceptor. Additionally, the GEI furnishes information related to the molecular hardness and chemical potential and, thus, reflects the resistance of a molecular unit for donating electrons. Roughly speaking, all the above-defined descriptors are important in determining the amount of energy required to add or remove electrons in a molecule.

Electrophilicity describes the capability of an electrophile to accept extra electronic charges and the inability to donate charges and, thus, supply information about charge exchange and the stability of a molecular entity and, therefore, represent the chemical potential and the hardness associated with it. In this respect, the GEI, (ω), estimates the energy drop resulting from charge transfer between donor and acceptor orbitals derived from HOMO–LUMO energy gap values. From this perspective of consideration, it follows that the GEI reflects the charge flow between interacting moieties and, consequently, the reactivity and modulation of their chemical properties. It is worth noting that the combination of the FMO results (including the chemical descriptors) with the experimentally obtained results is a helpful strategy for the recognition of the reactivity centers of a molecular species [83]. Table 12 summarizes the computed values of the chemical descriptors for a variety of SCIs and some of their derivatives that were obtained from the FMO analysis. Based on the above-introduced definitions of the frequently applied quantum chemical descriptors for rationalizing some crucial factors affecting the chemical and biological activity of molecular species, the following conclusions could be derived. 

The highest softness value, S, of 0.570 (inverse of the hardness value of 1.755) is exhibited by DODIPSSHT-47. One plausible elucidation for this high softness (easily polarizability and low E_gap_: S = 2/E_gap_) is that this large spirocyclic imine, consisting of six silaspiro rings with twelve electron-rich imino groups, is likely an indication for the cross-spiro-conjugation and the spiro-aromaticity of this cyclic imine, as will be discussed later in this work. In contrast, the lowest global softness (highest global hardness) of 0.381 and 0.389 is presented by F8-OITSSHC-33 and F12-OITSSHC-33, respectively. These values symbolize the resistance of such molecular entities to losing electrons. Support for this conclusion is provided by the most negative values of the chemical potential of −5.43 and −5.84 (and, correspondingly, by the largest molecular electronegativity values), as well as by highest global hardness values, as is apparent in Table 12. All these values concertedly imply that these fluorine derivatives of SCIs are regarded to be hard Lewis acids and, consequently, they resist the release of electrons and, hence, are quite stable (they possess the largest E_gap_ values). This is not particularly surprising because the accumulation of fluorine atoms in such an electron-rich type of molecule enhances their stability.

Table 12 also reveals that the largest values of the global electrophilicity index (GEI) are primarily manifested in those spirocyclic imines that are terminally substituted by electronegative fluorine atoms or cyano groups. Accordingly, for instance, the tetracyano derivatives of TCTISS***N***U-23, TCTISS***C***U-23, and TCTIGS***N***U-23 possess large GEI values of ω = 7.1, 8.5, 6.7. Additional examples are *term*-F4-OITSSHC-33, F8-OITSSHC-33, and F12-OITSSHC-33, where ω = 6.1 for *term*-F4-OITSSHC-33 vs. 5.6 and 6.6 for the latter two fluorine derivatives. These large GEI values (which are associated with the chemical potential and E_gap_: ω = μ^2^/E_gap_) evidently affirm the pronounced electrophilic nature of these compounds. Another striking example is presented by 3,9-*tetra*-azetid-TISS***N***U-19 in Table 12. This molecule possesses quite low hardness, η, of 1.8 (softness S = 0.56) and a similarly low global electrophilicity index, ω, of 2.9. From these values, it can be inferred that this molecular entity is ready to release charges, indicating its nucleophilicity. 

It is also worth pointing out that in Table 12, the terminal incorporation of heterocyclic groups, i.e., aziridine, azetidine, silole, and germole in the TISS***N***U-19 scaffold leads to a lowering of the HOMO-LUMO energy gap, ranging from 0.11 eV to 0.47 eV, which obviously contributes to a corresponding variation of the chemical descriptors in Table 12. All these examples substantiate the impact of introducing substituents with different electron affinities on the E_gap_ and, consequently, the chemical descriptors derived from it. This, in turn, affects the charge distribution and charge transfer within the considered cyclic imine framework, as well as their readiness for charge exchange, which eventually determines their chemical and biological reactivities. 

One final note with respect to the discussed chemical descriptors. Obviously, the apparent dependency of the E_gap_ on the applied quantum chemical method and basis set, which has been addressed above, disseminates throughout all these descriptors simply because they have been derived from the HOMO-LUMO orbital energies (and subsequently from the ionization potential, (I), and electron affinity, (A)). They furnish additional details, supporting the above-addressed results of the FMOs analysis, and 3D contour maps of the molecular electrostatic potential (MESP) on the total electron density surface for a selection of spirocyclic imines were calculated by employing the B2PLYP/aug-cc-pVDZ computational method.

## 8. Molecular Electrostatic Potential (MESP)

For mapping the MESP, a GaussView 6.0.16 graphical interface was utilized [26].

Whereas the NBO analysis, the FMO theory, and the HOMO-LUMO orbitals describe the local distribution of electronic charges and charge transfer between donors and acceptors orbitals, the molecular electrostatic potential (MESP) supplies crucial information related to the total three-dimensional charge distribution isosurface around the sphere of a molecular system. Moreover, MESPs (which can be regarded as a “snapshot” of the total charge distribution) provide direct indications regarding the ionicity or covalency of bonds in molecules, as well as qualitative knowledge about the electronegativity strength of the atoms forming them. 

MESP mapping has proven to be decisively important for drug discovery and biochemistry to understand the mutual interaction between receptors and substrates forming receptor-substrate complexes (e.g., protein-receptor/enzyme-substrate).

The visualization of the electronic change distribution has become increasingly more sophisticated and easily feasible by means of developed computational routines and graphical illustrations using color coding, which allows for a transparent and expedient interpretation of the driving forces for the reactivity of molecular systems. Traditionally, the nucleophilic (high electron density) and electrophilic (low electron density) domains of an MESP isosurface are assigned to red and blue colors, respectively. Moderate nucleophiles or electrophiles appear on this color scale as reddish or blueish and neutral zones (e.g., covalent bonds turn up greenish). This scheme of applying color coding for the qualitative interpretation of the MESP and specifying the nucleophilic and electrophilic sites in a molecular assembly is quite helpful and represents a simple means for predicting the chemical reactivity of molecules. A vast number of papers have been published over the last five decades dealing with the theoretical and experimental investigation of the MESP and its relevance for chemistry and applied sciences in general [84,85,86,87,88]. 

Based on the above-portrayed details about the MESP and its relevance for the elucidation of electronic charge distribution in a molecular sphere, the calculated molecular electrostatic potentials for a selection of SCIs will be hereafter presented and closely discussed. 

**Figure 9:**
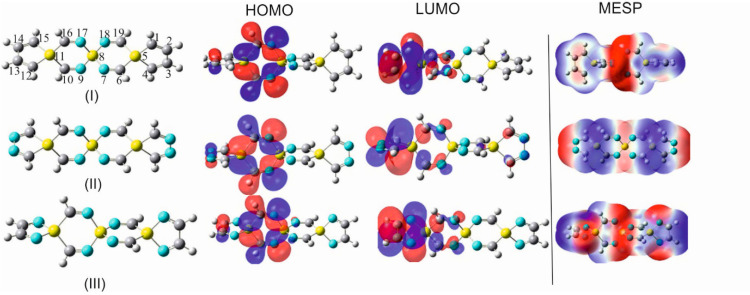
A comparison of the HOMO and LUMO frontier orbitals and the molecular electrostatic potentials (MESPs) of terminally incorporated silole and diminosilole in the TISSNU-19 scaffold (N-Si-N spiro center): (**I**) 6,10,16,19-tetraimino-5,8,11-trisilaspiro [4.2.2.411.28.25]nonadeca-1,3,12,14-tetraene (TITSSNDTE-31). (**II**) 1,4,6,10,12,15,16,19-octaimino-5,8,11-trisilaspiro [4.2.2.411.28.25]nonadecane (OITSSND-***D***-27). (**III**) 2,3,6,10,13,14,16,19-octaimino-5,8,11-trisilaspiro [4.2.2.411.28.25]nonadecane (OITSSND-***V***-27). For these abbreviations, see the text.

**Figure 10:**
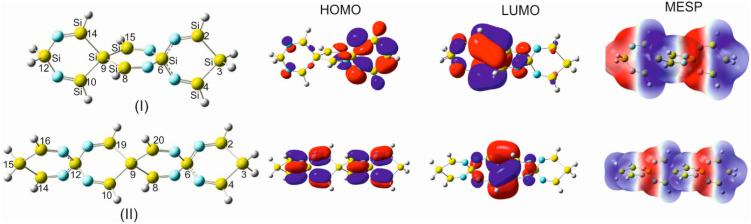
A comparison between the HOMO and LUMO frontier molecular orbital FMOs of (**I**) 2,4,8,10,14,15-hexasilaimino-3,12-disila-6,9-disilaspiro [5.2.59.26]hexadecane (HSIDSSNSH-26). (**II**) 2,4,8,10,14,16,19,20-octasilaimino-3.15-disila-6,9,12-trisilaspiro [5.2.2.512.29.26]henicosane (OSITSSHC-33). To the right, the corresponding MESPs are displayed.

In the MESP mapping images (Figure 7, Figure 8, Figure 9 and Figure 10 and Figures S13–S16), it is apparent that the partitioning of the negative (charge concentration) and positive (charge depletion) charges results in the visualization of marked nucleophilic (nitrogen atoms incorporated in imino groups) and electrophilic (spiro centers and hydrogen atoms) zones. Figure 7 and Figure 8, Figures S13 and S15 display the three-dimensional MESP mapping of various spirocyclic imines (SCIs) that have been chosen to visualize the electronic reasons, leading to the variation of charge distribution upon substitution of these SCIs with strong electronegative moieties (-C≡N and F) and heterocyclic fragments. The MESPs of these spirocyclic imines indicate the existence of two distinguishable regions: intensive red, indicating high electron density, and intensive blue, manifesting the lack of charges. The segments with a gradual color transition of pale red and pale blue characterize the balance between positive and negative surface potentials. 

It is worth noting that the illustrated MESPs of the presented molecules are chosen to cover the range from −0.007 au with the highest charge density (lowest electrostatic potential energy, marked red) to 0.009 au with the lowest charge density (highest electrostatic potential energy, marked blue). 

As is clearly demonstrated by the above-cited figures, the periodic occurrence of the nucleophilic and electrophilic domains along the spiro scaffold are adequately visualized by the MESP mapping of the electron density distribution surface.

Figure 7 depicts the MESP mapping onto the total electron density surface for TISS***N***U-19 (with N-Si-N spiro center), OITSSHC-33, and their terminally substituted tetracyano derivatives, TCTISS***N***U-23 and TCOITSSHC-37. From these MESPs representations, the following decisive conclusions can be derived. (i) The accentuated red sites around the imino nitrogen atoms in TISS***N***U-19 and OITSSHC-33 exhibit the highest charge density and lowest electrostatic potential energy within these molecules. The sites with high charge concentration make these silaspirocyclic imines accessible for an electrophilic attack. (ii) The blue-colored zones indicate the positively charged silicon spiro centers, which are potentially ready for a nucleophilic addition reaction. The peripheric blue-colored areas show the charge deficiency on the silicon and hydrogen atoms, whereas the light reddish region shows the residual negative charges on the carbon atoms after imparting partial charges to the neighboring nitrogen atoms. (iii) The MESPs of these spirocyclic imines (and higher homologs) generally illustrate the simultaneous existence of two discriminable regions, nucleophilic and electrophilic. This clear partitioning of charges within these spirocyclic imines imparts them form the previously described bifunctional character and, therefore, entitles them to act either as a Lewis base or a Lewis acid, depending on the approaching reactant. (iv) Figure 7 evidently demonstrates the distortion of the charge density distribution within the molecular electrostatic potentials (MESPs) of TCTISS***N***U-23 and TCOITSSHC-37 due to the strong electron-withdrawing nature of the geminally substituted C≡N groups. The appearance of the red color at both ends of the spiro scaffold and the partial fading of the internal color coding symbolizes the effect of the strong charge, attracting cyano groups. In the MESPs in Figure S13, it is evident that attaching strong electronegative substituents, like fluorine, at different positions along the OITSSHC-33 spiro framework obviously results in a decisive charge redistribution within these molecular assemblies and, thus, in a drastic change of the internal nucleophilicity and electrophilicity of the spiro unit. As is visible from the displacement and intensity alteration of the red and blue color-coding within the MESP mapping, depending on the positions and number of the introduced fluorine atoms, the electron density distribution varies correspondingly. Additionally, in Figure S13, it can be concluded that the withdrawal of electronic charges by the terminal fluorine substituents leads to an appreciable increase in the charge concentration (intensive red color) on the fluorine atoms and, consequently, to a depletion of charges on the imino groups (fading red color). Such an internal electronic shift enhances the ability of the SCI entity for docking at electrophiles on both ends. Additionally, the successive increase in the number of fluorine substituents causes an additional shift of the color coding within the MESP isosurface plots, indicating a larger distortion of the electron density distribution and, therefore, an increase in the reactivity of the substituted spirocyclic imine. 

Furthermore, the terminal substitution of spirocyclic imines with the heterocyclic aziridine, azetidine, silole, and germole has led to a drastic distortion of the MESP (Figure S16) and, consequently, sizeable structural alterations (as has been alluded to above and shown in Table 12) of the spiro skeleton, e.g., a departure from planarity of the fused rings and their perpendicular arrangement, the revocation of the rigidity of the spiro scaffold, and the subsequent incidence of conformational flexibility. 

Hereafter, only the content of the remaining MESP figures will be cited. Figure S15 visualizes the MESP mapping of the carbospirocyclic imine OITCSHC-33 (as well as the representation of the HOMO and LUMO frontier orbitals), demonstrating the loss of symmetry caused by the insertion of carbon atoms as spiro centers for the reasons that have been already discussed earlier in this work. 

Figure S17 shows (for comparison reasons) the MESP representation of terminally incorporated germole and diamino-germole fragments into the TIGS***N***U-19 scaffold with germanium atoms as spiro centers (in addition to the corresponding HOMO and LUMO orbitals). Figure 9 illustrates the MESP mapping of terminally incorporated silole and diamino-silole fragments into the TISS***N***U-19 scaffold (in addition to the corresponding HOMO and LUMO orbitals).

Figure 10 shows the MESPs of two silaspirocyclic silaimines HSIDSS***NSi***H-26 (displaying a spirocyclic silaimine unit with asymmetrical charge distribution and electrophilic and nucleophilic ends) and OSITSSHC-33.

## 9. AIM Analysis

The topological properties of the charge density distribution in the selected examples of spirocyclic imines have been analyzed by applying the quantum theory of atoms in molecules (QTAIM), which was developed by Bader and others [21,22,23,24,89].

In numerous papers, it has been repeatedly demonstrated that by employing Bader’s AIM theory, valuable information about bonding properties in molecules and the reasons for their structural stability can be acquired. In addition, changes in the charge density distribution, ρ(r_c_), provoked by substituents, are mirrored by the fluctuation of the Laplacian of the charge density, ∇^2^ρ(r_c_) (which is the algebraic sum of the three eigenvalues, λ_1_, λ_2_ λ_3_ of the charge density at a bond point), which allows for a three-dimensional representation of these effects, which is crucial in the case of SCIs.

In the AIM analysis, a positive value of the Laplacian, the small value of ρ(r_c_) at the bond critical point (*bcp*), implies that charges are concentrated in the separate atomic basins rather than the internuclear region, which means that electronic charges are locally depleted at *bcp,* indicating the occurrence of closed-shell interactions, such as in ionic or hydrogen bonds. Covalent bonds display a negative value of ∇^2^ρ(r_c_) at the *bcp,* signifying a concentration of electron density at or close to a *Bcp*. r_A_ and r_B_ are the distances between the nuclei A and B and the A–B bond critical point, (*bcp)*_A–B_. The ellipticity *ε_c_* is defined as ε_c_ = λ_1_/λ_2_ – 1, and it describes the deviation of a bond at (*bcp*) from the cylindrical symmetry. ε_c_ is crucial for characterizing chemical bonds, conjugation, hyperconjugation, and aromaticity. For example, ε_c_ for C-C and C≡C is equal to zero. ε_c_ for C=C in ethene is substantially ≠ 0 (in this case, ε_c_ = 0.4 at HF/6-31G*level of theory), and ε_c_ = 0.07 for the C-C single bond between the two double bonds in butadiene, signalizing the conjugation of the two π-bonds in butadiene and, thus, the deviation from the cylindrical symmetry [23]. Some of the results that were obtained from Bader’s QTAIM analysis for a variety of silaspirocyclic imines are shown in Table 13 and Tables S23–S26. Moreover, to investigate the effect (fingerprint) of substituents in terms of the criteria of the QTAIM theory, topological properties, tetracyano, and a variety of fluorinated derivatives of OITSSHC-33 have been closely investigated at the B2PLYP/aug-cc-pVDZ level of theory.

In Table 13, it is apparent that in OITSSHC-33, the large negative value (−0.909 e/$a_{0}^{5}$) of the Laplacian density at the (*bcp*) and the substantial deviation of the ellipticity ε_c_ from zero for the N_1_=C_2_ bond demonstrate the covalent character (the electron density is concentrated at the bond point) of this double bond.

In contrast, the silicon nitrogen bonds, N_1_-Si_6_ and Si_6_-N_7_, that are participating in the spiro center in OITSSHC-33 and inTCOITSSHC-37 are characterized by a large positive value of the Laplacian (0.599 e/$a_{0}^{5}$), confirming the ionic character of these bonds. This feature is also affirmed by the r_A_ and r_B_ values, as well as the ellipticity ε_c_ with its evidently deviant value from zero, indicating the formally partial π character of these bonds (Table 13). The nature of the C-H and Si-H bonds differ substantially by being a covalent and ionic (or strong polar) single bond (ε_c_ = 0), respectively, as is apparent from the large negative value (−0.993 e/$a_{0}^{5}$) of ∇^2^ρ(r_c_) for the former and positive small value (0.278 e/$a_{0}^{5}$) for the latter. 

Table 13 also shows that the substitution of the terminal hydrogens in OITSSHC-33 by cyano groups has little effect on the charge density distribution ρ(r_c_) and its Laplacian for all bonds. This finding is in harmony with the small variation of the structural parameters upon the addition of the cyano groups, as has been discussed above.

For attaining additional knowledge about the influence of the cyano substituents on the electronic structure of the silaspirocyclic imine framework, the topological properties of the charge distribution ρ(r_c_) in TCTISS***N***U-23 and TCOITSSHC-37 were compared with each other (Table 13 and Table S24). From the invariance of ρ(r_c_) (0.455 e/$a_{0}^{3}$) and the approximately equal values of the Laplacian ∇^2^ρ(r_c_) of 0.259 (Table 13) and 0.263 e/$a_{0}^{5}$ (Table S24) for the C≡N bond in these compounds, it is apparent that the degree of interaction between this substituent group and the SCI scaffold is independent of the size of the host SCI. Furthermore, it can be concluded that the influence of the terminal substitution by the geminal cyano groups is a local effect and targets only the adjacent bonds and angles.

Another discernible example is the Si-C≡ bond with its positive Laplacian of 0.403 e/$a_{0}^{5}$) and an ellipticity of 0.036 (Table 13). These values imply that this bond, which is part of the geminal Si(C≡N)_2_ moiety, is ionic or highly polar. The deviation of the ellipticity from the cylindric symmetry indicates the formation of a partial double bond between silicon and the cyanocarbon atom, leading to a strengthening of the Si-C≡ bond. Such a charge transfer is favored by the conjugative interaction between the geminal cyano groups.

Using B2PLYP in combination with the basis set cc-pVDZ produced almost the same values for the Laplacian at the *bcps* for these bonds, except for the double (N=C) and triple (C≡N) bonds in TCOISSHC-37. While the non-augmented basis set suggests −0.775 and 0.474 e/$a_{0}^{5}$ for the Laplacian at the bond critical points of the N_1_=C_2_ and C≡N bonds, the inclusion of the diffuse functions leads, however, to the values −0.887 and 0.259 e/$a_{0}^{5}$) (Table 13), respectively. The ellipticity values of both bonds remain invariant by the expansion of the basis set, i.e., 0.088 for the N_1_=C_2_ bond, certifying the double bond π-character of this bond and the almost vanishing value of ε_c_ = 0.03 for the C≡N bond, confirming the cylinder symmetric distribution (the principal curvatures λ_1_ and λ_2_ are almost equal) of the charges in the C≡N triple bond. The directionality of the ellipticity toward the π bond is given by the major axis -λ_2_, which is the smallest of the two negative eigenvalues λ_1_ and λ_2_.

For additional verification of this apparent dependency of the topological properties on the variation of the quality of the basis set, an AIM analysis was conducted on OITSSHC-33 (Table S23) and TCOITSSHC-37 (Table S25), employing People’s split-valence basis sets with polarization functions and diffuse functions (and without) of the types 6-311 + G**, 6-311G**, 6-311 + G*, 6-311G* in combination with B2PLYP. A comparison between the values in Table S25 reveals that generally, the alterations of the topological parameters are minor, except in the case of the C≡N bond. This finding is mirrored by the fluctuation of the magnitude and sign of ∇^2^ρ (r_c_). While the values of the Laplacian for the C≡N bond are negative, as suggested by the triple zeta basis sets 6-311 + G**, 6-311G**, 6-311 + G*, and 6-311G*, these values converted to positive when the double zeta basis sets 6-31 + G* and 6-31G* were applied. Such a sign inversion of the Laplacian means, however, that regardless of the extension of the basis set by simply employing a double zeta instead of triple zeta basis set, the C≡N bond character changes from a covalent to an ionic type, which is at least questionable and even absurd. This conspicuous dependency of the topological properties of the C≡N bond on the basis set confirms the results that we previously discussed [90] but contradicts the conclusion that was drawn earlier by Aray et al. [91], asserting that the topological properties of the C≡N and the R-CN bonds are minimally affected by the variation of the basis set. Accordingly, further investigations should be conducted to explore whether other electron-rich bonds in different molecular surroundings show similar striking behavior and dependency on the quality of the basis set.

Now, a few comments on the effect of fluorination of silaspirocyclic imines in terms of the AIM quantum theory. As an example, OITSSHC-33 was chosen and substituted by fluorine at various positions by four, eight, and twelve fluorine atoms. Table S26 visualizes some of the results, which were obtained from the AIM analysis. For reasons of brevity, only some selected examples of the peculiarities engendered by the fluorine substitution will be discussed.

As can be seen in Table S26, the noticeably large negative values of the Laplacian ∇^2^ρ(r_c_) (a high concentration of electron density at the bond critical points) associated with the N_1_=C_2_ bond in the *term*-F4- and *cent*-F4- derivatives (Figure 4) emphasize the accentuated covalent character of this bond. This covalency of the -N=C bond weakens upon the addition of fluorine substituents on C_2,4_ (and by symmetry on C_14_,_16_) in F8- and F12- derivatives, which is substantiated by the noticeably low ∇^2^ρ(r_c_) value of −0.103 e/$a_{0}^{5}$ (Table S26). Moreover, the apparent growth of the ellipticity value in F8- and F12- derivatives (0.195 and 0.205, respectively) in comparison to those in *term*-F4- and *cent*-F4- clearly reveals the increase in the π-character in the former fluorinated OITSSHC-33 than in the latter F4-derivatives.

Obviously, the strong charge withdrawal by the electronegative fluorine and the subsequent reduction in the π-character of the -N=C double bond is well-indicated by the alteration of the values of the Laplacian density and the ellipticity at the bond critical points.

Table S26 also shows that the C-F bonds in *cent*-F4-, F8-, and F12- derivatives exhibit a covalent (or slightly polar) character, which is specified by the small negative values of ∇^2^ρ(r_c_).

Further interesting features of this topological analysis are related to the Si-F bond in the terminally fluorinated derivatives of OITSSHC-33, i.e., *term-*F4- and F12- in Table S26. Strikingly, at the *bcp* of this bond, the charge density concentration is considerably low (0.11 E^−^), and the positive value of the ∇^2^ρ(r_c_) is large (charge density is locally depleted at the *bcp*), and the position of the *bcp* along the Si-F bond path (closer to the evidently less electronegative silicon) confirms the accentuated ionicity (the weakness) of this bond and, therefore, its higher reactivity. Moreover, the values of the bond ellipticity are noticeably small, indicating the proximity of this bond to the cylinder symmetry at the *bcp*. It remains to be mentioned that a comparison between the topological properties (in terms of the QTAIM) of the Si-F and C-F bonds in these fluorinated derivatives reveals that these two bonds are evidently different in nature. In contrast to the C-F bond, the Si-F bond is ionic or strong polar, according to the evidently large positive values of ∇^2^ρ(r_c_) at the *bcp* (0.99 e/$a_{0}^{5}$ in *term*-F4- and 1.07 e/$a_{0}^{5}$ in F12-) and the position of the *bcp* along the bond path (the *r_A_* and *r_B_* values show that the *bcp* is closer to the positively charged silicon atom). All these considerations affirm that the Si-F bond is clearly weaker than the C-F bond.

Furthermore, to probe the influence of the atoms surrounding the spiro centers in SCIs, the topological properties of ρ(r_c_) in TISS***N***U-19 and TISS***C***U-19 (with N-Si-N and C-N-C spiro centers, respectively), as well as their tetracyano derivatives, have been analyzed. From the obtained results, which are shown in Table S24, is apparent that the variation of the environment of the spiro center has a perceptible effect, predominantly on ∇^2^ρ (r_c_) distribution around the spiro center. For example, markedly larger positive values (0.60 e/$a_{0}^{5}$) of the Laplacian of the charge density have been predicted for the N_1_-Si_6_ and Si_6_-N_7_ bonds in TISS***N***U-19 and TCTISS***N***U-23, indicating the occurrence of a closed shell interaction between nitrogen and silicon in the =N-Si bond and, thus, manifesting its accentuated ionicity. In contrast, the considerably smaller values of the Laplacian of the charge density ρ(r_c_) at the *bcps* of the C_1_-Si_6_ and Si_6_-C_7_ bonds of 0.28 e/$a_{0}^{5}$ in TISS***C***H-19 and TCTISS***C***U-23 show the lower polarity of these bonds and, therefore, the lower nucleophilicity of the C-Si-C spiro center in comparison to the N-Si-N spiro center. This finding demonstrates, from the perspective of the QTAIM, the ability for manipulating the nucleophilic character of the spiro center by an alteration of the surrounding atoms.

## 10. Spiro-Aromaticity

Numerous concepts of aromaticity (π- and σ-) and its criteria have been postulated and applied since Hückel’s pioneering work and the establishment of the (4n + 2) π-electron stability rule 1931 [92,93]. Without entering the jungle of these concepts, the main emphasis will be placed on the spiro-aromaticity based on the Heilbronner–Möbius type [94] and the Craig–Möbius type [95,96] because, as will be shown below, these models provide the most reasonable presuppositions to be invoked for the elucidations of the aromaticity in the spirocyclic imines presented in this work.

To get a sense of the role of the ring current and the aromatic/antiaromatic character of the investigated spiro-conjugated sila- and germa-spirocyclic imines, GIAO-NMR calculations were carried out to determine the aromaticity descriptors NICS (nuclear independent chemical shift) and its corresponding out-of-plane zz-component of the shielding tensor; NICSzz values utilizing the related routines are available in Gaussian 16 [25].

These aromaticity descriptors were introduced for the first time by Schleyer et al. [97,98], and NICS was defined as the negative value of the isotropic shielding tensor computed at a ring center or some other crucial points of a molecular system, and NICS*zz* was introduced to assess the out-of-plane component of the NICS shielding tensor account for the p-electron delocalization. It should be noted that both shielding tensor components, the in-plane NICS component as well as the out-of-plane component NICS_zz_, incorporate *σ*- and *π*-contributions; however, the latter component NICS_zz_ encompasses predominantly *π*-contribution. Additionally, the NICS(1) index, which defines the NICS value at 1 Å, above the ring plane, was proposed to avoid to a wide extent contamination by σ-aromaticity.

Despite the widespread use of the NICS index, some justified criticism has been directed against this aromaticity descriptor by Lazzeretti and many others [99,100,101].

Furthermore, new concepts for assessing aromaticity in molecular entities have been suggested, e.g., D2BIA (the density and delocalization-based index of aromaticity) and D3BIA (the density, degeneracy, and delocalization-based index of aromaticity) [102]. Nonetheless, the NICS aromaticity index has proven to be an essential and easily attainable quantity for the prediction of the aromatic (diatropic ring current) and antiaromatic (paratropic ring current) character of cyclic molecular systems.

**Figure 11:**
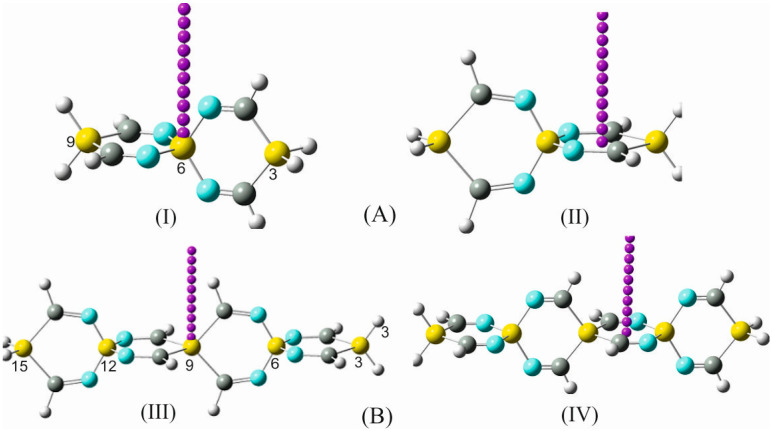
(**A**) Position of the Bq atoms in TISSNU-19: (**I**) above the spiro center Si6, (**II**) perpendicular to the center of a ring plane. (**B**) Position of the Bq atoms in OITSSHC-33: (**III**) above the central spiro silicon Si9, (**IV**)perpendicular to the center of a ring plane.

The NICS and NICS_zz_ values for the following selected representatives of SCIs were calculated: TISS***N***U-19, TSISS***N***U-19, OITSSHC-33, OSITSSHC-33, and OGITSSHC-33 (Figure 11 and Figure S18). Table 14 and Table 15, Tables S27 and S28 summarize the collected results and visualize the dependency of the NICS and NICS_zz_ values on the distance of the ghost (*Bq)* atoms from various reference positions in the spirocyclic scaffold.

The *Bq* arrays were positioned (a) above the silicon spiro center Si_6_ in TISS***N***U-19 and TSISS***N***U-19 (ranging from 0.5 Å to 5.0 Å with an increment of 0.5 Å). Such an arrangement was chosen despite possible magnetic contamination by interaction with some close ring atoms. The purpose of setting the *Bq* ghost atoms above the spiro center is to assess to that extent the spiro-aromaticity (charge delocalization and tunneling of electrons from one ring to the other) via the participation of the spiro center is “detectable” by means of the magnetic shielding (NICS) tensor, which is (b) perpendicular to the geometric center of the spiro ring plane and (c) perpendicular to the ring plane but closer to the spiro center Si_6_ by arbitrarily chosen 1.60 Å away from Si_6_ in TSISS***N***U-19 and 1.50 Å in TISS***N***U-19 (for (b) and (c), the range for the *Bq* arrays is 0.0 Å to 5.0 Å with an increment of 0.5 Å). This strategy was adopted in order to investigate the alteration of the magnitude of the NICS shielding tensor upon the variation of the positions of the *Bq* patterns relative to the spiro center.

Before discussing the computed NICS values, it should be noted that prior to performing the NMR calculations, the geometries of the selected spirocyclic imines were optimized by employing the B3PLYP/aug-cc-pVDZ computational method.

Inspection of the displayed NICS values in Table 14 and the NICS_zz_ values in Table 15 leads to the following conclusions. (1) From the notedly negative isotropic (NICS)_iso_ values (−4.24 to −3.10 ppm) in the range between 0.5 Å and 2.5Å (with a maximum at 2.0 Å of −6.61 ppm) above the spiro center, Si_6_ indicates the aromatic character of the spirocyclic silaimine TSISS***N***U-19 (with a skeleton consisting of exclusively silicon and nitrogen atoms). Between 3.0 Å and 5.0 Å, the diatropicity (aromaticity) decays rapidly to zero. Similar behaviors of the (NICS)_iso_ values above the Si_6_ in TISS***N***U-19 are observed but with seemingly lower NICS values, except for the two values at 1.0 Å and 1.5 Å, which become positive (deshielded), simulating a paratropic (anti-aromatic) behavior. However, these “outliers” are most likely produced due to the proximity of the ghost atoms to the imino nitrogen in this region and, thus, magnetic contamination (Figure 11).

Conspicuously, the negative NICS(0.5) value above Si_6_ in TSISS***N***U-19 is 1.17 ppm larger than the NICS(1) value. (2) As is also apparent in Table 14, the isotropic NICS values above the ring plane and closer to the spiro center Si_6_ ununiformly start in both TSISS***N***U-19 and TISS***N***U-19, with positive NICS)_iso_ values indicating anti-aromatic character, which then change to negative and small values (<1 ppm), signifying very weak diamagnetic aromatic character (nonaromatic). (3) The out-of-plane zz-components of the shielding tensor NICS_zz_ values in Table 15 show generally similar tendency, like (NICS)_iso_, upon moving to larger distances above the spiro center Si_6_ and equally above the ring geometric center, as well as above the reference position of the *Bq* atoms closer to the spiro center. In analogy to the (NICS)_iso_ values, the NICS_zz_ values at distances between 0.5 and 5.0 Å above the spiro center Si_6_ possess explicitly negative values in the range of −4.30 to −1.3 ppm for TSISS***N***U-19 and −13.30 to −2.55 ppm for TISS***N***U-19 at 0.5–2.5 Å, with a maximum NICS_zz_ value of −10.03 ppm for the former and −9.38 ppm for the latter at 1.5 Å. Such variations of the NICS_zz_ and (NICS)_iso_ values associated with the *Bq* atoms above the spiro center clearly visualize the aromaticity of these smallest representatives of the spirocyclic imines presented in this investigation.

To explore whether the enlargement of the spirocyclic imine framework has any effect on the aromatic behavior and the delocalization of electrons in larger spiro systems, the magnetic shielding tensors for the following SCIs were computed: (**I**) OITSSHC-33, (**II**) the analogs cyclic sila-imine OSITSSHC-33 (consisting exclusively of silicon, nitrogen, and hydrogen atoms), and (**III**) the cyclic germaimine OGITGSHC-33 (comprising solely germanium, nitrogen, and hydrogen atoms) (Tables S27 and S28). In all these spirocyclic imines, the ghost *Bq* atoms were positioned (a) above the spiro center Si_9_/Ge_9_ and (b) perpendicular to the geometric center of a ring plane neighboring the spiro center Si_9_/Ge_9_ (Figure 11 and Figure S18). A comparison between the calculated NICS values (Table S27) for these larger spirocyclic imines reveals (i) the explicitly negative NICS(0.5) and NICS(1.0) values at the position (a) in (**I**), (**II**), and (**III**), respectively, signalizing an appreciable diatropic ring current and, thus, confirming the apparent aromaticity of these spiro units with respect to the *Bq* ghost atoms positioned above the silicon/germanium spiro center Si_9/_Ge_9_. However, it is likely that the NICS(0.5) values are, to some extent, adulterated due to the propinquity of the *Bq*(0.5) probe to the spiro center Si_6_/Ge_5_. (ii) The largest NICS values are associated with the cyclic germaimine OGITSSHC-33. (iii) Notably, the negative large isotropic NICS values above the spiro center Si_9_/Ge_9_ change abruptly to positive values (they are different for the listed spirocyclic imines in Table S27) at 1.5 and 2.0 Å in (I), at 2.0 and 2.5 Å in (**II**), and at 2.5 Å in (**III**). One conceivable explanation for this sudden reversal of the sign of the NICS values is the distortion of the magnetic shielding provoked by the proximity of the *Bq* atoms in this region (between 1.5 and 2.5 Å) to the ring atoms (*Bq* atoms and adjacent ring atoms are in the same plane, and the distances between Bq(1.5) and ring carbon is 1.1 Å and between *Bq*(2.5) and the ring hydrogen atom in the Si-C(H) = N fragment is 0.7 Å) (see Figure 11 and Figure S18). (iv) Thereafter, the negative NICS values decline successively, reaching zero at NICS(5.0). (v) Remarkably, in all three compounds shown in Table S27, the NICS values above the ring geometric center are either positive (deshielded), denoting paratropic ring currents and anti-aromatic behavior, or small and negative, indicating weak aromatic character. (vi) The out-of-plane tensor components NICS_zz_ in Table S28 exhibit uniformly for all SCI (at the (a) and (b) positions of the *Bq* arrays) negative values at *Bq*(1.0), demonstrating diatropic shielding (aromatic character). However, these values are clearly more accentuated for the sila- and germaimino compounds OSITSSHC-33 and OGITSSHC-33 (NICS_zz_(1.0) is −18.2 ppm for the former and −6.2 ppm for the latter). (vii) Except for OSITSSHC-33 at position (a), all remaining NICS_zz_ values between *Bq*(1.5) and *Bq*(5.0) (for all three SCIs in Table S28) are positive, denoting a paratropic (antiaromatic) behavior.

In summary, from all indications of the aromatic character exhibited by SCIs as provided by the aromaticity descriptor NICs and NICS_zz_, it can be postulated that a spirocyclic imine framework consisting of a certain number of rings resembles a large, conjugated ring with a half-twist. Intrinsically, this class of spiro compounds (formally half-twisted and cyclically conjugated) fulfill the criteria of the Möbius 4nπ aromaticity and, thus, belong to the family of spiro-aromatic compounds. However, based on the peculiarity of the addressed spirocyclic imines consisting of silicon or germanium spiro centers, each of which is alternatively surrounded by four -N=C/Si/Ge bonds or four -C/Si/Ge=N bonds, this distinct type of spiro-aromaticity can be more specifically designated as “SCI aromaticity”. It is of interest to note that the term “spiro aromaticity” was first used by Harada et al. [103]. Both concepts of the Möbius-type aromaticity, the Heilbronner p_π_-p_π_ or Craig p_π_-d_π_, can be invoked to rationalize the spiro-aromaticity of the variety of the studied SCIs. Depending on whether silicon or germanium is occupying the spiro center, this kind of aromaticity can be classified either as Heilbronner–Möbius type aromaticity [94] in the former case (silicon spiro center) or Craig–Möbius type aromaticity [96] in the case of germanium because of the involvement of 3D orbitals (2p_π_ orbitals on nitrogen and 3d_π_ on Ge). Rzepa and his research group [104,105] have studied some spiro compounds and discussed the role of spiro-aromaticity by affecting their chemical properties and stability. Undoubtedly, more research has yet to be accomplished in order to establish this type of spirocyclic imine aromaticity (SCI aromaticity) more comprehensively and unequivocally as a robust model of stabilizing aromaticity notion. For this purpose, additional spirocyclic compounds incorporating different transition metals of the group of four elements as a spiro center, e.g., Ti, Zr surrounded by four N=Y bonds (where Y=P or a group 13 element) will be investigated.

Finally, all SCIs and their derivatives have been theoretically studied, and the results presented and discussed in this work represent an inspiration and appealing challenge for synthetic chemists to synthesize these novel sila- and germa-spirocyclic imines and make them accessible for general applications. They include multifunctional materials, photonics, optoelectronics, metal-organic semiconductors, functional coatings, and drug discoveries.

The dual nature of sila SCIs (and their isosteric germa SCIs) as simultaneous nucleophiles and electrophiles and their three-dimensionality and intra-molecular charge mobility aromaticity impart their peculiar chemical reactivity and biological activity.

Furthermore, the specific combination of unique structural and electronic features in SCIs (and their above-discussed derivatives) plays an essential role by the selective interactions with other chemical or biological (e.g., as a pharmacophore or attached to an active pharmaceutical ingredient (API)) entities. It can be anticipated that the imino group embedded in SCIs or their bioisosteres would unfold a wide scope of crucial biologically active properties, which can be utilized in medicinal and pharmaceutical chemistry.

## 11. Conclusions

The uniqueness of this novel class of cyclic imines is mirrored by their three-dimensionality, the simultaneous occurrence of internal nucleophilic electron donors (imino groups = Lewis base), and electrophilic charge acceptors (the silicon/germanium spiro centers = Lewis acid). The cross-hyperconjugative interactions that provoke electrons to shuttle back and forth (charge mobility) along the spiro-cyclic framework, inducing a special kind of aromaticity, which is specified as “spiro aromaticity”, has been clearly shown and discussed. All these distinctive characterizations of the sila-/germa-spirocyclic imines testify to their versatility to be utilized in a diversity of advanced materials. For a reasonable interpretation and substantiation of all the collected results and features related to the discussed novel class of SCIs in this work, various complementary quantum chemical concepts have been utilized. Finally, it is worth emphasizing that all the obtained structural results refer to single molecular entities in the gas phase, and accordingly, no inter-molecular interactions are considered.

## Figures and Tables

**Table 1:** Some structural parameters of (**I**) TISS***N***U-19, (**II**) HIDSS***NC***H-26 and (**III**) OITSSHC-33, and (**IV**) DITSSHC-40 (for these abbreviations, see the text and Figure 1). B2PLYP/aug-cc-pVDZ was used. Bond lengths are in Å and bond angles are in degrees.

Bond Lengths	**(I)**	**(II)**	**(III)**	**(IV)**	Bond Angles	**(I)**	**(II)**	**(III)**	**(IV)**
N_1_=C_2_	1.288	1.288	1.288	1.288	∠N_1_=C_2_-Si_3_	125.3	125.3	125.4	125.4
Si_3_-C_2_=	1.920	1.921	1.921	1.921	∠=C_2_-Si_3_-C_4_=	106.6	106.6	106.6	106.6
Si_6_-N_1_=	1.770	1.769	1.769	1.768	∠Si_6_-N_1_=C_2_	122.9	122.8	122.8	122.7
Si_6_-N_7_=		1.770	1.769	1.771	∠=N_1_-Si_6_-N_5_=	116.9	117.2	117.1	117.2
=N_11,13_-Si_12_		1.785		1.769	∠Si_6_-N_7_=C_8_		123.2	123.2	123.3
=N_17,19_-Si_18_				1.786	∠=N_7_-Si_6_-N_16(21)(26)_		116.4	116.5	116.4
Si_3_-H	1.492	1.492	1.492	1.492	∠=N_11_-Si_12_-N_13(23)_		115.2		116.7
Si_12_-H		1.483			∠Si_15_-C_16_=N_17_				125.4
Si_18_-H				1.483	∠Si_18_-N_17_=C_16_				123.8
					∠=N_17_-Si_18_-N_19_				115.2
					∠H-Si_3_-H	108.7	108.8	108.8	108.9
					∠H-Si_12((18)_-H		111.7		111.8

**Table 2:** Comparison between some structural parameters of (**I**) TISS***N***U-19 and (**II**) OITSSHC-33, as predicted by (***A***) B2PLYP/aug-cc-pVDZ, and (***B***) MP2-aug-cc-pVDZ levels of theory (for these abbreviations, see the text and Figure 1). Bond lengths are in Å and bond angles are in degrees.

	**(*A*)**		**(*B*)**			**(*A*)**		**(*B*)**	
Bond Lengths	**(I)**	**(II)**	**(I)**	**(II)**	Bond Angles	**(I)**	**(II)**	**(I)**	**(II)**
N_1,5_=C_2,4_	1.288	1.288	1.301	1.301	∠N_1,5_=C_2,4_-Si_3_	125.3	125.3	125.6	125.6
Si_3_-C_2,4_=	1.920	1.921	1.922	1.923	∠=C_2_-Si_3_-C_4_=	106.6	106.6	106.9	106.9
Si_6_-N_1,5_=	1.770	1.769	1.776	1.775	∠Si_6_-N_1,5_=C_2,4_	122.9	122.8	122.0	121.7
Si_6_-N_7,11≡_		1.770		1.778	∠=N_1_-Si_6_-N_5_=	116.9	117.1	118.0	118.3
N_7,11_=C_8,10_		1.287		1.301	∠Si_6_-N_7,11_=C_8,10_		123.2		122.0
Si_9_-C_8,10_=		1.921		1.920	∠=N_7_-Si_6_-N_11_=		116.5		117.9
					∠Si_9_-C_8,10_=N_7,11_		125.4		125.6
					∠=C_8_-Si_9_-C_10_=		106.4		107.0

**Table 3:** Some structural parameters of (**I**) TIGS***N***U-19, (**II**) HIDGS***NC***H-26, and (**III**) OITGSHC-33 (for these abbreviations, see the text and Figure S1). B2PLYP/aug-cc-pVDZ was used. Bond lengths are in Å and bond angles are in degrees.

Bond Lengths	**(I)**	**(II)**	**(III)**	Bond Angles	**(I)**	**(II)**	**(III)**
N_1,5_ = C_2,4_	1.285	1.285	1.285	∠N_1_=C_2_-Ge_3_	127.0	127.1	127.1
Ge_3_-C_2,4_=	1.993	1.994	1.994	∠=C_2_-Ge_3_-C_4_=	106.4	106.4	106.4
Ge_6_-N_1,5_=	1.876	1.874	1.874	∠Ge_6_-N_1_=C_2_	121.2	121.1	121.0
Ge_6_-N_7_=		1.875	1.875	∠=N_1_-Ge_6_-N_5_=	117.2	117.4	117.4
=N_11,13_-Ge_12_		1.890		∠=N_7_-Ge_6_-N_16(21)_=		116.8	116.8
Ge_3_-H	1.539	1.539	1.539	∠C_10_-N_11_ = Ge_12_		122.7	
Ge_12_-H		1.531		∠=N_11_-Ge_12_-N_13_=		114.9	
				∠H-Ge_3_-H	109.2	109.3	109.3
				∠H-Ge_12_-H		113.1	

**Table 4:** Some structural parameters of spirocyclic-silaimines: (**I**) TSISS***N***U-19, (**II**) HSIDSS***NC***H-26, and (**III**) OSITSSHC-33 (for these abbreviations, see the text and Figure 2 and Figure S3). B2LYP/aug-cc-pVDZ was used. Bond lengths are in Å and bond angles are in degrees.

Bond Lengths	**(I)**	**(II)**	**(III)**	Bond Angles	**(I)**	**(II)**	**(III)**
N_1_=Si_2_	1.629	1.629	1.629	∠N_1_=Si_2_-Si_3_	121.2	121.1	121.1
=Si_2_-Si_3_	2.365	2.366	2.365	∠=Si_2_-Si_3_-Si_4_=	98.4	98.5	98.4
Si_6_-N_1_=	1.759	1.755	1.757	∠Si_6_-N_1_=Si_2_	129.8	129.7	129.8
Si_6_-N_7_=		1.762	1.760	∠=N_1_-Si_6_-N_5_=	119.5	119.9	119.6
N_7_=Si_8_		1.627	1.628	∠Si_6_-N_7_=Si_8_		130.1	129.8
Si_9_-Si_8_=		2.365	2.362	∠=N_7_-Si_6_-N_16(21)_		118.6	118.9
Si_9_-Si_10_		2.363		∠Si_9_-Si_8_=N_7_		121.9	122.0
Si_10_=N_11_		1.630		∠=Si_8_-Si_9_-Si_15(20)_		97.6	97.6
=N_11_-Si_12_		1.765		∠=Si_10_-Si_9_-Si_14_=		97.4	
Si_3_-H	1.495	1.494	1.494	∠Si_9_-Si_10_=N_11_		122.2	
Si_12_-H		1.490		∠=N_11_-Si_12_-N_13_=		118.5	
				∠H-Si_3_-H	108.4	108.6	108.5
				∠H-Si_12_-H		110.1	

**Table 5:** Selected structural parameters of tetracyano derivatives of some SSCIs: (**I**) TCTISS***N***U-23 with N-Si-N spiro center), (**II**) TCHIDSS***NC***H-30 (with N-Si-N and C-Si-C spiro centers), (**III**) TCOITSSHC-37*,* and (**IV**) TCDITSSHC-44 (with alternating N-Si-N and C-Si-C spiro centers). For abbreviations, see the text and Figure S5). B2PLYP/aug-cc-pVDZ was used. Bond lengths are in Å and bond angles are in degrees.

Bond Lengths	**(I)**	**(II)**	**(III)**	**(IV)**	Bond Angles	**(I)**	**(II)**	**(III)**	**(IV)**
N_1,5_=C_2,4_	1.284	1.284	1.283	1.283	∠N_1_=C_2_-Si_3_	123.6	123.5	123.4	123.6
Si_3_-C_2,4_=	1.915	1.915	1.914	1.915	∠=C_2_-Si_3_-C_4_=	108.4	108.5	108.6	108.4
Si_6_-N_1_=	1.768	1.770	1.772	1.772	∠Si_6_-N_1_=C_2_	123.8	124.1	124.3	124.2
Si_6_-N_7_		1.765	1.764	1.764	∠=N_1_-Si_6_-N_5_=	116.7	116.2	116.0	116.0
N_7_=C_8_		1.287	1.288	1.288	∠Si_6_-N_7_=C_8_		123.2	122.8	122.6
Si_9_-C_8_=		1.923	1.923	1.923	∠=N_7_-Si_6_-N_16(21)(26)_=		116.9	117.3	117.5
=N_11,13_-Si_12_		1.762		1.767	∠=N_11_-Si_12_-N_13,(23)_=		118.1		117.1
=N_17,19_- Si_18_				1.762	∠Si_15_-C_16_=N_17_				126.1
Si_3_-C≡	1.855	1.856	1.857	1.858	∠Si_18_-N_17_=C_16_				121.9
Si_12(18)_-C≡		1.853		1.853	∠=N_17_-Si_18_-N_19_				118.3
C≡N	1.174	1.174	1.174	1.174	∠≡C-Si_3_-C≡	106.5		106.2	106.3
					∠≡C-Si_12,18_-C≡		108.3		108.2

**Table 6:** A comparison between the structural parameters of (**I**) 1,4-disila-3,5-diimino-cyclohecane (DSDICH-12), (**II**)1,1,4,4-F_4_-DSDICH-12, (**III**) 3,5-F_2_-DSDICH-12, (**IV**) 1,1,3,5,4,4-F_6_-DSDICH-12, (**V**) TISS***N***U-19, (**VI**) 3,3,9,9-F_4_-TISS***N***U-19, (**VII**) 2,4,8,10-F_4_-TISS***N***U-19, and (**VIII**) 3,3,9,9,2,4,8,10-F_8_-TISS***N***U-19 as predicted B2PLYP/aug-cc-pVD levels of theory (for these abbreviations, see the text and Figure 4). Bond lengths are in Å and bond angles are in degrees.

Bond Lengths	**(I)**	**(II)**	**(III)**	**(IV)**	Bond Angles	**(I)**	**(II)**	**(III)**	**(IV)**
N_2_=C_3_	1.288	1.290	1.255	1.259	∠N_2_=C_3_-Si_4_	125.4	123.7	130.0	127.6
=C_3_-Si_4_	1.921	1.899	1.916	1.903	∠=C_3_-Si_4_-C_5_=	106.7	109.3	100.8	103.8
Si_1_-N_2_=	1.785	1.761	1.780	1.750	∠Si_1_-N_2_ = C_3_	123.5	122.5	123.0	122.4
Si_4_-F		1.633		1.619	∠=N_2_-Si_1_-N_6_=	115.7	118.3	113.2	116.2
Si_1_-F		1.618		1.613	∠=F-Si_1_-F		108.2		108.7
C_3_-F			1.388	1.376	∠=F-Si_4_-F		106.1		108.8
Bond Lengths	**(V)**	**(VI)**	**(VII)**	**(VIII)**	Bond Angles	**(V)**	**(VI)**	**(VII)**	**(VIII)**
N_1_=C_2_	1.288	1.289	1.257	1.258	∠N_1_=C_2_-Si_3_	125.3	123.4	129.9	127.4
=C_2_-Si_3_	1.920	1.896	1.919	1.901	∠=C_2_-Si_3_-C_4_=	106.6	109.8	100.8	104.1
Si_6_-N_1_=	1.770	1.771	1.758	1.757	∠Si_6_-N_1_ = C_2_	122.9	123.1	122.2	122.8
Si_3_-F		1.635		1.619	∠=N_1_-Si_6_-N_5_=	116.9	117.3	115.1	115.5
C_2_-F			1.381	1.367	∠=F-Si_3_-F		105.6		108.6

**Table 7:** Natural charges *q*_NPA_ in sila- and germa-spirocyclic imines: (**I**) TISS*N*U-19, (**II**) TCSSTI*N*U-23, (**III**) OITSSHC-33, (**IV**) TCOITSSHC-37, (**V**) TIGST*N*U-19, and (**VI**) TCGSTI*N*U-23. The B2PLYP/aug-cc-pVDZ method was used. For abbreviations, see the text and Figure 1 and Figure S5.

Atom	**(I)**	**(II)**	Atom	**(III)**	**(IV)**	Atom	**(V)**	**(VI)**
N_1,5_	−0.893	−0.862	N_1,5_	−0.896	−0.857	N_1,5_	−0.848	−0.817
C_2,4_	−0.226	−0.223	C_2,4_	−0.223	−0.230	C_2,4_	−0.201	−0.194
Si_3,9_	1.100	1.476	Si_3,15_	1.099	1.475	Ge_3,9_	0.972	1.372
S_i6_	2.262	2.259	S_i6,12_	2.261	2.260	Ge_6_	2.120	2.123
H_(C)_	0.184	0.209	N_7_	−0.881	−0.885	H_(C)_	0.183	0.211
H_(Si)_	−0.179		C_8,10_	−0.224	−0.235	H_(Si)_	−0.150	
C≡		−0.174	Si_9_	1.459	1.456	C≡		−0.141
≡N		−0.254	H_(C)_	0.192	0.206	≡N		−0.276
			H_(Si)_	−0.178				
			C≡		−0.170			
			≡N		−0.260			

**Table 8:** Mulliken atomic charges *q_M_* in some silaspirocyclic imines and their tetracyano derivatives: (**I**) TISS***N***U-19, (**II**) TCTISS***N***U-23, (**III**) OITSSHC-33, (**IV**) TCOITSSHC-37. The B2PLYP/aug-cc-pVDZ computational method was used. For abbreviations, see the text and Figure 1 and Figure S5.

Atom	**(I)**	**(II)**	Atom	**(III)**	**(IV)**
N_1,5_	−0.541	−0.526	N_1,5_	−0.475	−0.535
C_2,4_	0.334	0.333	C_2,4_	0.284	0.306
Si_3,9_	−0.185	1.351	Si_3_	−0.204	1.265
S_i6_	2.119	2.361	S_i6,12_	2.493	2.538
H_(C)_	−0.544	−0.564	N_7_	−0.622	−0.498
H_(Si)_	0.314		C_8_	0.356	0.326
C≡		−0.127	Si_9_	0.915	0.987
≡N		−0.380	H_(C)_	−0.682	−0.691
			H_(Si)_	0.326	
			C≡		−0.090
			≡N		−0.390

**Table 9:** Some NBO donor–acceptor interactions and stabilization energies, E(2), in various sila- and germa-spirocyclic imines as estimated by the second-order perturbative scheme. (**I**) TISS*N*U-19, (**II**) TCTISS*N*U-23, (**III**) TIGS*N*U-19, (**IV**) TCTIGS*N*U-23. B3LYP/cc-pVDZ was applied. E2 is in kcal∙mol^−1^. For atomic numbering, see the text and Figure 1, Figures S1 and S5.

Donor Orbital	Acceptor Orbital	E(2)	Donor Orbital	Acceptor Orbital	E(2)
**(I)**			**(III)**		
π _(2)N1=C2_	σ*_Si6-N7,11=_	3.1	π _(2)N1=C2_	σ*_Ge6-N7,1=_	2.6
σ_Si6-N1=_	σ*_Si6-N5=_	3.6	σ_Ge6-N1=_	σ*_Ge6-N5=_	6.6
σ_Si6-N7=_	σ*_Si6-N11=_	3.6	σ_Ge6-N1=_	σ*_Ge6-N7,11=_	4.0
σ_C2-Si3_	σ*_Si3-C4=_	2.1	σ_C2-Ge3_	σ*_Ge3-C4=_	3.4
(LP)_N1_	σ*_Si3-C2=_	13.2	σ_Ge6-N7=_	σ*_Ge6-N11=_	6.6
(LP)_N1_	σ*_Si6-N5=_	7.5	(LP)_N1_	σ*_Ge3-C2=_	14.1
(LP)_N1_	σ*_C2-H_	4.6	(LP)_N1_	σ*_Ge6-N5=_	6.0
			(LP)_N1_	σ*_C2-H_	3.0
**(II)**			**(IV)**		
π _(2)N1-C2_	σ*_Si6-N7,11=_	3.0	π _(2)N1-C2_	σ*_Ge6-N7,11=_	2.5
σ_Si6-N1_	σ*_Si6-N5=_	3.9	σ_Ge6-N1_	σ*_Ge6-N5=_	6.9
σ_Si3-C≡_	σ*_C≡N_	3.5	σ_Ge3-C≡_	σ*_C≡N_	2.2
π _(2)N7-C8_	σ*_Si6-N1,5=_	3.0	π _(2)N7-C8_	σ*_Ge6-N1,5=_	2.5
(LP)_N1_	σ*_Si3-C2=_	13.3	(LP)_N1_	σ*_Ge3-C2=_	14.5
(LP)_N1_	σ*_Si6-N5=_	7.3	(LP)_N1_	σ*_Ge6-N5=_	5.9
(LP)_N1_	σ*_C2-H_	4.4	(LP)_N1_	σ*_C2-H_	2.8
(LP)_N≡_	σ*_Si- C≡_	8.6	(LP)_N≡_	σ*_Ge-C≡_	9.2

**Table 10:** Some NBO donor–acceptor interactions and stabilization energies, E(2), in (**I**) OITSSHC-33 and its fluorine derivatives: (**II)** *cent*-F4 (four fluorine atoms positioned around the silaspiro center C-Si-C, (**III)** F8-OITSSHC-33 (fluorine atoms on all carbon atoms)*,* and (**IV**) perfluoro-derivative F12-OITSSHC-33 as estimated by the second-order perturbative scheme using B3LYP/aug-cc-pVDZ. E2 is in kcal∙mol^−1^. For abbreviations and atomic numbering, see the text and Figure 4.

Donor Orbital	Acceptor Orbital	**E**(**2**)			
		(**I**)	(**II**)	(**III**)	(**IV**)
π _N1-C2_	σ*_Si6-N7,21_	3.1	3.6	3.8	3.7
σ_Si6-N1,5_	σ*_C2,4-F_			14.6	14.0
σ_Si6-N1_	σ*_Si6-N5_	3.5	3.5	4.4	4.6
σ_Si6-N7_	σ*_Si6-N11_	3.7	4.9	4.9	4.7
σ_Si6-N7_	σ*_C8-F_		15.9	15.4	15.1
π _(2)N7-C8_	σ*_Si6-N1,5_	3.3	3.3	3.5	3.6
σ_C2-Si3_	σ*_Si3-C4_	2.6	2.6	3.4	4.1
σ_C2,4-Si3_	σ*_Si3-F_				3.9
(LP)_N1_	σ*_Si3-C2_	12.1	11.9	11.6	10.1
(LP)_N1_	σ*_Si6-N1_			4.1	3.8
(LP)N1	σ*_C2,4-F_			9.7	9.8
(LP)_N1_	σ*_Si6-N5_	8.5	8.7	7.3	7.3
(LP)^N7^	σ*_Si9-C8_	11.6	12.3	12.3	12.3
(LP)^N7^	σ*_Si6-N7_		5.2	5.0	4.7
(LP)_N7_	σ*_Si6-N21_		6.4	6.6	6.8
(LP)_N7,21_	σ*_C8,20-F_			9.1	9.1
(LP2)_F26_	σ*_N7-C8_		7.8	7.9	8.0
(LP2)_F23_	σ*_Si3-C2,4_				5.9
(LP3)_F23_	σ*_Si3-F24_				11.8

**Table 11:** Predicted HOMO-LUMO frontier molecular orbital energy gaps in OITSSHC-33 and its fluoro-derivatives (for abbreviations, see the text and Figure 7 and Figure S13). B2PLYP/aug-cc-pVDZ was applied. All values are given in eV.

	E_HOMO_	E_LUMO_	E_gap_		E_HOMO_	E_LUMO_	E_gap_
OITSSHC-33	−7.537	−0.871	6.67	TISS*N*U-19	−7.429	−0.299	7.13
*term*-F4-	−7.973	−1.279	6.69	TISS*C*U-19	−7.674	−0.871	6.80
*cent*-F4-	−7.918	−0.789	7.13	TIGS*N*U-19	−7.238	0.027	7.21
F8-	−9.578	−1.252	8.33	TCTISS*N*U-23	−8.762	−1.687	7.07
F12-	−10.014	−1.714	8.30	TCTISS*C*U-23	−9.116	−2.259	6.86
				TCTIGS*N*U-23	−8.626	−1.578	7.05

**Table 12:** Calculated HOMO-LUMO energy gaps and global reactivity descriptors for some substituted silaspiro-cyclic imines: η = global chemical hardness, µ = molecular chemical potential, χ = molecular electronegativity, ω = global electrophilicity index. The B3LYP/aug-cc-pVDZ was applied. All values are given in eV. For abbreviations, see the text and Figure 7, Figures S9, S13 and S17.

	E_HOMO_	E_LUMO_	E_gap_	η	µ	χ	ω
OITSSHC-33	−6.150	−2.476	3.674	1.837	−4.313	4.313	5.063
*term*-F4- ^(a)^	−6.558	−2.884	3.674	1.837	−4721	4.721	6.066
*cent*-F4- ^(a)^	−6.476	−2.422	4.054	2.017	−4.449	4.449	4.907
F8- ^(a)^	−8.055	−2.803	5.252	2.626	−5.429	5.429	5.612
F12- ^(a)^	−8.408	−3.265	5.143	2.572	−5.837	5.837	6.622
DODIPSSHT-47	−6.177	−2.667	3.510	1.755	−4.422	4.422	5.571
silole	−6.558	−1.768	4.789	2.395	−4.163	4.163	3.618
gelole	−6.530	−1.660	4.870	2.435	−4.095	4.095	3.443
TISS*N*U-19	−6.041	−1.986	4.055	2.027	−4.014	4.014	3.973
TISS*C*U-19	−6.259	−2.449	3.810	1.905	−4.354	4.354	4.976
TIGS*N*U-19	−5.932	−1.660	4.272	2.136	−3.796	3.796	3.373
TCTISS*N*U-23	−7.347	−3.347	4.000	2.000	−5.347	5.347	7.148
TCTISS*C*U-23	−7.619	−3.782	3.837	1.919	−5.700	5.700	8.465
TCTIGS*N*U-23	−7.320	−3.211	4.109	2.055	−5.266	5.266	6.748
3,9-diazirid- ^(b)^	−5.796	−1.850	3.946	1.973	−3.823	3.823	3.704
3,9-diazetid- ^(b)^	−5.469	−1.742	3.727	1.864	−3.606	3.606	3.488
3,9-tet-azirid- ^(b)^	−5.687	−1.905	3.782	1.891	−3.796	3.796	3.810
3,9-tet-azetid- ^(b)^	−5.034	−1.442	3.592	1.796	−3.238	3.238	2.919
3,9-disilole-^(b)^	−6.068	−2.313	3.755	1.878	−4.191	4.191	4.677
3,9-tet-silole- ^(b)^	−5.959	−2.286	3.673	1.837	−4.123	4.123	4.628
3,9-tet-gelole- ^(b)^	−5.469	−1.660	3.809	1.905	−3.565	3.565	3.336
TITSSNDTE-31 ^(c)^	−6.041	−2.204	3.837	1.919	−4.123	4.123	4.430
TITGDNDTE-31 ^(c)^	−5.850	−1.932	3.918	1.959	−3.891	3.891	3.864
OITSS*C*ND-*D*-27 ^(c)^	−6.830	−3.048	3.782	1.891	−4.939	4.939	6.450
OITSS*C*ND-*V*-27 ^(c)^	−6.504	−2.748	3.756	1.878	−4.626	4.626	5.698

^(a)^ derivatives of OITSSHC-33. ^(b)^ Derivatives of TISS***N***U-19 (see Figure S9). ^(c)^ See Figure S11.

**Table 13:** Atomic and critical point properties of some bonds in the silaspirocyclic imine OITSSHC-33 and some of their substituted (with C≡N and fluorine) derivatives (Figure 1 and Figure 4, and Figure S5). For abbreviations, see the text. All quantities are in atomic units. The B2PLYP/aug-cc-pVDZ method.

	r_A_	r_B_	ρ(r_c_)	λ_1_	λ_2_	λ_3_	∇^2^ρ(r_c_)	ε_c_
OITSSHC-33								
N_1_=C_2_	1.598	0.836	0.365	−0.775	−0.723	0.589	−0.909	0.072
C_2_-Si_3_	2.256	1.373	0.107	−0.138	−0.133	0.555	0.284	0.034
N_1_-Si_6_	2.027	1.319	0.115	−0.180	−0.170	0.950	0.599	0.058
Si_6_-N_7_	1.320	2.029	0.114	−0.179	−0.169	0.946	0.598	0.060
C-H	1.300	0.708	0.273	−0.693	−0.689	0.389	−0.993	0.006
Si-H	1.364	1.423	0.111	−0.152	−0.152	0.582	0.278	0.000
TCOITSSHC-37								
N_1_=C_2_	1.594	0.831	0.368	−0.788	−0.724	0.625	−0.887	0.088
C_2_-S-i_3_	2.248	1.370	0.112	−0.144	−0.142	0.546	0.260	0.020
N_1_-Si_6_	2.031	1.321	0.113	−0.178	−0.167	0.940	0.595	0.067
Si_6_-N_7_	1.316	2.020	0.116	−0.183	−0.173	0.966	0.610	0.059
Si-C≡	1.356	2.152	0.104	−0.141	−0.136	0.680	0.403	0.036
C≡N	0.743	1.475	0.455	−0.987	−0.984	2.230	0.259	0.003
F8- ^(a)^								
N_1_=C_2_	1.562	0.815	0.396	−0.956	−0.800	0.728	−0.103	0.195
C_2_-Si_3_	2.258	1.370	0.104	−0.139	−0.133	0.593	0.320	0.048
N_1_-Si_6_	2.002	1.314	0.115	−0.183	−0.174	0.994	0.637	0.055
Si_6_-N_7_	1.318	2.011	0.113	−0.179	−0.168	0.969	0.622	0.061
C_2,4_-F	0.912	1.692	0.238	−0.465	−0.398	0.668	−0.194	0.169
C_8,10_-F	0.909	1.688	0.241	−0.471	−0.414	0.685	−0.200	0.138
Si-H	1.357	1.409	0.115	−0.161	−0.160	0.602	0.281	0.007
F12- ^(a)^								
N_1_=C_2_	1.561	0.817	0.396	−0.954	−0.792	0.713	−0.103	0.205
C_2_-Si_3_	2.237	1.355	0.113	−0.159	−0.156	0.635	0.321	0.016
N_1_-Si_6_	2.007	1.316	0.114	−0.182	−0.172	0.984	0.631	0.059
Si_6_-N_7_	1.316	2.007	0.114	−0.181	−0.171	0.982	0.631	0.060
C_2,4_-F	0.899	1.686	0.244	−0.483	−0.421	0.714	−0.190	0.149
C_8,10_-F	0.904	1.687	0.242	−0.477	−0.419	0.698	−0.198	0.140
Si-F	1.280	1.780	0.115	−0.210	−0.206	1.484	1.068	0.017

^(a)^ Derivatives of OITSSHC-33.

**Table 14:** Dependency of the NICS values (ppm) on the distance *r_Bq_* (Å) of the ghost atom *Bq* in (**I**) TSISS***N***U-19 and (**II**) TISS***N***U-19. (a) The *Bq* ghost atoms positioned above the silicon spiro center Si_6_, (b) the *Bq* atom array is set perpendicular to the center of the spiro ring plane, (c) the *Bq* atom array is set perpendicular to the ring plane and closer to the spiro center (for more details, see the text and Figure 11 and Figure S18). The B3LYP/aug-cc-pVDZ method was used.

	(I)			(II)		
*Bq*	NICS ^a^	NICS ^b^	NICS ^c^	NICS ^a^	NICS ^b^	NICS ^c^
0.0		1.64	0.61		5.78	
0.5	−4.24	1.06	0.23	−4.99	4.09	3.16
1.0	−3.07	0.21	−0.19	3.35	1.70	1.37
1.5	−6.42	−0.15	−0.27	3.75	0.59	0.57
2.0	−6.61	−0.21	−0.28	−3.34	0.14	0.16
2.5	−3.07	−0.19	−0.25	−3.16	−0.08	−0.10
3.0	−0.92	−0.15	−0.19	−1.37	−0.17	−0.20
3.5	−0.20	−0.11	−0.13	−0.58	−0.18	−0.21
4.0	−0.03	−0.08	−0.09	−0.32	−0.17	−0.18
4.5	0.00	−0.06	−0.06	−0.22	−0.14	−0.16
5.0	0.00	−0.04	−0.04	−0.16	−0.12	−0.13

**Table 15:** Dependency of the NICS_zz_ values (ppm) on the distance *r_Bq_* (Å) of the ghost atom *Bq* in TSISS***N***U-19 and TISS***N***U-19: (a) the ghost atoms positioned above the silicon spiro center Si_6_, (b) the ghost atom array is set perpendicular to the center of the spiro ring plane, and (c) the *Bq* atom array is set perpendicular to the ring plane and closer to the spiro center (for more details, see the text and Figure 11 and Figure S18). The B3LYP/aug-cc-pVDZ method was used.

	TSISS*N*U-19			TISS*N*U-19		
*r_Bq_*	NICS_zz_^a^	NICS_zz_^b^	NICS_zz_^c^	NICS_zz_^a^	NICS_zz_^b^	NICS_zz_^c^
0.0		5.30	4.10		13.00	11.48
0.5	−4.30	3.87	2.79	−13.30	9.53	8.26
1.0	−3.41	1.54	0.90	−4.83	4.14	3.52
1.5	−10.03	0.19	0.00	−9.38	1.29	1.13
2.0	−5.68	−0.30	−0.31	−6.69	0.25	0.23
2.5	−1.30	−0.41	−0.39	−2.55	−0.12	−0.12
3.0	−0.24	−0.41	−0.38	−0.93	−0.25	−0.25
3.5	−0.12	−0.38	−0.36	−0.47	−0.28	−0.28
4.0	−0.17	−0.34	−0.33	−0.34	−0.27	−0.27
4.5	−0.21	−0.30	−0.29	−0.28	−0.24	−0.25
5.0	−0.23	−0.26	−0.26	−0.24	−0.22	−0.22

## Data Availability

Not applicable.

## Supplementary Materials

The following supporting information can be downloaded at: https://www.mdpi.com/article/10.3390/molecules28176298/s1.
