# Supplementary material for: A Theoretical Investigation of Novel Sila- and Germa-Spirocyclic Imines and Their Relevance for Electron-Transporting Materials and Drug Discovery
Source: Molecules. 2023 Aug 28;28(17):6298. doi: 10.3390/molecules28176298 (PMC10489060; doi:10.3390/molecules28176298)
Supplement: Supplementary file 1 [file molecules-28-06298-s001.zip › molecules-2547122-supplementary.pdf]

Supplementary Material

Theoretical Investigation of Novel Sila- and Germa-spirocyclic Imines and Their Relevance for Electron Transporting Materials and Drug Discovery

Marwan Dakkouri

Department of Electrochemistry, University of Ulm, D-89069 Ulm, Germany; marwan.dakkouri@uni-ulm.de

Table S1. Some structural parameters of *(I)* TISS*N*U-19, *(II)* HIDSS*NC*H-26 and *(III)* OITSSHC-33, *(IV)* DITSSHC-40 (for abbreviations see text and Figure 1). B2PLYP/aug-cc-pVDZ was used. Bond lengths in Å and bond angles in degrees.

Bond lengths	*(I)*	*(II)*	*(III)*	*(IV)*	Bond angles	*(I)*	*(II)*	*(III)*	*(IV)*
$N_1=C_2$	1.288	1.288	1.288	1.288	$\angle N_1=C_2-Si_3$	125.3	125.3	125.4	125.4
$Si_3-C_2=$	1.920	1.921	1.921	1.921	$\angle =C_2-Si_3-C_4=$	106.6	106.6	106.6	106.6
$Si_6-N_1=$	1.770	1.769	1.769	1.768	$\angle Si_6-N_1=C_2$	122.9	122.8	122.8	122.7
$Si_6-N_7=$		1.770	1.770	1.771	$\angle =N_1-Si_6-N_5=$	116.9	117.2	117.1	117.2
$N_7=C_8$		1.287	1.287	1.287	$\angle Si_6-N_7=C_8$		123.2	123.2	123.3
$Si_9-C_8=$		1.921	1.921	1.921	$\angle =N_7-Si_6-N_{16(21)(26)}=$		116.4	116.5	116.4
Si_9-C_{10}		1.921		1.921	$\angle Si_9-C_8=N_7$		125.5	125.4	125.3
$C_{10}=N_{11}$		1.288		1.288	$\angle =C_8-Si_9-C_{15(20)(25)}=$		106.2	106.3	106.4
$=N_{11}-Si_{12}$		1.785		1.769	$\angle =C_{10}-Si_9-C_{14(24)}=$		106.4		106.3
$Si_{12}- N_{13}=$				1.769	$\angle Si_9-C_{10}=N_{11}$		125.5		125.4
$N_{13}=C_{14}$				1.288	$\angle Si_{12}-N_{11}=C_{10}$		123.7		123.0
$Si_{15}-C_{14}=$				1.921	$\angle =N_{11}-Si_{12}-N_{13(23)}=$		115.2		116.7
$Si_{15}-C_{16}=$				1.921	$\angle Si_{12}-N_{13}=C_{14}$				123.1
$C_{16}=N_{17}$				1.288	$\angle Si_{15}-C_{14}=N_{13}$				125.5
$Si_{18}-N_{17}=$				1.786	$\angle =C_{14}-Si_{15}-C_{21}=$				106.2
Si_3-H	1.492	1.492	1.492		$\angle =N_{13}-Si_{12}-N_{22}$				116.6
$Si_{12}-H$		1.483			$\angle Si_{15}-C_{16}=N_{17}$				125.4
					$\angle Si_{18}-N_{17}=C_{16}$				123.8
					$\angle =N_{17}-Si_{18}-N_{19}$				115.2
					$\angle =C_{16}-Si_{15}-C_{20}=$				106.4
					$\angle H-Si_3-H$	108.7	108.8	108.8	108.9
					$\angle H-Si_{12(18)}-H$		111.7		111.8

All rings are planar.

Table S2. Some structural parameters of spiro cyclic-silaimines: *(I)* TSISS*N*U-19, *(II)* HSIDSS*NC*H-26 and *(III)* OSITSSHC-33 (for abbreviations see text and Figure S3). B3LYP/aug-cc-pVDZ was used. Bond lengths in Å and bond angles in degrees.

Bond lengths	*(I)*	*(II)*	*(III)*	Bond angles	*(I)*	*(II)*	*(III)*
$N_{1,5}=Si_{2,4}$	1.618	1.619	1.619	$\angle N_1=Si_2-Si_3$	120.7	120.6	120.7
$Si_3-Si_{2,4}=$	2.369	2.370	2.370	$\angle =Si_2-Si_3-Si_4=$	98.3	98.4	98.3
$Si_6-N_{1,5}=$	1.756	1.753	1.755	$\angle Si_6-N_1=Si_2$	130.9	130.8	130.9
$Si_6-N_7=$		1.758	1.757	$\angle =N_1-Si_6-N_5=$	118.4	118.7	118.5
$N_7=Si_8$		1.616	1.617	$\angle Si_6-N_7=Si_8$		131.3	131.0
$Si_9-Si_8=$		2.373	2.370	$\angle =N_7-Si_6-N_{16(21)}=$		117.3	117.6
Si_9-Si_{10}		2.370		$\angle Si_9-Si_8=N_7$		121.4	121.6
$Si_{10}=N_{11}$		1.621		$\angle =Si_8-Si_9-Si_{15(20)}=$		97.3	97.2
$=N_{11}-Si_{12}$		1.759		$\angle =Si_{10}-Si_9-Si_{14}=$		97.2	

Si_3-H	1.498	1.497	1.497	∠Si_9-Si_{10}=N_{11}		121.8	
Si_{12}-H		1.494		∠=N_{11}-Si_{12}-N_{13}=		117.9	
				∠H-Si_3-H	108.1	108.3	108.2
				∠H-Si_{12}-H		109.5	

All rings are planar.

Table S3. Some structural parameters of spiro cyclic-germaimines ***(I)*** TGIGS*N*U-19, ***(II)*** HGIDGS*NC*H-26 and ***(III)*** OGITGSHC-33 (for these abbreviations see text and Figure S3). B2PLYP/aug-cc-pVDZ was used. Bond lengths in Å and bond angles in degrees.

Bond lengths	*(I)*	*(II)*	*(III)*	Bond angles	*(I)*	*(II)*	*(III)*
$N_{1,5}$=$Ge_{2,4}$	1.731	1.732	1.732	∠N_1=$G2_2$-Ge_3	123.9	123.7	123.8
Ge_3-$Ge_{2,4}$=	2.453	2.453	2.453	∠=Ge_2-Ge_3-Ge_4=	98.7	98.8	98.7
Ge_6-$N_{1,5}$=	1.873	1.870	1.871	∠Ge_6-N_1=Ge_2	124.7	124.6	124.7
Ge_6-N_7=		1.876	1.874	∠=N_1-Ge_6-N_5=	124.2	124.6	124.3
N_7=Ge_8		1.731	1.731	∠Ge_6-N_7=Ge_8		125.1	124.9
Ge_9-Ge_8=		2.459	2.456	∠=N_7-Ge_6-$N_{16(21)}$=		123.3	123.5
Ge_9-Ge_{10}		2.458		∠Ge_9-Ge_8=N_7		124.4	124.4
Ge_{10}=N_{11}		1.731		∠=Ge_8-Ge_9-$Ge_{15(20)}$=		97.8	97.8
=N_{11}-Ge_{12}		1.880		∠=Ge_{10}-Ge_9-Ge_{14}=		97.4	
Ge_3-H	1.541	1.540	1.541	∠Ge_9-Ge_{10}=N_{11}		124.5	
Ge_{12}-H		1.539		∠=N_{11}-Ge_{12}-N_{13}=		121.7	
				∠H-Ge_3-H	108.8	109.1	108.9
				∠H-Ge_{12}-H		111.7	

All rings are planar.

Table S4. Some structural parameters of ***(I)*** TGIGS*N*U-19, ***(II)*** HGIDGS*NC*H-26 and ***(III)*** OGITGSHC-33 (for abbreviations see text and Figure S3). B3LYP/aug-cc-pVDZ was used. Bond lengths in Å and bond angles in degrees.

Bond lengths	*(I)*	*(II)*	*(III)*	Bond angles	*(I)*	*(II)*	*(III)*

$N_{1,5}$=$Ge_{2,4}$	1.719	1.720	1.720	∠N_1=$G2_2$-Ge_3	123.3	123.2	123.2
Ge_3-$Ge_{2,4}$=	2.465	2.466	2.465	∠=Ge_2-Ge_3-Ge_4=	98.5	98.5	98.5
Ge_6-$N_{1,5}$=	1.872	1.869	1.870	∠Ge_6-N_1=Ge_2	126.1	125.9	126.0
Ge_6-N_7=		1.874	1.872	∠=N_1-Ge_6-N_5=	122.8	123.2	122.9
N_7=Ge_8		1.717	1.718	∠Ge_6-N_7=Ge_8		126.6	126.4
Ge_9-Ge_8=		2.478	2.475	∠=N_7-Ge_6-$N_{16(21)}$=		121.7	121.9
Ge_9-Ge_{10}		2.476		∠Ge_9-Ge_8=N_7		123.9	124.0
Ge_{10}=N_{11}		1.720		∠=Ge_8-Ge_9-$Ge_{15(20)}$=		97.3	97.3
=N_{11}-Ge_{12}		1.875		∠=Ge_{10}-Ge_9-Ge_{14}=		97.1	
Ge_3-H	1.546	1.545	1.546	∠Ge_9-Ge_{10}=N_{11}		123.9	
Ge_{12}-H		1.544		∠=N_{11}-Ge_{12}-N_{13}=		120.9	
				∠H-Ge_3-H	108.5	108.7	108.6
				∠H-Ge_{12}-H		111.2	

In comparison to the values which were suggested by B2PLYP/aug-cc-pVDZ (Table S3), these values are generally either evidently smaller than /or equal to those which were predicted by the double-hybrid functional method except for the Ge_3-Ge_4 bond were the B3LYP/aug-cc-pVDZ predict constantly larger values (by 0.02 to 0.03 Å). The valence bond angles vary by 1.3° and 2.1°.

Table S5. Some structural parameters of: *(I)* TCTISS*N*U-23, *(II)* TCOITSSHC-37, *(III)* tetra-isocyanotetraimine (TICTISS*N*U-23), *(IV)* tetraisocyanooctaimino trisilaspiro-hemicosane (TICOITSSHC-37). For abbreviations see text and Figure S5. B3LYP/aug-cc-pVDZ was used. Bond lengths in Å and bond angles in degrees.

Bond lengths	*(I)*	*(II)*	*(III)*	(IV)	Bond angles	*(I)*	*(II)*	*(III)*	(IV)
$N_{1,5}$=$C_{2,4}$	1.275	1.274	1.278	1.277	∠N_1=C_2-Si_3	123.4	123.3	123.4	123.3
Si_3-$C_{2,4}$=	1.921	1.920	1.906	1.905	∠=C_2-Si_3-C_4=	108.0	108.1	108.8	108.9
Si_6-$N_{1,5}$=	1.766	1.771	1.767	1.772	∠Si_6-N_1=C_2	124.7	125.1	124.1	124.5
Si_6-$N_{7,11}$=		1.762		1.763	∠=N_1-Si_6-N_5=	115.9	115.2	116.2	115.6
$N_{7,11}$=$C_{8,10}$		1.280		1.279	∠Si_6-N_7=C_8		123.5		123.5
Si_9-$C_{8,10}$=		1.927		1.926	∠=N_7-Si_6-N_{11}=		116.5		116.5
Si-C≡ (Si-N≡)	1.855	1.857	1.753	1.755	∠Si_9-C_8=N_7		125.2		125.2
C≡N (N≡C)	1.163	1.163	1.185	1.185	∠=C_8-Si_9-C_{10}=		106.0		106.0
					∠≡C-Si_3-C≡	106.6	106.3		
					∠≡N-Si_3-N≡			106.0	105.7

All rings are planar.

Table S6. Selected structural parameters of some tetracyano derivatives of germa-spirocyclic-imines: ***(I)*** TCTIGS***N***U-23 (with one N-Ge-N spiro center), ***(II)*** TCHIDGS***NC***H-30 (with N-Ge-N and C-Ge-C spiro centers), ***(III)*** TCOITGSHC-37 (with alternating N-Ge-N and C-Ge-C- spiro centers). For the abbreviations see text and Figure S1. B2PLYP/aug-cc-pVDZ was used. Bond lengths in Å and bond angles in degrees.

Bond lengths	***(I)***	***(II)***	***(III)***	Bond angles	***(I)***	***(II)***	***(III)***
$N_{1,5}=C_{2,4}$	1.280	1.280	1.279	$\angle N_1=C_2\text{-}Ge_3$	125.0	124.8	124.7
$Ge_3\text{-}C_{2,4}=$	1.991	1.991	1.990	$\angle=C_2\text{-}Ge_3\text{-}C_4=$	108.4	108.5	108.5
$Ge_6\text{-}N_{1,5}=$	1.873	1.876	1.877	$\angle Ge_6\text{-}N_1=C_2$	122.3	122.8	123.0
$Ge_6\text{-}N_7=$		1.870	1.869	$\angle=N_1\text{-}Ge_6\text{-}N_5=$	117.0	116.4	116.1
$N_7=C_8$		1.284	1.285	$\angle Ge_6\text{-}N_7=C_8$		121.4	121.0
$Ge_9\text{-}C_8=$		1.998	1.998	$\angle=N_7\text{-}Ge_6\text{-}N_{16(21)}=$		117.4	117.9
$C_{10}=N_{11}$		1.288		$\angle N_7=C_8\text{-}Ge_9$		126.6	127.0
$N_{11,13}\text{-}Ge_{12}=$		1.870		$\angle=C_8\text{-}Ge_9\text{-}C_{15(20)}=$		106.5	106.2
$Ge_3\text{-}C\equiv$	1.925	1.927	1.927	$\angle=C_{10}\text{-}Ge_9\text{-}C_{14}=$		106.0	
$Ge_{12}\text{-}C\equiv$		1.920		$\angle Ge_9\text{-}C_{10}=N_{11}$		127.5	
$C\equiv N$	1.173		1.174	$\angle C_{10}=N_{11}\text{-}Ge_{12}$		120.3	
				$\angle=N_{11}\text{-}Ge_{12}\text{-}N_{13}=$		118.4	
				$\angle\equiv C\text{-}Ge_3\text{-}C\equiv$	106.0	105.7	105.6
				$\angle\equiv C\text{-}Ge_{12}\text{-}C\equiv$		108.3	

All rings are planar. The Ge-C≡N groups in both compounds ***(I)*** and ***(II)*** are bent outwards by 3.0° and by 2.4° in ***(III)*** as a consequence of the repulsive forces between the cyano groups.

Table S7. Some structural parameters of various fluorine derivatives of sila-spirocyclic octaimine of the type F_x-OITSSHC-33 with alternating N-Si-N and C-Si-C spiro centers (for abbreviations see text and Figure 4). B2PLYP/aug-cc-pVDZ was used. Bond lengths in Å and bond angles in degrees.

Bond lengths	F2-	*term*-F4-	*cent*-F4-	F8-	F12-	Bond angles	F2-	*term*-F4-	*cent*-F4-	F8-	F12-
$N_1=C_2$	1.255	1.289	1.289	1.258	1.258	∠$N_1=C_2$-Si_3	129.8	123.4	125.5	129.8	127.4
Si_3-C_2=	1.917	1.895	1.922	1.920	1.901	∠=C_2-Si_3-C_4=	100.9	109.8	106.4	100.8	104.1
Si_6-N_1=	1.763	1.773	1.761	1.753	1.757	∠Si_6-$N_1=C_2$	122.5	123.3	122.3	121.9	122.7
Si_6-N_7=	1.764	1.767	1.767	1.761	1.757	∠=N_1-Si_6-N_5=	114.5	116.9	118.0	115.7	115.6
$N_7=C_8$	1.289	1.288	1.253	1.255	1.256	∠Si_6-$N_7=C_8$	122.8	122.9	123.4	123.1	122.9
Si_9-C_8=	1.921	1.922	1.909	1.910	1.911	∠=N_7-Si_6-N_{21}	117.2	117.0	113.7	114.3	114.9
Si-F		1.636			1.619	∠Si_9-$C_8=N_7$	125.5	125.4	128.7	128.6	128.5
C-F[(a)]	1.384			1.378	1.367	∠=C_8-Si_9-C_{20}=	106.2	106.4	102.1	102.2	102.3
C-F[(b)]			1.378	1.374	1.371	∠F-Si-F		105.5			108.6
						∠N=C-F[(a)]	118.4			118.3	119.2
						∠Si-C-F[(a)]	111.8			111.9	113.4
						∠N=C-F[(b)]			119.3	119.3	119.4
						∠Si-C-F [(b)]			112.0	112.0	112.1

[(a)]Peripheric C-F bond lengths and N=C-F, Si-C-F bond angles. [(b)]Central C-F bond length N=C-F and Si-C-F bond angles around the C-Si_9-C spiro center.

Positioning of the fluorine substituents in OITSSHC-33:
F2- the two fluorine atoms are located on =C_2 and =C_4.

- *term*-F4- the fluorine atoms are substituted for the hydrogen atoms on the terminal silicon atoms Si_3 and Si_{15}.
- *cent*-F4- the fluorine atoms are attached to the central carbon atoms: C_8, C_{10}, C_{19} and C_{20}.
- F8- the fluorine atoms are positioned on the carbon atoms: C_2, C_4, C_8, C_{10}, C_{14}, C_{16}, C_{19}, and C_{20}.
- F12-the fluorine atoms are placed on the carbon and silicon atoms: 2, 3, 3′, 4, 8, 10, 14, 15, 15′, 16, 19, 20.

For symmetry and redundancy reasons the remaining structural parameters in the spiro chain have been left out.

It is worthwhile to point out that regardless the degree of fluorination of OITSSHC-33 the planarity of the rings remains unaffected despite the electron withdrawal by the strong electronegative fluorine.

Table S8. Structural parameters of various fluorine derivatives of sila-spiro cyclic octaimine of the type F_x-OITSSHC-33 with alternating N-Si-N and C-Si-C spiro centers (for abbreviations see text and Figure 4). B3LYP/aug-cc-pVDZ was used. Bond lengths in Å and bond angles in degrees.

Bond lengths	*term*-F4-	*cent*-F4-	F8-	F12-	Bond angles	*term*-F4-	*cent*-F4-	F8-	F12-
$N_{1,5}=C_{2,4}$	1.281	1.281	1.252	1.253	$\angle N_{1,5}=C_{2,4}-Si_3$	123.3	125.3	129.5	127.0
$Si_3-C_{2,4}=$	1.898	1.924	1.922	1.905	$\angle =C_2-Si_3-C_4=$	109.5	106.2	100.8	104.0
$Si_6-N_{1,5}=$	1.773	1.760	1.752	1.756	$\angle Si_6-N_{1,5}=C_{2,4}$	123.9	122.9	122.7	123.5
$Si_6-N_7=$	1.765	1.765	1.759	1.755	$\angle =N_1-Si_6-N_5=$	116.3	117.4	115.0	114.9
$N_7=C_8$	1.280	1.248	1.249	1.250	$\angle Si_6-N_7=C_8$	123.6	124.1	124.0	123.7
$Si_9-C_8=$	1.926	1.914	1.915	1.916	$\angle =N_7-Si_6-N_{21}=$	116.2	113.1	113.5	114.1
Si-F	1.637			1.621	$\angle Si_9-C_8=N_7$	125.3	128.4	128.3	128.2
C-F[(a)]			1.374	1.364	$\angle =C_8-Si_9-C_{20}=$	106.0	101.9	101.9	102.0
C-F[(b)]		1.375	1.370	1.367	∠F-Si-F	105.4			108.4
					∠N=C-F[(a)]			118.5	119.4
					∠Si-C-F[(a)]			112.1	113.6
					∠N=C-F[(b)]		119.4	119.4	119.4
					∠Si-C-F[(b)]		112.2	112.3	112.3

[(a)]Peripheric C-F bond lengths and N=C-F and Si-C-F bond angles. [(b)]Central C-F bond length N=C-F and Si-C-F bond angles on the C-Si-C spiro center.

As it was indicated in Table S7 the planarity of the rings along the spiro scaffold remains unaffected by the number and position of the fluorine substituents.
For abbreviation see Table S7.

Due to symmetry reasons here and elsewhere in other tables the remaining structural parameters within the chain have been left out.

Table S9. Comparison between the structural parameters of: ***(I)*** 1,4-disila-3,5-disilaiminocyclohexane (DSDSICH-12***),*** ***(II)***1,1,4,4-F4-DSDSICH-12 (*term*-F4-DSDSICH-12)***,*** ***(III)*** 3,5-F_2-DSDSICH-12 (*cent*-F2-DSDSICH-12), ***(IV)***1,1,3,5,4,4-F6-DSDSICH-12 (F6- DSDSICH-12) and those of ***(V)*** TSISS***N***U-19, ***(VI)*** *term-F4*-TSISS***N***U-19, ***(VII)*** *cent*-F4-TSISS***N***U-19, ***(VIII)*** *F8*-TSISS***N***U-19 (for these abbreviations see text and Figure S7). B2LYP/augh-cc-pVDZ was used. Bond lengths in Å and bond angles in degrees.

Bond lengths	*(I)*	*(II)*	*(III)*	*(IV)*	Bond angles	*(I)*	*(II)*	*(III)*	*(IV)*
$N_2=Si_3$	1.630	1.626	1.611	1.605	$\angle N_2=Si_3-Si_4$	121.1	116.7	125.2	120.4
$=Si_3-Si_4$	2.366	2.373	2.353	2.360	$\angle =Si_3-Si_4-Si_5=$	98.5	102.6	94.4	98.5
$Si_1-N_2=$	1.764	1.732	1.766	1.729	$\angle Si_1-N_2=Si_3$	130.1	130.9	128.6	130.2
Si_4-F		1.645		1.632	$\angle =N_2-Si_1-N_6=$	119.0	122.1	118.0	120.3
Si_1-F		1.632		1.628					
Si_3-F			1.638		$\angle =F-Si_4-F$		105.7		107.8
					$\angle =F-Si_1-F$		106.3		106.4
Bond lengths	*(V)*	*(VI)*	*(VII)*	*(VIII)*	Bond angles	*(V)*	*(VI)*	*(VII)*	*(VIII)*
$N_1=Si_2$	1.629	1.627	1.608	1.606	$\angle N_1=Si_2-Si_3$	121.2	117.1	125.0	120.9
$=Si_2-Si_3$	2.365	2.365	2.351	2.355	$\angle =Si_2-Si_3-Si_4=$	98.4	102.8	94.4	98.5
$Si_6-N_1=$	1.759	1.757	1.756	1.754	$\angle Si_6-N_1=Si_2$	129.8	131.4	128.7	130.5
$=Si_2-F$			1.639	1.632	$\angle =N_1-Si_6-N_5=$	119.5	120.2	118.2	118.7
Si_3-F		1.649		1.635	$\angle F-Si_3-F$		104.7		106.8

Table S10. Relevant structural parameters of: ***(I)*** TISS***N***U-19 spirocyclic imine and its various heterocyclic 3,9-derivatives: ***(II)*** diaziridine, ***(III)*** diazetidine, ***(IV)*** disilole, ***(V)*** digermole. ***(VI)*** TITSS***N***DTE-31, ***(VII)*** TITGS***N***DTE-31 (for these abbreviations see text and Figures S8, S9, S15). B3LYP/aug-cc-pVDZ was used. Bond lengths in Å and bond angles in degrees.

Bond lengths	*(I)*	*(II)*	*(III)*	*(IV)*	*(V)*	Bond lengths	*(VI)*	*(VII)*
$N_{1,5}=C_{2,4}$	1.280	1.281	1.281	1.280	1.281	$C_{1,3}=C_{2,4}$	1.356	1.353
C_2-Si_3	1.923	1.927	1.927	1.927	1.925	C_2-C_3	1.485	1.484
$Si_6-N_1=$	1.769	1.771	1.772	1.773	1.773	$=C_4-Si_5/Ge_5$	1.882	1.960
$Si_6-N_5=$		1.769	1.768	1.765	1766	$Si_5/Ge_5-C_6=$	1.926	2.004

Si_3-$N_{azir(azet)}$		1.759	1.741		
$N_{azir(azet)}$-$C_{azir(azet)}$		1.467	1.484		
$(C\text{-}C)_{azir(azet)}$		1.492	1.553		
Si_3-$(Si/Ge)_{sil/ger}$				2.371	2.416
$(Si/Ge)\text{-}C{=})_{sil/ger}$				1.886	1.965
$(C{=}C)_{sil(ger)}$				1.356	1.352
$({=}C\text{-}C)_{sil(ger)}$				1.483	1.484
Bond angles					
∠$N_1{=}C_2$-Si_3	125.2	125.6	126.2	124.8	124.8
∠$={C_2}$-Si_3-$C_4{=}$	106.3	105.4	104.7	104.7	104.9
∠$N_5{=}C_4$-Si_3		125.1	125.7	125.3	124.7
∠Si_6-$N_1{=}C_2$	123.5	122.6	122.3	123.2	122.8
∠Si_6-$N_5{=}C_4$		123.0	122.8	122.5	123.3
∠$={N_1}$-Si_6-$N_5{=}$	116.3	115.8	115.7	114.8	114.9
∠Si_3-$(N\text{-}C)_{azir}$		126.2			
∠Si_3-$(N\text{-}C)_{azet}$			131.3		
$(∠N\text{-}C\text{-}C)_{azir(azet)}$		59.5	89.2		
$(∠C\text{-}N\text{-}C)_{azir(azet)}$		61.0	92.7		
$(∠C\text{-}C\text{-}C)_{azet}$			87.5		
∠Si_3-$(Si/Ge\text{-}C{=})_{sil/ger}$				113.1	
$(∠Si/Ge)\text{-}C{=}C)_{sil/ger}$				106.7	107.2
$(∠C{=}C\text{-}C)_{sil(ger)}$				117.1	118.1
$(∠C\text{-}Si/Ge)\text{-}C)_{sil/ger}$				92.3	89.2
$^{a}\Theta$		14.0	14.0	19.0	18.8
$^{b}\Theta_{azet}$			13.0		

$C_6{=}N_7$	1.279	1.275
$={N_7}{=}Si_8/Ge_8$	1.768	1.879
Bond angles		
∠$C_1{=}C_2$-C_3	116.9	118.0
∠$={C_2}$-C_3-$C_4{=}$	116.9	118.0
∠$C_3{=}C_4$-Si_5/Ge_5	107.0	107.3
∠$={C_4}$-Si_5/Ge_5-$C_1{=}$	92.2	89.3
∠Si_5/Ge_5-$C_6{=}N_7$	125.5	127.1
∠$C_6{=}N_7$-Si_5/Ge_8	123.6	121.8
∠$={N_7}$-Si_8/Ge_8-$N_{18}{=}$	116.0	116.5
∠$={C_{19}}$-Si_5/Ge_5-$C_6{=}$	105.8	105.7

$^{a}\Theta$ = puckering angle of the diimine rings in the silaspiro scaffold.
$^{b}\Theta_{azet}$ = puckering angle of the azetidine ring.

Table S11. Grimme's D3-BJ intramolecular dispersion energy in some silla-/germa-spiro

cyclic-imines and their tetracyano and fluorine derivatives. All energy values in kcal·mol^{-1}. B2PLYP/aug-cc-pVDZ was used.

	E_{D3-BJ}	TC-derivatives[c]	E_{D3-BJ}
TISS***N***U-19[a]	14.5	TC-TISS***N***U-23[a]	20.8
TISS**C**U-19[b]	14.6	TC-TISS***C***U-23[b]	20.8
TIGS***N***U-19[a]	14.5	TC-TIGS***N***U-23[a]	20.6
OITSSHC-33	29.3	TC-OITSSHC-37	35.6
Fx-OITSSHC-33			
term-F4	29.9		
cent-F4	30.0		
F8-	30.4		
F12-	31.1		

[a]N-Si-N (N-Ge-N) spiro center. [b]C-Si-C spiro center. [c]*term*-tetracyano derivatives.

(tetracyano-imines)- (non-substituted-imines) =

1. (20.8) - (14.5) = 6.3 kcal·mol^{-1}
2. (20.8) – (14.6) = 6.2
3. (20.6) – (14.5) = 6.1
4. (35.6) – (29.3) = 6.3

(Fx-OITSSHC-33) – (OITSSHC-33)

1. (term-F4): 29.9 - 29.3= 0.6 kcal·mol^{-1}
2. (cent-F4): 30.0 - 29.3 = 0.7
3. (F8-): 30.4 - 29.3 =1.1
4. (F12-): 31.1 - 29.3 = 1.8

Table S12. Natural charges q_{NPA} in various fluorine derivatives of OITSSHC-33 of the type F_x- OITSSHC-33 (for abbreviations see text and Figure 4). B2PLYP/aug-cc-pVDZ was used.

Atom	*term*-F4-	*cent*-F4-	F8-	F12-
$N_{1,5}$	-0.870	-0.902	-0.943	-0.923
$C_{2,4}$	-0.324	-0.212	0.380	0.290
$Si_{3,15}$	2.199	1.098	1.077	2.204
$Si_{6,12}$	2.258	2.277	2.289	2.284
Si_{9}	1.457	1.410	1.409	1.408
$N_{7,21}$	-0.883	-0.903	-0.907	-0.911
$C_{8,20}$	-0.239	0.351	0.359	0.364
F[(a)]	-0.674			-0.657
F[(b)]			-0.384	-0.380
F[(c)]		-0.391	-0.394	-0.380

[(a)]the fluorine atoms are geminally positioned on the terminal silicon atoms Si_3 and Si_{15}.

[(b)]the fluorine atoms are residing on the central carbon atoms: C_8, C_{10}, C_{19}, C_{20}.

[(c)]the fluorine atoms are positioned on the carbon atoms: C_2, C_4, C_{14}, C_{16}.

Table S13. Mulliken charges q_M in various fluorine derivatives of silaspiro cyclic octaimine of the type F_x-OITSSHC-33 (for abbreviations see text and Figure 4). B2PLYP/aug-cc-pVDZ was used.

Atom	*term*-F4-	*cent*-F4-	F8-	F12-
$N_{1,5}$	-0.470	-0.467	-0.615	-0.598
$C_{2,4}$	0.271	0.314	0.494	0.537

$Si_{3,15}$	1.920	-0.200	-0.339	1.713
$Si_{6,12}$	2.396	2.496	2.485	2.354
Si_9	0.912	0.040	0.058	0.159
$N_{7,21}$	-0.564	-0.859	-0.821	-0.771
$C_{8,20}$	0.360	0.697	0.723	0.689
$H_{(Si)}$		0.334	0.343	
F[(a)]	-0.784			-0.748
F[(b)]			-0.613	-0.607
F[(c)]		-0.620	-0.599	-0.576

[(a)]the fluorine atoms are geminally positioned on the terminal silicon atoms Si_3 and Si_{15}.
[(b)]the fluorine atoms are residing on the central carbon atoms: C_8, C_{10}, C_{19}, C_{20}.
[(c)]the fluorine atoms are positioned on the carbon atoms: C_2, C_4, C_{14}, C_{16}.

Table S14. Mulliken charges q_M in sila- and germa-spirocyclic imines: ***(I)*** TISS*N*U-19, ***(II)*** TCSSTI*N*U-23, ***(III)*** OITSSHC-33, ***(IV)*** TCOITSSHC-37, ***(V)*** TIGS*N*U-19, ***(VI)*** TCTGS*N*U-23. The B3LYP/aug-cc-pVDZ method was used.

Atom	***(I)***[(a)]	***(II)***[(a)]	Atom	***(III)***[(b)]	***(IV)***[(b)]	Atom	***(V)***[(c)]	***(VI)***[(c)]
$N_{1,5}$	-0.326	-0.277	$N_{1,5}$	-0.263	-0.284	$N_{1,5}$	-0.454	-0.440
$C_{2,4}$	0.438	0.380	$C_{2,4}$	0.390	0.358	$C_{2,4}$	0.391	0.370
$Si_{3,9}$	-0.677	1.075	Si_3	-0.700	0.961	$Ge_{3,9}$	-0.410	1.021
Si_6	1.591	1.765	Si_6	1.898	1.914	Ge_6	2.081	2.466
$H_{(C)}$	-0.642	-0.674	N_7	-0.367	-0.249	$H_{(C)}$	-0.627	-0.654
$H_{(Si)}$	0.471		C_8	0.421	0.389	$H_{(Si)}$	0.375	
C≡		-0.067	Si_9	0.771	0.857	C≡		-0.058
≡N		-0.340	$H_{(C)}$	-0.659	-0.686	≡N		-0.345
			$H_{(Si)}$	0.483				
			C≡		-0.022			
			≡N		-0.352			

[(a)]N-Si-N spiro center, [(b)]alternating N-Si-N and C-Si-C spiro centers, [(c)]N-Ge-N spiro center.

Table S15. Natural charges q_{NPA} in various fluorine derivatives of silaspiro cyclic octaimine of the type F_x- OITSSHC-33 (for abbreviations see text and Figure 4). B3LYP/aug-cc-pVDZ was used.

Atom	*term-*	*cent-*	F8-[(c)]	F12-

	F4-[(a)]	F4-[(b)]		
$N_{1,5}$	-0.833	-0.859	-0.896	-0.882
$C_{2,4}$	-0.324	-0.216	0.348	0.258
$Si_{3,15}$	2.109	1.008	0.992	2.108
$Si_{6,12}$	2.170	2.191	2.205	2.201
Si_{9}	1.358	1.304	1.303	1.302
$N_{7,21}$	-0.844	-0.863	-0.866	-0.869
$C_{8,20}$	-0.235	0.328	0.334	0.339
F[(a)]	-0.648			-0.630
F[(b)]			-0.362	-0.347
F[(c)]		-0.359	-0.353	-0.348

[(a)]the fluorine atoms are geminally positioned on the terminal silicon atoms Si_3 and Si_{15}.
[(b)]the fluorine atoms are residing on the central carbon atoms: C_8, C_{10}, C_{19}, C_{20}.
[(c)]the fluorine atoms are positioned on the carbon atoms: C_2, C_4, C_{14}, C_{16}.

A brief comparison of the above listed values with the corresponding values in Table 12 (where B2PLYP/aug-pVDZ was used) reveals that the NPA charges which have been produces by both functional methods exhibit the same trend and in good agreement with each other but the B3LYP method suggests generally slightly smaller values. This is at variance with the behavior of the Mulliken charge distribution upon using these computational methods as is shown and commented in Tables S13 and S16.

Table S16. Mulliken charges q_M in various fluorine derivatives of sila-spirocyclic octaimine of the type F_x-OITSSHC-33 (for abbreviations see text and Figure 4). B3LYP/aug-cc-pVDZ was used.

Atom	*term*-F4-	*cent*-F4-	F8-	F12-
$N_{1,5}$	-0.247	-0.243	-0.413	-0.390
$C_{2,4}$	0.348	0.422	0.445	0.458
$Si_{3,15}$	1.610	-0.695	-0.842	1.378
$Si_{6,12}$	1.818	1.892	1.871	1.751
Si_{9}	0.756	0.025	0.062	0.111
$N_{7,21}$	-0.318	-0.643	-0.601	-0.555
$C_{8,20}$	0.429	0.559	0.586	0.564
$H_{(Si)}$		0.491	0.491	
F[(a)]	-0.699			-0.662
F[(b)]			-0.513	-0.485
F[(c)]		-0.533	-0.526	-0.520

[(a)]the fluorine atoms are geminally positioned on the terminal silicon atoms Si_3 and Si_{15}.
[(b)]the fluorine atoms are residing on the central carbon atoms: C_8, C_{10}, C_{19}, C_{20}.
[(c)]the fluorine atoms are positioned on the carbon atoms: C_2, C_4, C_{14}, C_{16}.

Approximately similar tendency of the compared values for the NPA charges (Tables S12 and S16) is shown by the Mulliken charges which are displayed in Tables S13 (where B2PLYP/aug-cc-pVDZ was used) and S16, however, the differences between the values are inconsistent (some are smaller and others are larger). It remains to mention that in consistency with the double-hybrid functional (Table S13), the B3LYP functional suggests also negative (but significantly larger) values for the Mulliken charges on the terminal silicon atoms, $Si_{3,15}$, in the *cent*-F4- and F8-derivatives of OITSSHC-33 of -0.695 e^- and -0.842 e^- (Table S16), respectively.

Table S17. Natural charges q_{NPA} in spiro-cyclic silaimines: ***(I)*** TSISS*N*U-19, (***II)*** OSITSSHC-33 as suggested by B3LYP/aug-cc-pVDZ and ***(III***) OSITSSHC-33 as predicted by B2PLYP/aug-cc-pVDZ. For abbreviations see Figures 2 and S3.

Atom	*(I)*	*(II)*	*(III)*
$N_{1,5}$	-1.483	-1.486	-1.544
$Si_{2,4}$	1.189	1.193	1.239
$Si_{3,9}$	0.038		
$Si_{3,15}$		0.038	0.096
Si_6	2.258	2.259	2.342

$N_{7,21}$		-1.473	-1.531
$Si_{8,20}$		1.243	1.294
Si_{9}*		-0.667	-0.651
$H^{a}_{(Si)}$	-0.180	-0.171	-0.128

(a)Terminal hydrogens.

*Mulliken population analysis predicts a charge of -0.275 e^- on Si_9 by using B2PLYP/aug-cc-pVDZ and -0.162e^- at the B3LYP/aug-cc-pVDZ level of theory.

Table S18. Some relevant results of the NBO occupancy analysis of sila-spirocyclic imine: ***(I)*** TISS***N***U-19 and its tetracyano derivative ***(II)*** TCTISS***N***U-23 with N-Si-N spiro center, B3LYP/cc-pVDZ level of theory was applied. ξ = polarization coefficient.

Bond Orbital		*(I)*	*(II)*	Bond Orbital		*(II)*
N_1-C_2(σ)	occ.	1.995	1.994	Si-C≡	occ.	1.973

	% N_1	60.71	60.67		% Si	28.99
	% C_2	39.29	39.33		% C≡	71.01
	ξ(N_1)	0.779	0.779		ξ(Si)	0.539
	ξ(C_2)	0.627	0.627		ξ(C≡)	0.843
N_1=C_2(π)	occ.	1.955	1.950	C≡N (σ)	occ.	1.998
	% N_1	57.93	57.63		% C	40.45
	% C_2	42.07	42.37		% N	59.55
	ξ(N_1)	0.761	0.759		ξ(C)	0.636
	ξ(C_2)	0.649	0.651		ξ(N)	0.772
=N_1-Si_6	occ.	1.947	1.962	C≡N (π_2)	occ.	1.980
	% N_1	80.63	80.59		% C	47.96
	% Si_6	19.37	19.41		% N	52.04
	ξ(N_1)	0.898	0.898		ξ(C)	0.693
	ξ(Si_6)	0.440	0.441		ξ(N)	0.721
Si_3-C_4=	occ.	1.980	1.961			
	% Si_3	31.40	32.57			
	% C_4	68.60	67.43			
	ξ(Si_3)	0.560	0.571			
	ξ(C_4)	0.828	0.821			
(LP)N_1	occ.	1.881	1.871	(LP)$N_{\equiv 17,19}$	1.969	

Table S19. NBO occupancy values as provided by the NBO analysis of: ***(I)*** F8-TSISS***N***U-19 and ***(II)*** F8-TISS***N***U-19 (both with N-Si-N spiro-center).

B3LYP/aug-cc-pVDZ level of theory was applied. ξ = polarization coefficient.

Bond Orbital		*(I)*	Bond Orbital		*(II)*
N_1-Si_2(σ)	occ.	1.973	N_1-C_2(σ)	occ.	1.993
	% N_1	78.80		% N_1	60.80
	% Si_2	21.20		%C_2	39.20
	ξ(N_1)	0.888		ξ(N_1)	0.780
	ξ(Si_2)	0.461		ξ(C_2)	0.626
N_1=Si_2(π)	occ.	1.939	N_1=C_2(π)	occ.	1.953
	% N_1	77.00		% N_1	60.56
	% C_2	23.00		%C_2	39.44
	ξ(N_1)	0.877		ξ(N_1)	0.778
	ξ(Si_2)	0.480		ξ(C_2)	0.628
=N_1-Si_6	occ.	1.955	=N_1-Si_6	occ.	1.914
	% N_1	82.48		% N_1	81.39
	% Si_6	17.52		% Si_6	18.61
	ξ(N_1)	0.908		ξ(N_1)	0.902
	ξ(Si_6)	0.419		ξ(Si_6)	0.431
=Si_2-Si_3	occ.	1.856	=C_2-Si_3	occ.	1.942
	% Si_2	50.15		% C_2	73.21
	% Si_3	49.85		% Si_3	26.79
	ξ(Si_2)	0.708		ξ(C_2)	0.856
	ξ(Si_3)	0.706		ξ(Si_3)	0.518
Si_2-F	occ.	1.991	C_2-F	occ.	1.994

	% Si_2	13.22		% C_2	26.76
	% F	86.78		% F	73.24
	ξ(Si_2)	0.364		ξ(C_2)	0.517
	ξ(F)	0.932		ξ(F)	0.856
Si_3-F_{17}	occ.	1.988	Si_3-F_{17}	occ.	1.985
	% Si_3	12.82		% Si_3	12.86
	% F	87.18		% F	87.14
	ξ(Si_3)	0.358		ξ(Si_3)	0.359
	ξ(F)	0.934		ξ(F)	0.934
(LP)N_1	occ.	1.843	(LP)N_1	occ.	1.842
(LP_3)F_{15}	occ.	1.935	(LP_3)F_{15}	occ.	1.892
(LP_2)F_{17}	occ.	1.954	(LP_2)F_{17}	occ.	1.951
(LP_3)F_{17}	occ.	1.946	(LP_3)F_{17}	occ.	1.945

2nd-order perturbative energy values E(2) of NBO interactions in the spiro cyclic silaimine F8-TSISS***N***U-19:

Donor orbital	Acceptor orbital	F8-TSISS***N***U-19 E(2)
π_{N1-C2}	$\sigma^*_{Si6-N7,21}$	5.05
$\sigma_{Si2-Si3}$	σ^*_{N1-Si2}	9.85
$\sigma_{Si2-Si3}$	$\sigma^*_{Si2-F19}$	10.49
$\sigma_{Si2-Si3}$	$\sigma^*_{Si2-F17,18}$	7.72
$\sigma_{Si3-F17}$	$\sigma^*_{Si3-F18}$	2.78
$(LP)_{N1}$	σ^*_{Si2-S3}	10.63
$(LP)_{N1}$	σ^*_{N5-Si6}	10.22
$(LP_3)_{F17}$	$\sigma^*_{Si3-F18}$	10.25
$(LP_3)_{F18}$	$\sigma^*_{Si3-F17}$	10.25
$(LP_3)_{F15}$	σ^*_{N7-Si8}	14.30

Table S20. Dependency of the NBO donor-acceptor stabilization energies E(2) as predicted by the SOPT analysis on the applied computational method and basis set for: ***(I)*** TISS***N***U-19 and ***(II)*** TIGS***N***U-19**.** E(2) values are in kcal·mol^{-1}. For atomic numbering see Figures 1 and S1.

Donor orbital	Acceptor orbital	E(2)	Donor orbital	Acceptor orbital	E(2)
	(I)[a]			***(II)***[b]	

B3LYP/aug-cc-pVDZ					
$\pi_{(2)N1=C2}$	$\sigma^*_{Si6-N7,11=}$	3.3	$\pi_{(2)N1=C2}$	$\sigma^*_{Ge6-N7,1=}$	2.6
$\sigma_{Si6-N1=}$	$\sigma^*_{Si6-N5=}$	3.6	$\sigma_{Ge6-N1=}$	$\sigma^*_{Ge6-N5=}$	6.5
$\sigma_{Si6-N1=}$	$\sigma^*_{Si6-N7,11=}$	2.1	$\sigma_{Ge6-N1=}$	$\sigma^*_{Ge6-N7,11=}$	3.8
σ_{C2-Si3}	$\sigma^*_{Si3-C4=}$	2.5	σ_{C2-Ge3}	$\sigma^*_{Ge3-C4=}$	4.0
$(LP)_{N1}$	$\sigma^*_{Si3-C2=}$	12.2	$(LP)_{N1}$	$\sigma^*_{Ge3-C2=}$	13.1
$(LP)_{N1}$	$\sigma^*_{Si6-N5=}$	8.4	$(LP)_{N1}$	$\sigma^*_{Ge6-N5=}$	6.5
B3LYP/cc-pVDZ					
$\pi_{(2)N1=C2}$	$\sigma^*_{Si6-N7,11=}$	3.1	$\pi_{(2)N1=C2}$	$\sigma^*_{Ge6-N7,1=}$	2.6
$\sigma_{Si6-N1=}$	$\sigma^*_{Si6-N5=}$	3.6	$\sigma_{Ge6-N1=}$	$\sigma^*_{Ge6-N5=}$	6.6
$\sigma_{Si6-N1=}$	$\sigma^*_{Si6-N7,11=}$	2.2	$\sigma_{Ge6-N1=}$	$\sigma^*_{Ge6-N7,11=}$	4.0
σ_{C2-Si3}	$\sigma^*_{Si3-C4=}$	2.1	σ_{C2-Ge3}	$\sigma^*_{Ge3-C4=}$	3.4
$(LP)_{N1}$	$\sigma^*_{Si3-C2=}$	13.2	$(LP)_{N1}$	$\sigma^*_{Ge3-C2=}$	14.1
$(LP)_{N1}$	$\sigma^*_{Si6-N5=}$	7.5	$(LP)_{N1}$	$\sigma^*_{Ge6-N5=}$	6.0
B2PLYP/aug-cc-pVDZ					
$\pi_{(2)N1=C2}$	$\sigma^*_{Si6-N7,11=}$	4.0	$\pi_{(2)N1=C2}$	$\sigma^*_{Ge6-N7,1=}$	3.2
$\sigma_{Si6-N1=}$	$\sigma^*_{Si6-N5=}$	3.9	$\sigma_{Ge6-N1=}$	$\sigma^*_{Ge6-N5=}$	6.7
$\sigma_{Si6-N1=}$	$\sigma^*_{Si6-N7,11=}$	2.4	$\sigma_{Ge6-N1=}$	$\sigma^*_{Ge6-N7,11=}$	3.9
σ_{C2-Si3}	$\sigma^*_{Si3-C4=}$	2.9	σ_{C2-Ge3}	$\sigma^*_{Ge3-C4=}$	4.2
$(LP)_{N1}$	$\sigma^*_{Si3-C2=}$	13.0	$(LP)_{N1}$	$\sigma^*_{Ge3-C2=}$	14.1
$(LP)_{N1}$	$\sigma^*_{Si6-N5=}$	9.8	$(LP)_{N1}$	$\sigma^*_{Ge6-N5=}$	7.6
HF/6-311G(d,p)					
$\pi_{(2)N1=C2}$	$\sigma^*_{Si6-N7,11=}$	4.1	$\pi_{(2)N1=C2}$	$\sigma^*_{Ge6-N7,1=}$	3.5
$\sigma_{Si6-N1=}$	$\sigma^*_{Si6-N5=}$	2.9	$\sigma_{Ge6-N1=}$	$\sigma^*_{Ge6-N5=}$	4.9
$\sigma_{Si6-N1=}$	$\sigma^*_{Si6-N7,11=}$	1.3	$\sigma_{Ge6-N1=}$	$\sigma^*_{Ge6-N7,11=}$	2.4
σ_{C2-Si3}	$\sigma^*_{Si3-C4=}$	2.4	σ_{C2-Ge3}	$\sigma^*_{Ge3-C4=}$	3.0
$(LP)_{N1}$	$\sigma^*_{Si3-C2=}$	15.1	$(LP)_{N1}$	$\sigma^*_{Ge3-C2=}$	15.9
$(LP)_{N1}$	$\sigma^*_{Si6-N5=}$	9.2	$(LP)_{N1}$	$\sigma^*_{Ge6-N5=}$	7.9

[a]N-Si-N spiro center. [b]N-Ge-N spiro center.

Table S21. HOMO and LUMO frontier molecular orbitals and energy gaps in a variety of sila-spirocyclic imines as predicted by different computational methods (for abbreviations see text and Figures 7 and S13).

	B3LYP/ cc-pVDZ			B2PLYP/ cc-pVDZ			MP2/ cc-pVDZ		
	E_{HOMO} (a.u)	E_{LUMO} (a.u)	E_{gap} (eV)	E_{HOMO} (a.u)	E_{LUMO} (a.u)	E_{gap} (eV)	E_{HOMO} (a.u)	E_{LUMO} (a.u)	E_{gap} (eV)
TISS***N***U-19[(a)]	-0.215	-0.061	4.19	-0.267	0.004	7.37	-0.346	0.092	11.92
TISS***C***U-19[(b)]	-0.221	-0.076	3.95	-0.276	-0.016	7.07	-0.357	0.069	11.59
TIGS***N***U-19[(a)]	-0.210	-0.052	4.30	-0.260	0.010	7.35	-0.335	0.097	11.76
TCTISS***N***-23[(a)]	-0.260	-0.113	4.00	-0.314	-0.053	7.10	-0.394	0.031	11.56
TCTIGS***N***-23[(c)]	-0.259	-0.109	4.08	-0.309	-0.050	7.05	-0.386	0.034	11.43
OITSSHC-33[(d)]	-0.217	-0.079	3.76	-0.271	-0.018	6.88	-0.350	0.068	11.37
term-F4-[(e)]	-0.232	-0.093	3.78	-0.287	-0.034	6.88	-0.368	0.050	11.37
cent-F4[(e)]	0.228	-0.076	4.14	-0.283	-0.021	7.13	-0.364	0.061	11.56

[(a)]N-Si-N spiro center. [(b)]C-Si-C spiro center. [(c)]N-Ge-N spiro center. [(d)]Alternating N-Si-N and C-Si-C spiro centers. [(e)]Derivatives of OITSSHC-33.

Table S22: HOMO and LUMO frontier molecular orbitals and energy gaps in a variety of sila-spirocyclic imines as predicted by different computational methods (for abbreviations see text and Figures 7, S13).

	B3LYP/ 6-31+G(d,p)			B3LYP/ 6-311+G(d,p)			B3LYP/ 6-311G(d,p)		
	E_{HOMO} (a.u)	E_{LUMO} (a.u)	E_{gap} (eV)	E_{HOMO} (a.u)	E_{LUMO} (a.u)	E_{gap} (eV)	E_{HOMO} (a.u)	E_{LUMO} (a.u)	E_{gap} (eV)
TISS***N***U-19	-0.222	-0.072	4.08	-0.222	-0.073	4.05	-0.219	-0.067	4.14
TISS***C***U-19	-0.231	-0.090	3.84	-0.231	-0.091	3.81	-0.227	-0.083	3.92
TIGS***N***U-19	-0.219	-0.062	4.27	-0.218	-0.061	4.27	-0.215	-0.056	4.33
TCTISS***N***U-23	-0.270	-0.124	3.97	-0.270	-0.125	3.95	-0.265	-0.120	3.95
TCTIGS***N***U-23	-0.270	-0.118	4.14	-0.270	-0.119	4.11	-0.265	-0.114	4.11
OITSSHC-33	-0.226	-0.091	3.67	-0.226	-0.092	3.65	-0.223	-0.087	3.70
term-F4-[(a)]	-0.241	-0.105	3.70	-0.242	-0.107	3.67	-0.242	-0.107	3.67
cent-F4-[(a)]	-0.238	-0.091	4.00	-0.238	-0.091	4.00	-0.234	-0.085	4.05

[(a)]Derivatives of OITSSHC-33.

Table S23. Comparison of the atomic and critical point properties of OITSSHC-33 (Figure 1) as obtained from B2PLYP in combination with the basis sets: *(I)* cc-pVDZ, *(II)* 6311+G**, *(III)* 6-311G**), *(IV)* 6-31+G* and *(V)* 6-31G*. All quantities are in atomic units. For abbreviations see text.

	r_A	r_B	$\varrho(r_c)$	λ_1	λ_2	λ_3	$\nabla^2\varrho(r_c)$	ε_c
(I)								
N_1=C_2	1.605	0.829	0.363	-0.756	-0.698	0.645	-0.809	0.083
C_2-Si_3	2.256	1.373	0.107	-0.136	-0.131	0.551	0.284	0.039
N_1-Si_6	2.029	1.319	0.114	-0.178	-0.167	0.951	0.606	0.066
Si_6-N_7	1.320	2.029	0.114	-0.177	-0.166	0.946	0.604	0.066
C-$H_{(av)}$	1.330	0.707	0.271	-0.676	-0.673	0.369	-0.980	0.005
Si-H	1.363	1.424	0.110	-0.150	-0.149	0.590	0.291	0.003
(II)								
N_1=C_2	1.556	0.868	0.366	-0.782	-0.712	0.697	-0.798	0.097
C_2-Si_3	2.241	1.371	0.116	-0.160	-0.155	0.520	0.206	0.036
N_1-Si_6	2.004	1.304	0.129	-0.218	-0.207	0.992	0.567	0.056
Si_6-N_7	1.305	2.006	0.128	-0.217	-0.205	0.987	0.566	0.058
C-$H_{(av)}$	1.340	0.713	0.276	-0.728	-0.726	0.518	-0.936	0.002
Si-H	1.355	1.428	0.119	-0.182	-0.181	0.604	0.242	0.003
(III)								
N_1=C_2	1.555	0.867	0.366	-0.783	-0.710	0.695	-0.798	0.103
C_2-Si_3	2.240	1.371	0.116	-0.161	-0.155	0.518	0.202	0.037
N_1-Si_6	2.002	1.303	0.129	-0.220	-0.207	0.999	0.571	0.060
Si_6-N_7	1.304	2.002	0.129	-0.219	-0.206	0.995	0.570	0.062
C-$H_{(av)}$	1.339	0.715	0.276	-0.726	-0.725	0.515	-0.936	0.002
Si-H	1.355	1.428	0.119	-0.181	-0.181	0.605	0.243	0.002
(IV)								
N_1=C_2	1.603	0.831	0.367	-0.793	-0.719	0.780	-0.732	0.102
C_2-Si_3	2.240	1.371	0.112	-0.154	-0.149	0.526	0.224	0.034
N_1-Si_6	2.035	1.311	0.123	-0.203	-0.192	0.945	0.550	0.058
Si_6-N_7	1.312	2.037	0.122	-0.202	-0.190	0.940	0.548	0.060
C-$H_{(av)}$	1.356	0.703	0.270	-0.727	-0.726	0.530	-0.924	0.001
Si-H	1.374	1.428	0.116	-0.165	-0.165	0.469	0.139	0.000
(V)								
N_1=C_2	1.599	0.829	0.368	-0.797	-0.721	0.803	-0.715	0.105
C_2-Si_3	2.247	1.365	0.114	-0.157	-0.152	0.546	0.237	0.033
N_1-Si_6	2.010	1.301	0.126	-0.214	-0.201	1.030	0.610	0.062
Si_6-N_7	1.302	2.012	0.125	-0.213	-0.200	1.020	0.607	0.064
C-$H_{(av)}$	1.346	0.702	0.273	-0.738	-0.737	0.530	-0.944	0.003
Si-H	1.369	1.420	0.117	-0.167	-0.166	0.492	0.159	0.001

Table S24. Atomic and critical point properties of some bonds in silaspiro cyclic imines: TISS***N***U-19 and TISS***C***U-19 and their tetracyano derivatives TCTISS***N***U-23, and TCTISS***C***U-23 (Figures 1 and S5) as obtained from the B2PLYP/aug-cc-pVDZ method. All quantities are in atomic units. For abbreviations see text.

	r_A	r_B	$\varrho(r_c)$	λ_1	λ_2	λ_3	$\nabla^2\varrho(r_c)$	ε_c
TISS***N***U-19								
N_1=C_2	1.598	0.835	0.365	-0.775	-0.722	0.593	-0.905	0.073
C_2-Si_3	2.256	1.373	0.107	-0.138	-0.133	0.555	0.284	0.034
N_1-Si_6	2.029	1.320	0.115	-0.179	-0.169	0.946	0.598	0.059
Si_6-N_7	1.320	2.029	0.115	-0.179	-0.169	0.946	0.598	0.059
TCTISS***N***U-23								
N_1=C_2	1.595	0.832	0.368	-0.784	-0.724	0.623	-0.886	0.082
C_2-Si_3	2.250	1.370	0.112	-0.144	-0.141	0.546	0.261	0.019
N_1-Si_6	2.025	1.318	0.115	-0.181	-0.170	0.955	0.604	0.066
Si_6-N_7	1.318	2.025	0.115	-0.181	-0.170	0.955	0.604	0.066
Si_3-C_{12}≡	1.356	2.151	0.104	-0.142	-0.137	0.682	0.404	0.035
C≡N	0.743	1.475	0.455	-0.986	-0.983	2.231	0.263	0.003
TISS***C***U-19								
C_1=N_2	0.834	1.600	0.365	-0.772	-0.717	0.607	-0.882	0.077
N_2-Si_3	2.047	1.330	0.109	-0.162	-0.160	0.886	0.564	0.014
C_1-Si_6	2.256	1.375	0.107	-0.136	-0.130	0.544	0.279	0.047
Si_6-C_7	1.375	2.256	0.107	-0.136	-0.130	0.544	0.279	0.047
TCTISS***C***U-23								
C_1=N_2	0.835	1.602	0.363	-0.764	-0.722	0.599	-0.887	0.058
N_2-Si_3	2.019	1.316	0.117	-0.180	-0.175	0.959	0.605	0.029
C_1-Si_6	2.260	1.376	0.107	-0.135	-0.129	0.539	0.275	0.043
Si_6-C_7	1.376	2.260	0.107	-0.135	-0.129	0.539	0.275	0.043
Si_3-C_{12}≡	1.350	2.147	0.106	-0.152	-0.147	0.706	0.407	0.037
C≡N	0.742	1.473	0.456	-0.988	-0,985	2.252	0.278	0.003

Table S25. Comparison of atomic and critical point properties of some bonds in TCOITSSHC-37 as obtained from B2PLYP in combination with the basis sets: ***(I)*** 6-311+G**, ***(II)*** 6-311G**, ***(III)*** 6-311+G*, ***(IV)*** 6-311G*, ***(V)*** 6-31+G* and ***(VI)*** 6-31G*. All quantities are in atomic units. For abbreviations see text.

	r_A	r_B	$\varrho(r_c)$	λ_1	λ_2	λ_3	$\nabla^2\varrho(r_c)$	ε_c
(I)								
N_1=C_2	1.556	0.870	0.366	-0.783	-0.701	0.681	-0.803	0.117
C_2-Si_3	2.244	1.373	0.119	-0.163	-0.159	0.489	0.167	0.024
N_1-Si_6	2.035	1.317	0.123	-0.203	-0.191	0.902	0.507	0.063
Si_6-N_7	1.312	2.025	0.126	-0.208	-0.198	0.927	0.521	0.055
Si_3-C≡	1.360	2.149	0.111	-0.160	-0.155	0.624	0.310	0.033
C≡N	0.778	1.440	0.458	-0.999	-0.996	1.765	-0.230	0.003
(II)								
N_1=C_2	1.551	0.863	0.369	-0.793	-0.712	0.722	-0.783	0.114
C_2-Si_3	2.234	1.369	0.121	-0.166	-0.162	0.506	0.178	0.022
N_1-Si_6	2.005	1.305	0.127	-0.217	-0.204	0.987	0.566	0.068
Si_6-N_7	1.300	2.995	0.131	-0.222	-0.210	1.101	0.582	0.060
Si_3-C≡	1.352	2.127	0.114	-0.167	-0.162	0.657	0.329	0.033
**C≡N**	0.772	1.430	0.466	-1.032	-1.030	1.890	-0.172	0.003
(III)								
N_1=C_2	1.555	0.870	0.365	-0.782	-0.702	0.679	-0.805	0.114
C_2-Si_3	2.243	1.374	0.119	-0.162	-0.159	0.485	0.164	0.022
N_1-Si_6	2.035	1.317	0.123	-0.203	-0.192	0.902	0.507	0.063
Si_6-N_7	1.312	2.025	0.126	-0.209	-0.198	0.928	0.521	0.055
Si_3-C≡	1.360	2.149	0.111	-0.160	-0.155	0.624	0.310	0.033
C≡N	0.778	1.440	0.458	-0.999	-0.996	1.765	-0.230	0.003
(IV)								
N_1=C_2	1.555	0.870	0.365	-0.781	-0.699	0.675	-0.805	0.117
C_2-Si_3	2.243	1.374	0.119	-0.163	-0.159	0.484	0.162	0.023
N_1-Si_6	2.036	1.317	0.123	-0.204	-0.192	0.901	0.505	0.066
Si_6-N_7	1.312	2.025	0.126	-0.209	-0.198	0.926	0.519	0.059
Si_3-C≡	1.360	2.149	0.112	-0.161	-0.156	0.620	0.304	0.032
C≡N	0.778	1.440	0.458	-0.996	-0.993	2.177	-0.223	0.003
(V)								
N_1=C_2	1.597	0.828	0.370	-0.805	-0.719	0.814	-0.709	0.120
C_2-Si_3	2.250	1.367	0.118	-0.160	-0.157	0.509	0.193	0.018
N_1-Si_6	2.039	1.314	0.121	-0.201	-0.188	0.934	0.545	0.067
Si_6-N_7	1.309	2.028	0.124	-0.206	-0.194	0.962	0.562	0.059
Si_3-C≡	1.352	2.157	0.110	-0.160	-0.155	0.660	0.344	0.031
C≡N	0.751	1.467	0.456	-0.971	-0.968	2.171	0.233	0.003
(VI)								

$N_1=C_2$	1.597	0.828	0.370	-0.805	-0.715	0.815	-0.705	0.126
C_2-Si_3	2.250	1.367	0.118	-0.160	-0.157	0.511	0.194	0.018
N_1-Si_6	2.040	1.314	0.121	-0.200	-0.187	0.936	0.548	0.070
Si_6-N_7	1.309	2.029	0.124	-0.205	-0.193	0.964	0.565	0.062
Si_3-C≡	1.352	2.157	0.110	-0.161	-0.156	0.660	0.343	0.031
C≡N	0.750	1.468	0.456	-0.967	-0.964	2.210	0.278	0.003

Table S26. Atomic and critical point properties of some bonds in various fluorine derivatives of OITSSHC-33 (Figure 4). B2PLYP/aug-cc-pVDZ computational method was used. All quantities are in atomic units. For abbreviations see text.

	r_A	r_B	$\rho(r_c)$	λ_1	λ_2	λ_3	$\nabla^2\rho(r_c)$	ε_c
term-F4-								
$N_1=C_2$	1.599	0.837	0.365	-0.774	-0.716	0.578	-0.912	0.080
C_2-Si_3	2.226	1.355	0.116	-0.160	-0.157	0.611	0.295	0.013
N_1-Si_6	2.034	1.321	0.114	-0.178	-0.168	0.937	0.591	0.064
Si_6-N_7	1.318	2.024	0.115	-0.181	-0.171	0.957	0.605	0.060
Si-F	1.291	1.800	0.110	-0.196	-0.191	0.138	0.990	0.023
cent-F4-								
$N_1=C_2$	1.600	0.836	0.364	-0.770	-0.724	0.585	-0.909	0.064
C_2-Si_3	2.258	1.374	0.107	-0.137	-0.133	0.555	0.285	0.033
N_1-Si_6	2.017	1.314	0.118	-0.186	-0.176	0.977	0.615	0.055
Si_6-N_7	1.322	2.018	0.111	-0.174	-0.164	0.948	0.610	0.062
$C_{8,10}$-F	0.915	1.690	0.238	-0.462	-0.407	0.667	-0.202	0.136
Si-H	1.363	1.421	0.111	-0.153	-0.153	0.584	0.278	0.000
F8-								
$N_1=C_2$	1.562	0.815	0.396	-0.956	-0.800	0.728	-0.103	0.195
C_2-Si_3	2.258	1.370	0.104	-0.139	-0.133	0.593	0.320	0.048
N_1-Si_6	2.002	1.314	0.115	-0.183	-0.174	0.994	0.637	0.055
Si_6-N_7	1.318	2.011	0.113	-0.179	-0.168	0.969	0.622	0.061
$C_{2,4}$-F	0.912	1.692	0.238	-0.465	-0.398	0.668	-0.194	0.169
$C_{8,10}$-F	0.909	1.688	0.241	-0.471	-0.414	0.685	-0.200	0.138
Si-H	1.357	1.409	0.115	-0.161	-0.160	0.602	0.281	0.007
F12-								
$N_1=C_2$	1.561	0.817	0.396	-0.954	-0.792	0.713	-0.103	0.205
C_2-Si_3	2.237	1.355	0.113	-0.159	-0.156	0.635	0.321	0.016
N_1-Si_6	2.007	1.316	0.114	-0.182	-0.172	0.984	0.631	0.059
Si_6-N_7	1.316	2.007	0.114	-0.181	-0.171	0.982	0.631	0.060
$C_{2,4}$-F	0.899	1.685	0.244	-0.483	-0.421	0.714	-0.190	0.149
$C_{8,10}$-F	0.904	1.687	0.242	-0.477	-0.419	0.698	-0.198	0.140

Si-F	1.280	1.780	0.115	-0.210	-0.206	1.484	1.068	0.017

Table S27. Dependency of the NICS values in [ppm] on the distance r_{Bq} in [Å] of the ghost atom *Bq* in: ***(I)*** OITSSHC-33, ***(II)*** OSITSSHC-33 and ***(III)*** OGITSSHC-33. (a) the *Bq* ghost atoms positioned above the silicon spiro center Si_9/Ge_9, (b) the *Bq* atoms array is set perpendicular to the center of the spiro-ring-plane, (see Figures 11 and S18). B3LYP/aug-cc-pVDZ method was used.

	(I)		***(II)***		***(III)***	
r_{Bq}	NICS[a]	NICS[b]	NICS[a]	NICS[b]	NICS[a]	NICS[b]
0.0		5.69		0.75		1.91
0.5	-19.00	4.03	-26.30	032	-67.30	1.41
1.0	-7.63	1.80	-14.60	-0.20	-15.73	0.58
1.5	3.07	0.90	-5.32	-0.32	-5.77	0.11
2.0	3.60	0.57	0.27	-0.25	-0.52	-0.05
2.5	-0.87	0.33	0.62	-0.15	0.57	-0.08
3.0	-1.20	0.15	-0.77	-0.09	-0.34	-0.07
3.5	-0.38	0.05	-1.05	-0.05	-0.83	-0.05
4.0	-0.12	-0.01	-0.56	-0.02	-0.50	-0.03
4.5	-0.07	-0.04	-0.21	-0.01	-0.15	-0.02
5.0	-0.07	-0.05	-0.06	-0.01	0.00	-0-02

(a) the *Bq* ghost-0.06 atoms positioned above the silicon spiro center Si_6.

(b) the *Bq* atoms are set perpendicular to the center of the spiro-ring-plane.

Table S28. Dependency of the $NICS_{zz}$ values in [ppm] on the distance r_{Bq} in [Å] of the ghost atom *Bq* in: ***(I)*** OITSSHC-33, ***(II)*** OSITSSHC-33 and ***(III)*** OGITSSHC-33. (a) the *Bq* ghost atoms positioned above the silicon spiro center Si_9/Ge_9, (b) the *Bq* atoms array is set perpendicular to the center of the spiro-ring-plane, (see Figures 11 and S18). B3LYP/aug-cc-pVDZ method was used.

	(I)		***(II)***		***(III)***	

r_{Bq}	$NICS_{zz}$ a	$NICS_{zz}$ b	$NICS_{zz}^{a}$	$NICS_{zz}$ b	$NICS_{zz}^{a}$	$NICS_{zz}^{b}$
0.0		-1.05		-1.26		-1.02
0.5	2.98	-1.62	-48.93	-1.39	-48.41	-0.90
1.0	-1.85	-0.78	-18.22	-0.90	-6.16	-0.16
1.5	4.84	1.18	-9.10	0.16	2.46	0.93
2.0	4.46	2.16	-3.55	0.89	6.65	1.68
2.5	1.34	2.24	-0.99	1.15	6.36	1.93
3.0	1.85	2.02	-0.42	1.14	4.29	1.86
3.5	2.17	1.73	-0.94	1.03	2.76	1.66
4.0	1.73	1.43	-1.35	0.88	2.09	1.43
4.5	1.29	1.16	-1.29	0.74	1.65	1.20
5.0	1.00	0.94	-1.08	0.60	1.29	1.17

(a) the *Bq* ghost atoms positioned above the silicon spiro center Si_6.

(b) the *Bq* atoms are set perpendicular to the center of the spiro-ring-plane.

Figures S1-S18:

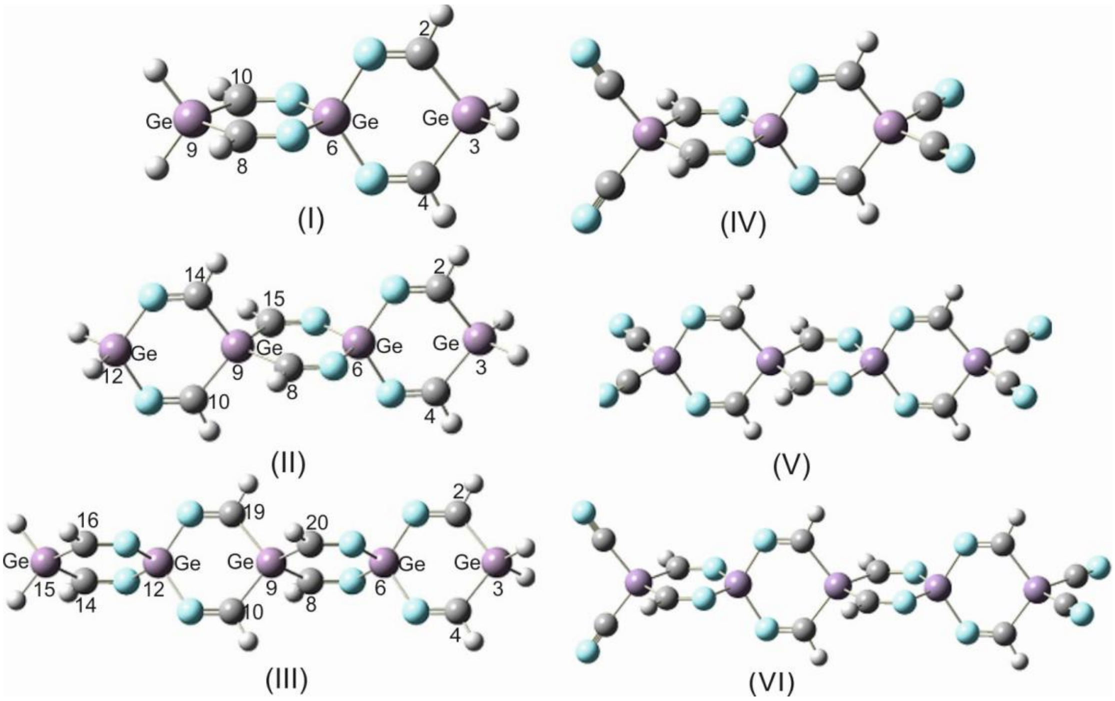

Figure S1: (I) 2,4,8,10-tertraimino-3,9-digerma-6-germaspiro[5.5]undecane (TIGSNU-19).

(II) 2,4,8,10,14,15-hexaimino-3,12-digerma-6,9-digermaspiro[5.2.59.26]hexadecane (HIDGSNCH-26).

(III) 2,4,8,10,14,16,19,20-octaimino-3,15-digerma-6,9,12-trigermaspiro[5.2.2.512.29.26]henicosane (OITGSHC-33).
(IV)-(VI) corresponding tetracyano derivatives.

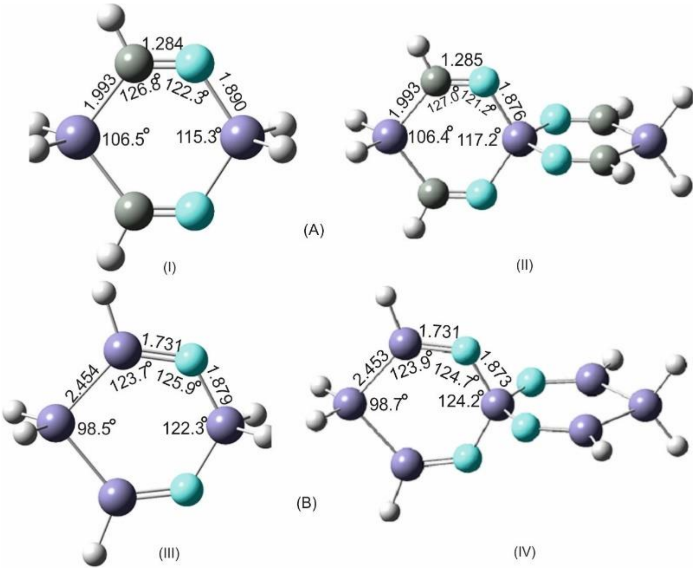

Figure S2: (I) Structural parameters of the building blocks of
(A) Germa-spiro cyclic imines:
(I) 1,4-digermacyclohexa-2,6-diimine (DGCHDI-12,
(II) TIGSNU-19
(B) Germa-spiro cyclic germaimines:
(III) 1,4-digermacyclohexa-2,6-digermaimine (DGCHDGI-12,
(IV) TGIGSNU-19.
B2PLYP/aug-cc-pVDZ was used.

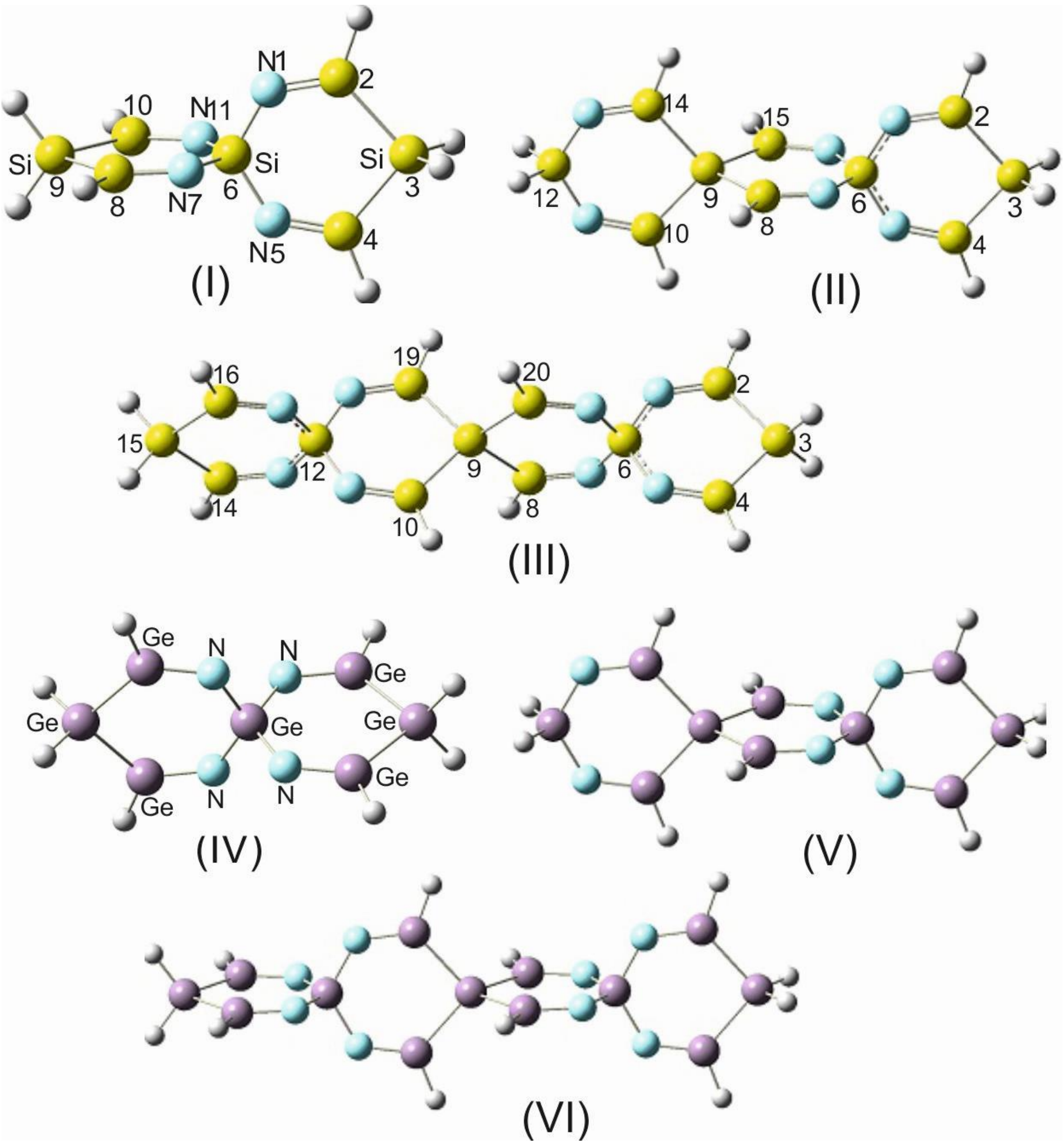

Figure S3: (I) 2,4,8,10-tetrasilaimino-3,9-disila-6-silaspiro[5,5]undecane (TSISSNU-19).
(II) 2,4,8,10,14,15-hexasilaimino2-disila-6,9-disila[5.2.59.26]hexadecane (HSISSNCH-26).
(III) 2,4,8,10,14,16,19,20-octasilaimino-3.15-disila-6,9,12-trisilaspiro[5.2.2.512.29.26]henicosane (OSITSSHC-33).
(IV), (V) and (VI) are the corresponding germaimino analogs: TGIGSNU, (V) HGIGSNCH-26 and (VI) OGIGSHC-33).

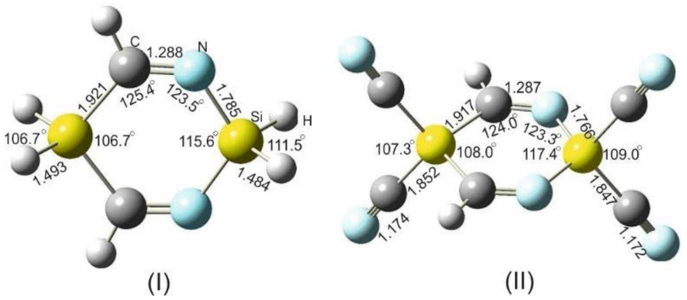

Figure S4: (I) Structural parameters of the „primordial“ building block of Sila-spirocyclic imines (I): 1,4-disilacyclohexa-3,5-diimine (DSCHDI). and ist tetracyano derivative (II) (TCDSCHDI). B2PLYP/aug-cc-pVDZ was used.

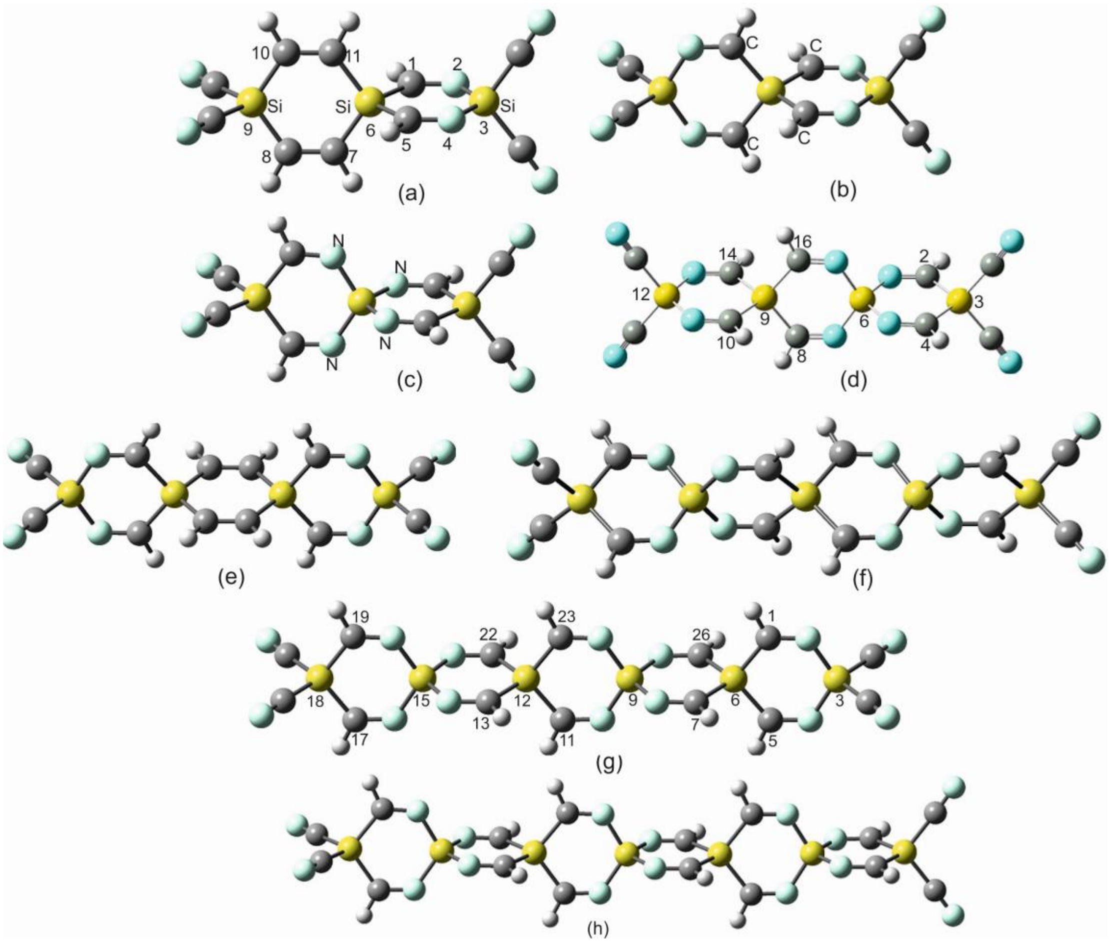

Figure S5: (a) 3,3,,9,9-tetracyano-1,5-diimino-3,9-disila-6silaspiro[5.5]undecane (TCDISSCU-25).

(b) 3,3,9,9-tetracyano-1,5,7,11-tetraimino3,9-disila-6-silaspiro[5.5]undecane (TCTISSCU-23).

(c) 3,3,9,9-tetracyano-2,4,6,10-tertraimino-3,9-disila-6-silaspiro[5.5]undecane (TCTISSNU-23).

(d) 3,3,12,12-tetracyano-2,4,8,10,14,15-hexaimino-3,12-disila-6,9-disilaspiro[5.2.59.26]hexadecane (TCHIDSSNCH-30)

(e) 3,3,12,12-tetracyano-1,5,10,14-tetraimino-3,12-disila-6,9-disilaspiro[5.2.59.26]hexadecane (TCTIDSSCCH-32).

(f) 3,3,15,15-tetrayano-2,4,8,10,14,16,19,20-octaimino-3,15-disila-6,9,12-trisilaspiro[5.2.2.512.29.26]henicosane (TCOITSSHC-37).

(g) 3,3,18,18-tetracyano-1,5,7,11,13,17,19,22,23,26-decaimino-3,18-disila-6,9,12,15-tetrasilaspiro[5.2.2.2.515.212.29.26]hexacosane (TCDITSSHC-44).

(h) 3,3,21,21-tetracyano-2,4,8,10,14,16,20,22,25,26,29,30-dodecaimino-3,21-disila-6,9,12,15,18-pentasilaspiro[5.2.2.2.2.518.215.212.29.26]hentriacontane (TCDIPSSHT-51).

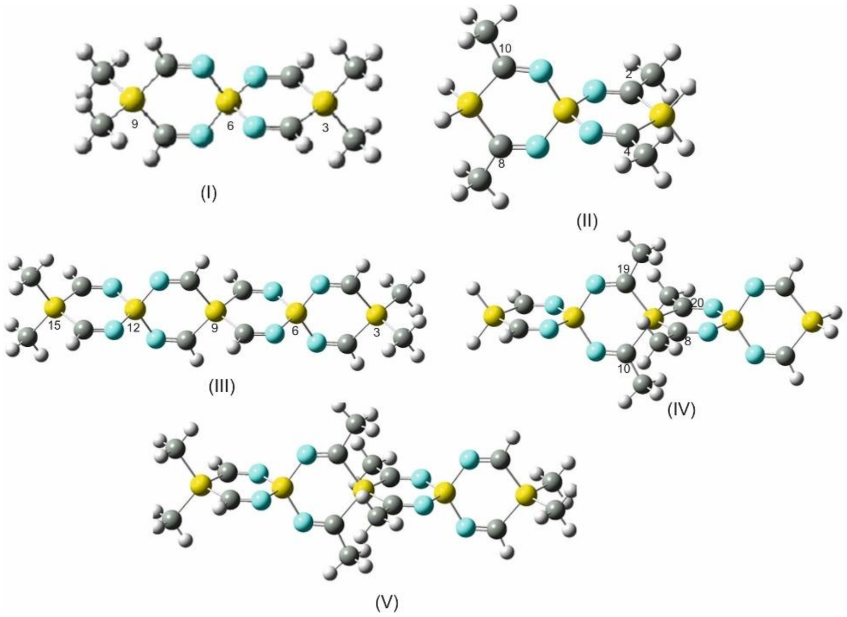

Figure S6: Some methyl derivatives of TISSNU-19 and OITSSHC-33:
(I) 3,3,9,9-tetramethyl-TISSNU-31. (II) 2,4,8,10-tetramethyl-TISSNU-31.
(III) 3,3,15,15-tetramethyl-OITSSHC-45. (IV) 8,10.19,20-tetramethyl-OITSSHC-45.
(V) 3,3,8,10,15,15,19,20-octamethyl-OITSSHC-57.
B3LYP/aug-cc-pVDZ was employed.

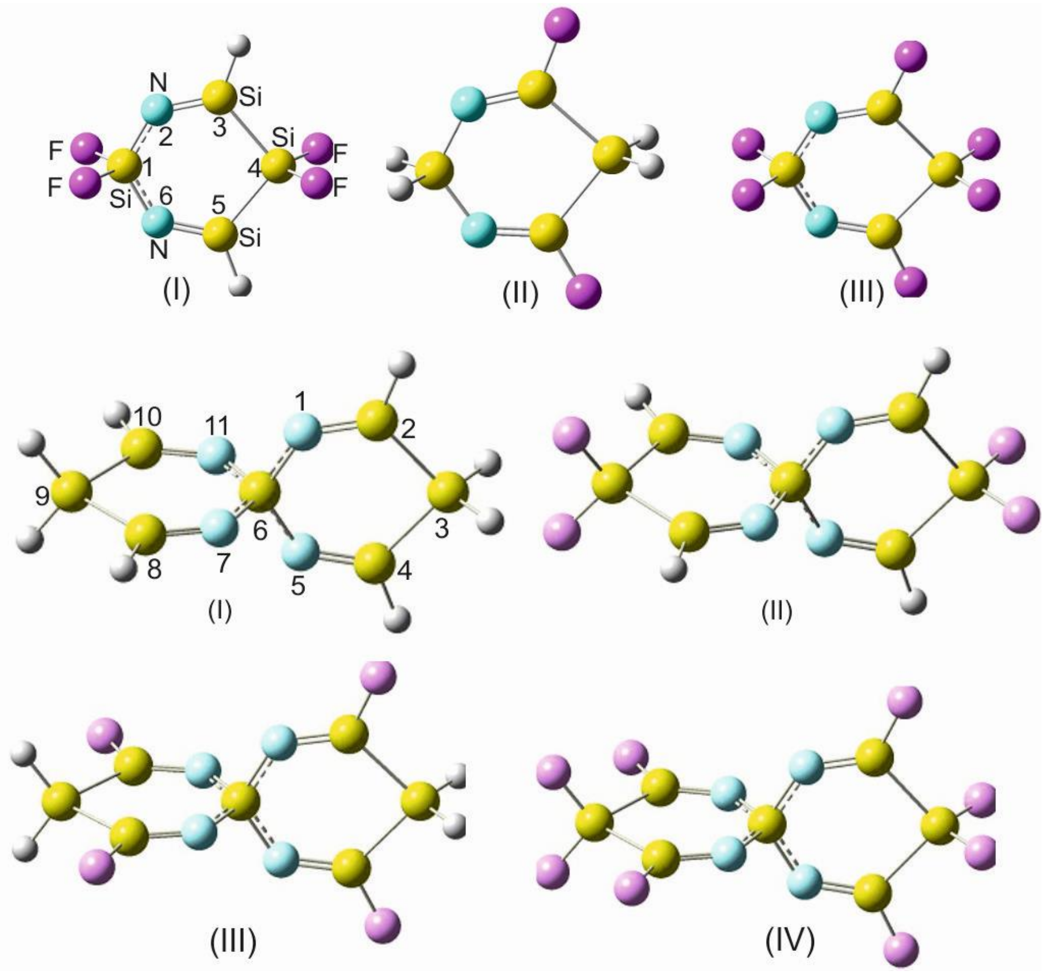

Figure S7: (I) 1,1,4,4-tetrafluoro-1,4-disila3,5-disilaimino-cyclohexane (trme-F4-DSDSICH-12).

(II) 3,5-difluoro-DSDSICH-12 (cent-F2-DSDSICH-12.

(III) perfluoro-DSDSICH-12 (F6-DSDSICH-12).

(IV) 2,4,8,10-tetrasilaimino-3,9-disila-6-silaspiro[5,5]undecane (TSISSNU-19).

(V) terminal-3,3,9,9-tetrafluoro-TSISSNU-19 (term-F4-TSISSNU-19).

(IVI) central-2,4,8,10-tetrafluoro-TSISSNU-19 (cent- F4-TSISSNU-19).

(IV) perfluro-TSISSNU-19 (F8-TSISSNU-19)

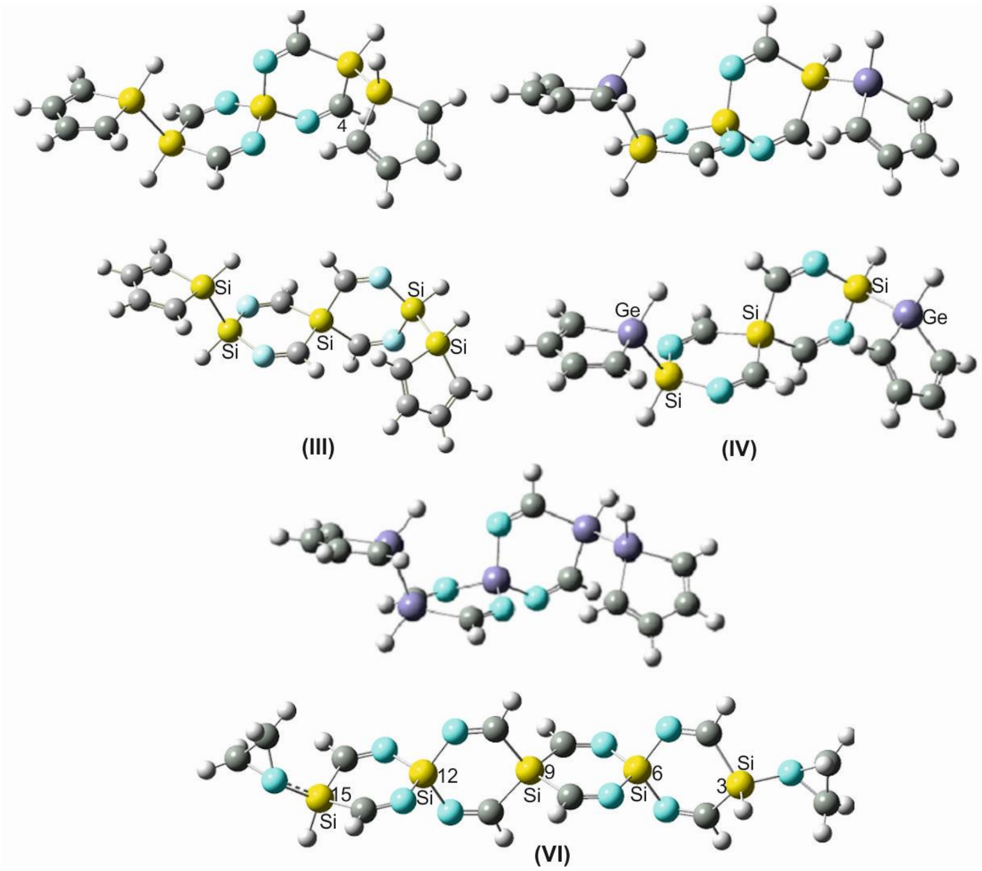

Figure S8: Diverse terminally substituted CIMs:
(I) 3,9-disilole-TISSNU-19. (II) 3,9-digermole-TISSNU-19.
(III) 3,9-disilole-TISSCU-19. (IV) 3,9-digermole-TISSCU-19.
(V) 3,9-digermole-TIGSNU-19. (VI) 3,15-diaziridine-OITSSHC-33.

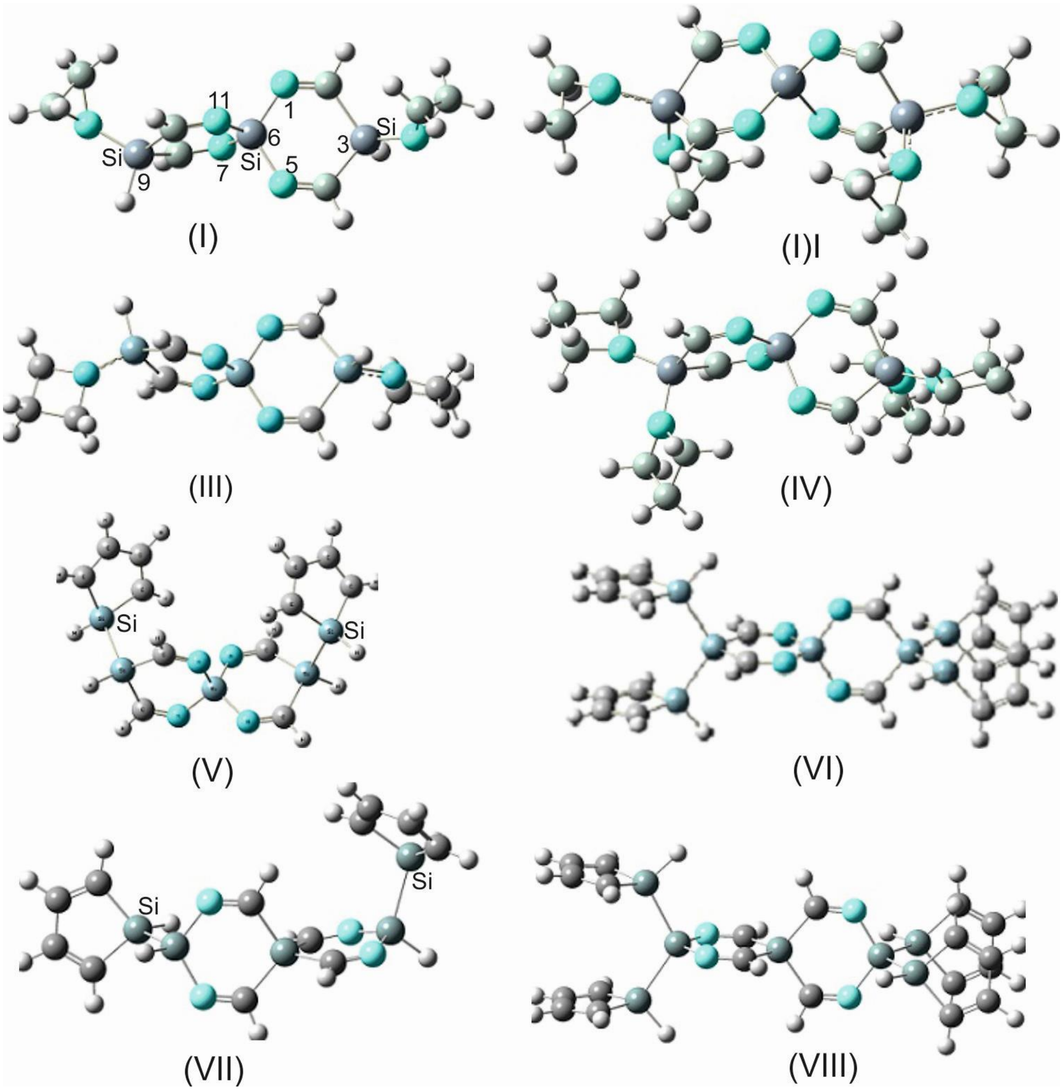

Figure S9: Different 3,9-di- (left) and tetra-substiuted (right) derivatives of TISSNU-19:

(I) 3,9-diaziridine (DIAZITISSNU-31), (II) 3,3',9,9'-tetraaziridine (TAZITISSNU-43).

(III) 3,9-diazetidine (DAZETISSNU-37). (IV) 3,3',9,9'-tetraazetidine (TAZETISSNU-55).

(V) 3,9-disilole (DSILTISSNU-37). (VI) 3,3',9,9'-tetrasilole (TSILTISSNU-55).

(VII) 3,9-disilole (DSILTISSCU-37), (VIII) 3,3',9,9'-tetrasilole (TSILTISSCU-55).

(VII) and (VIII) are derivatives of TISSCU-19).

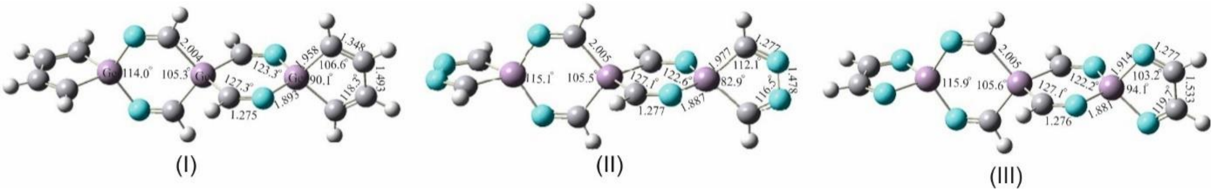

Figure S10: Structural parameters of some germinally incorporated gelole and diminogelole in the TIGSNU-19
scaffold (C-Ge-C spiro center):
(I) 7,9,17,18-tetraimino-5,8,11-trigermaspiro[4.2.2.411.28.25]nonadeca-1,3,12,14-tetraene (TITGSNDTE-31).
(II) 1,4,7,9,12,15,17,18-octaimino-5,8,11-trigermaspiro[4.2.2.411.28.25]nonadecane (OITGSND-D-27).
(III) 2,3,7,9,13,14,17,18-octaimino-5,8,11-trigermaspiro[4.2.2.411.28.25]nonadecane (OITSSND-V-27).
For these abbreviations see text. B3LYP/aug-cc-pVDZ was used.

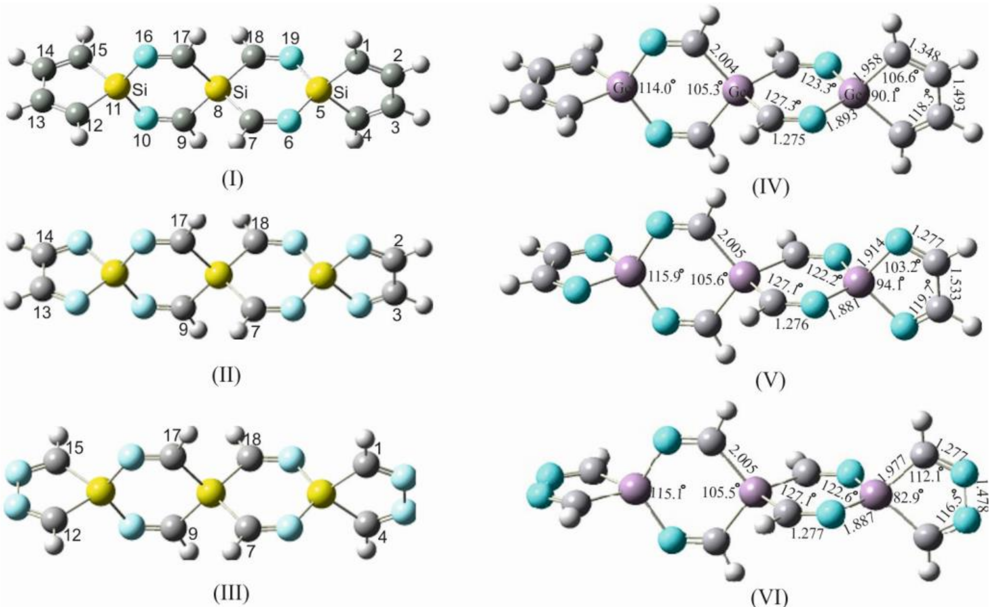

Figure S11: Some sila- and germa-spiro cyclicimines derivatives with
terminally incorporated silole or gelole and their diiminoderivatives.
(I) 7,9,17,18-tetraimino-5,8,11-trisilaspiro[4.2.2.411.28.25]nonadeca-1,3,12,14-tetraene (TITSSNDTE-31).
(II) 2,3,7,9,13,14,17,18-octaimino-5,8,11-trisilaspiro[4.2.2.411.28.25]nonadecane (OITSSND-V-27).
(III) 1,4,7,9,12,15,17,18-octaimino-5,8,11-trisilaspiro[4.2.2.411.28.25]nonadecane (OITSSND-D-27).
(IV) 7,9,17,18-tetraimino-5,8,11-trigermaspiro[4.2.2.411.28.25]nonadeca-1,3,12,14-tetraene (TITGSNDTE-31).

(V) 2,3,7,9,13,14,17,18-octaimino-5,8,11-trigermaspiro[4.2.2.411.28.25]nonadecane (OITGSND-V-27).
(VI) 1,4,7,9,12,15,17,18-octaimino-5,8,11-trigermaspiro[4.2.2.411.28.25]nonadecane (OITSSND-D-27).

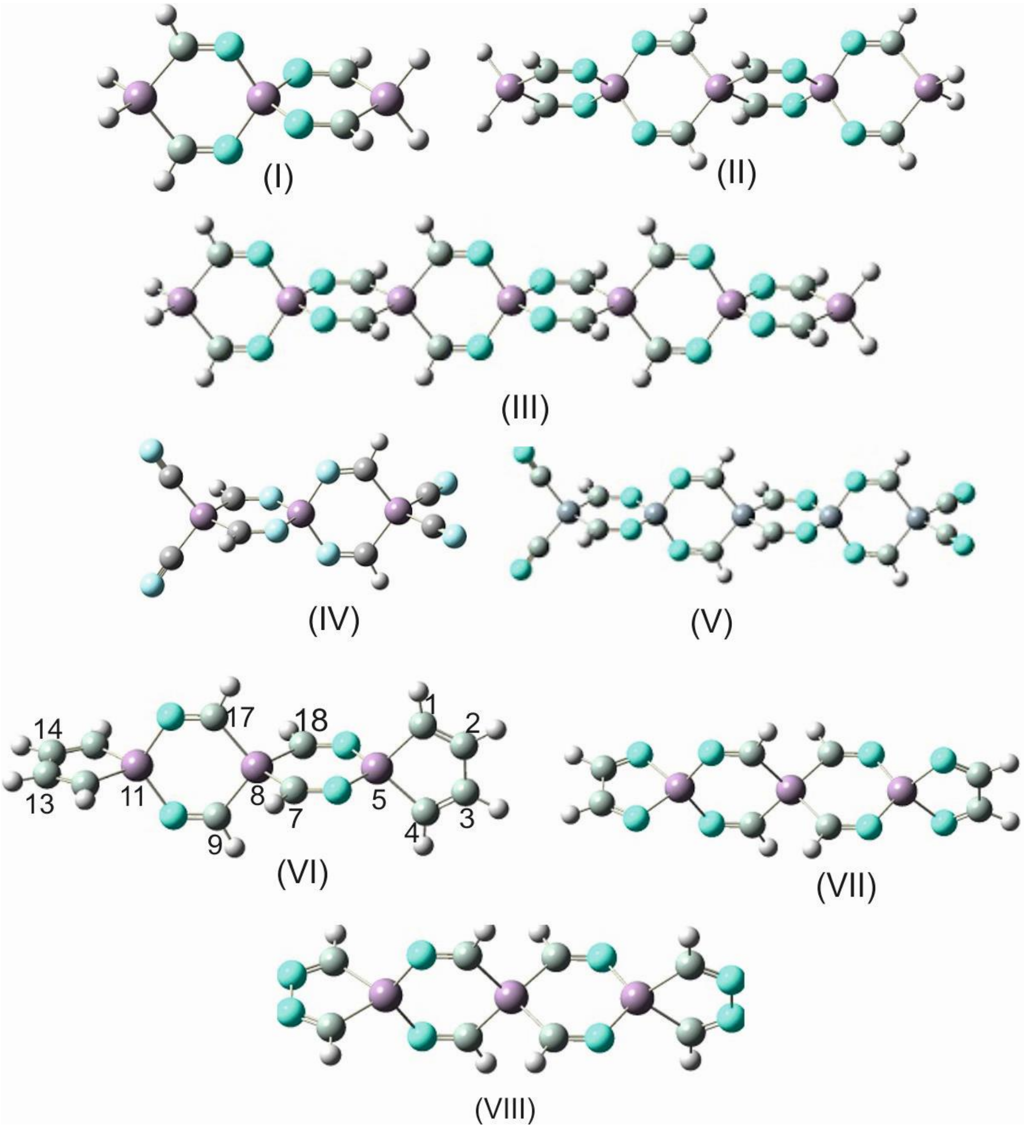

Figure S12: Some germaspiro cyclicimine derivatives:
(I) 1,5,7,11-tertraimino-3,9-digerma-6-germaspiro[5.5]undecane (TIGSNU-19).
(II) 1,5,7,11,13,17,18,21-octaimino-3,15-digerma-6,9,12-trigermaspiro[5.2.2.512.29.26]henicosane (OITGSHC-33).
(III) 1,5,7,11,13, 17,19,23,24,27,28,31-dodecaimino-3,21-digerma-6,9,12,15,18-pentagermaspiro
[5.2.2.2.2.518.215.212.29.26]hentriacontane (DDIPGSHT-47).
(IV) TCTIGSNU-23. (V) TCOITGSHC-37.
(VI) 7,9,17,18-tetraimino-5,8,11-trigermaspiro[4.2.2.411.28.26]nonadeca-1.3.12,14-tetraene (TITGSNDTE-31).

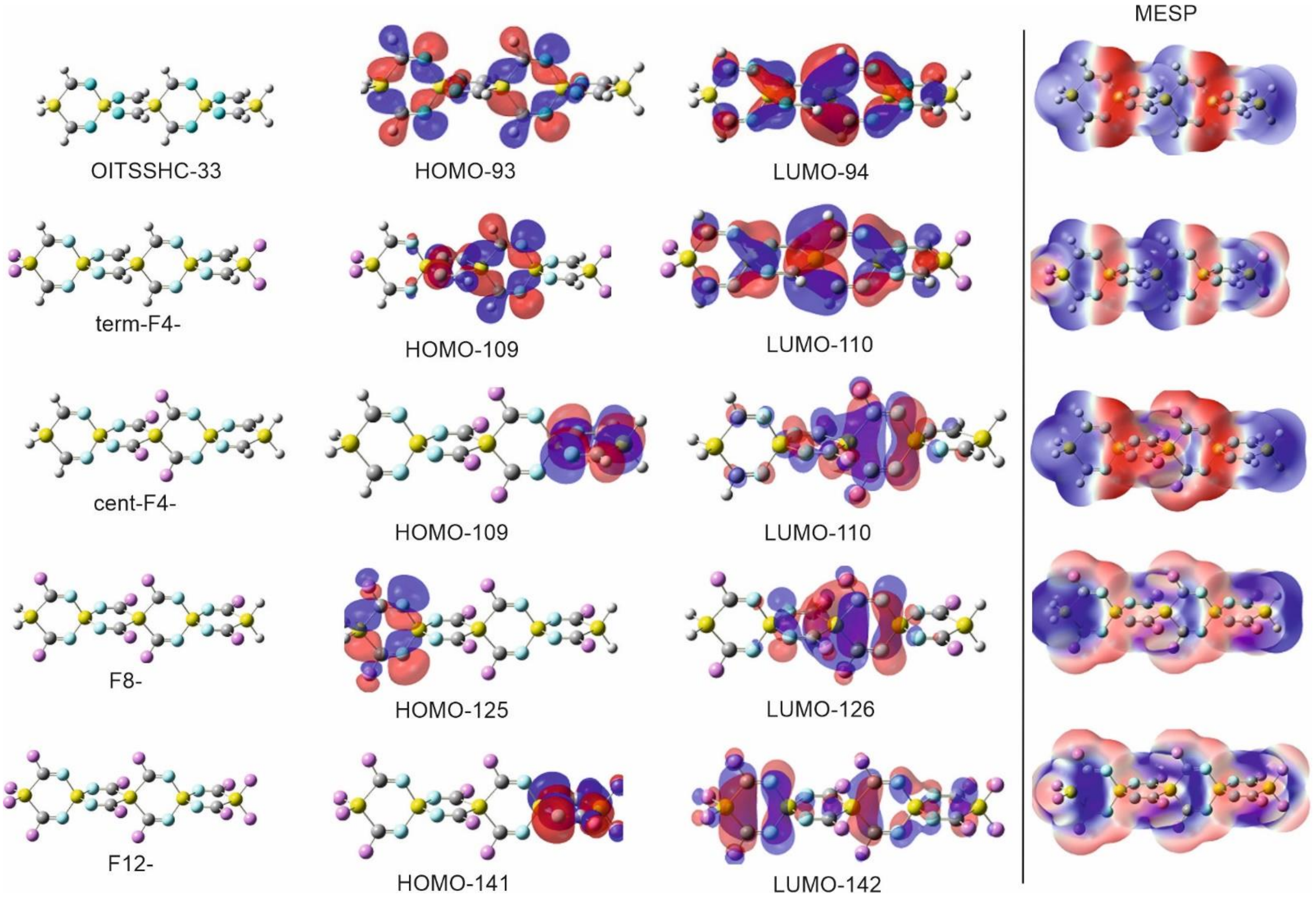

(VII) 2,3,7,9,13,14,17,18-octaimino-5,8,11-trigermaspiro[4.2.2.411.28.25]nonadecane (OITGSND-V-27).

(VIII) 1,4,7,9,12,15,17,18-octaimino-5-8-11-trigeermaspiro[4.2.2.411.28.25]nonadecane (OITGSND-D-27).

Figure S13: Comparison between the HOMO and LUMO frontier molecular orbitals FMOs of octaimino-trisilaspiro-henicosane (OITSSHC-33) and its fluorine derivatives: 3,315.15-tetrafluor- (terminal tetrafluoro =term-F4-), 8,10,19,20-tetrafluoro- (central tetrafluoro= cent-F4-), 2,4,8,10,14,16,19,20-ctafluoro- (F8-), and 2,3,3,4,8,10,14,15,15,16,19,20-dodecafluoro- (F12-). Right column represents the corresponding molecular electrostatic potentials (MESPs)

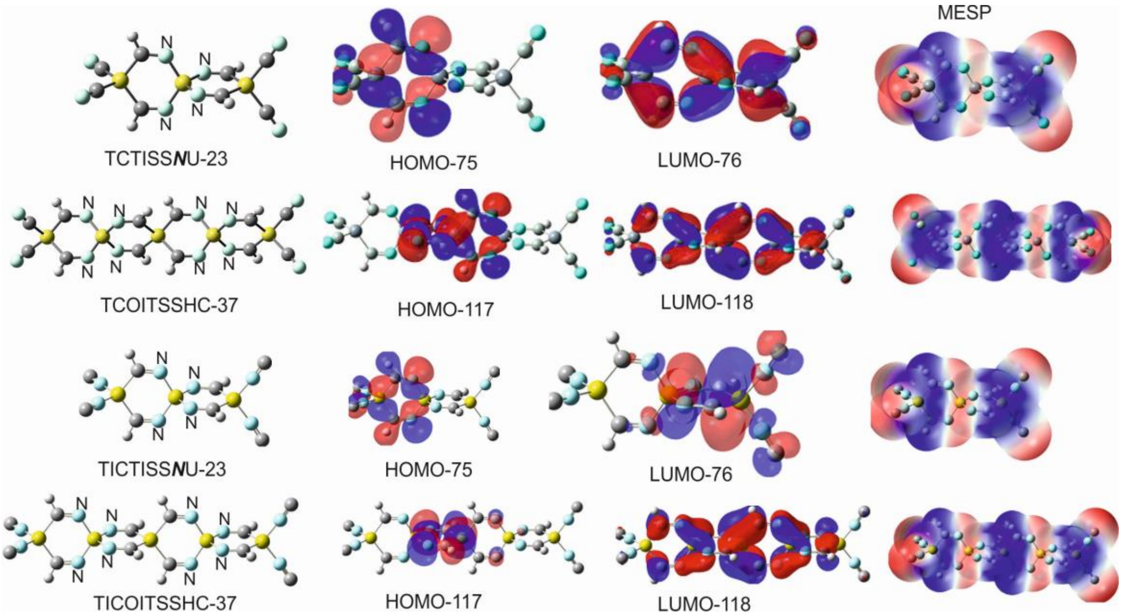

Figure S14: Comparison between frontier molecular orbitals (FMOs) and molecular electrostaic potentials (MESP) of
the terminally substituted tetracyano silaspirocyclic imines TCTISSNU-23, TCOITSSHC-37 and the corresponding
tetraisocyano analogues TICTISSNU-23 and TICOITSSHC-37 with N-Si-N spiro center and alternating N-Si-N.and
C-Si-C spiro centers.

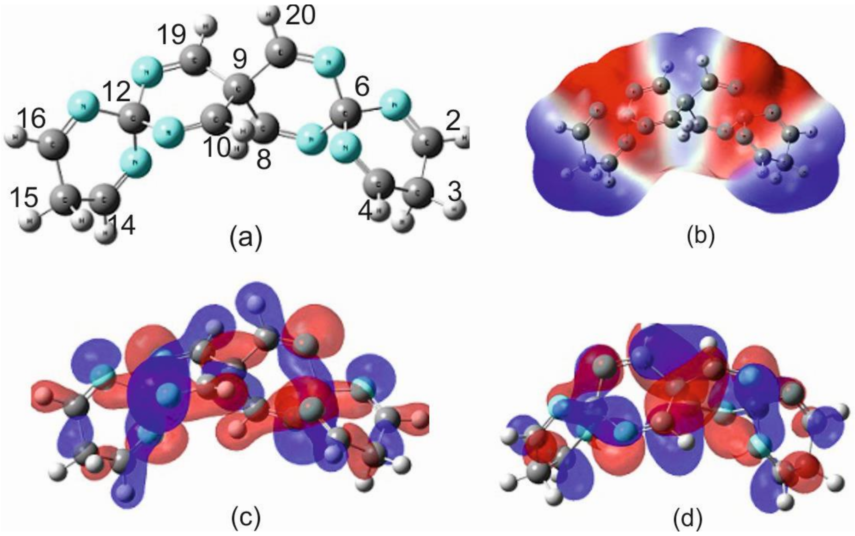

Figure S15: Carbospiro cyclic imine with alternating N-C-N and C-C-C spiro centers: (a) 2,4,8,10,14,16,19,20-octaimine-6,9,12-tricarbospiro[5.2.2.512.29.26]henicosane. (OITCSHC-33). (b) MESP and the molecular frontier orbitals: (c) HOMO, (d) LUMO

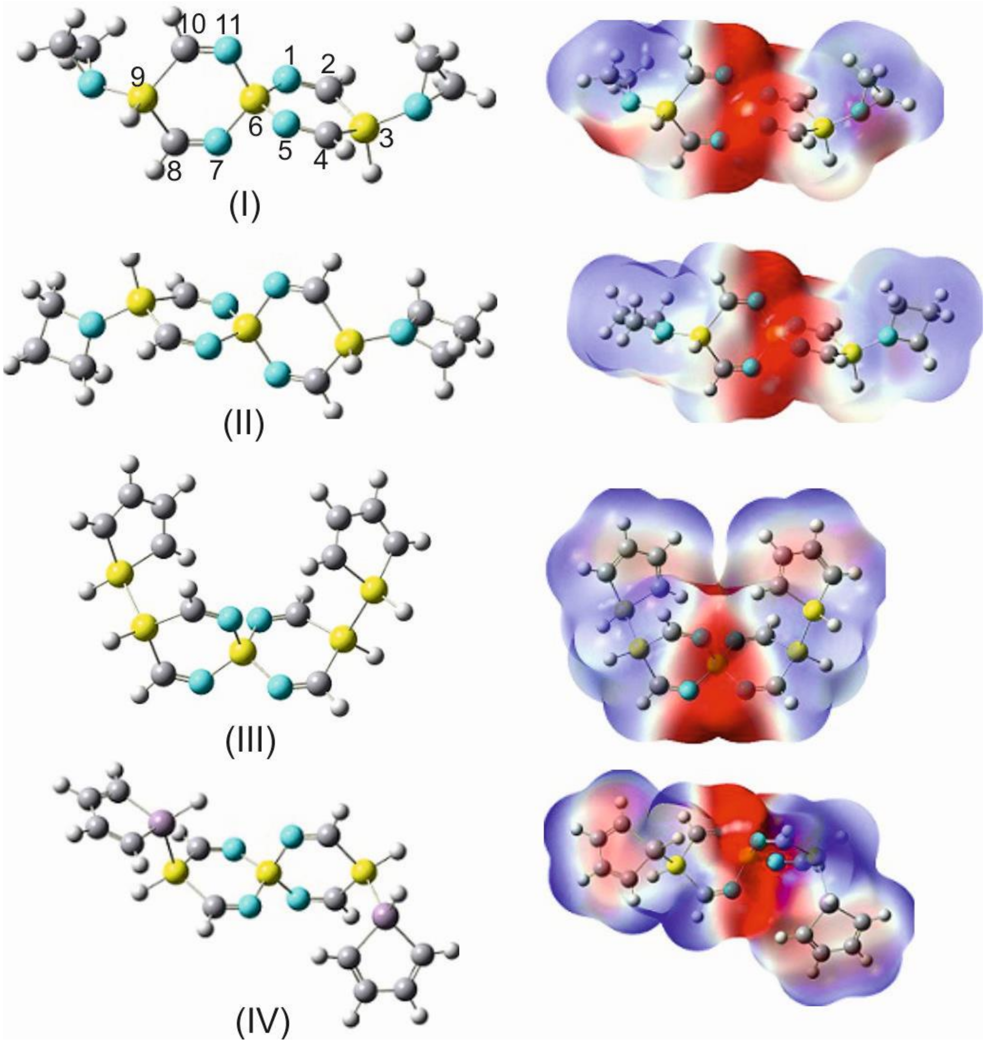

Figure S16: Some terminally substituted derivatives of TISSNU-19 (N-Si-N spiro-center) with their MESPs mapping:
(I) 3,9-diaziridine-, (II) 3,9-diazetidine-, (III) 3,9-disilole-, (IV) 3,9-digermole-.

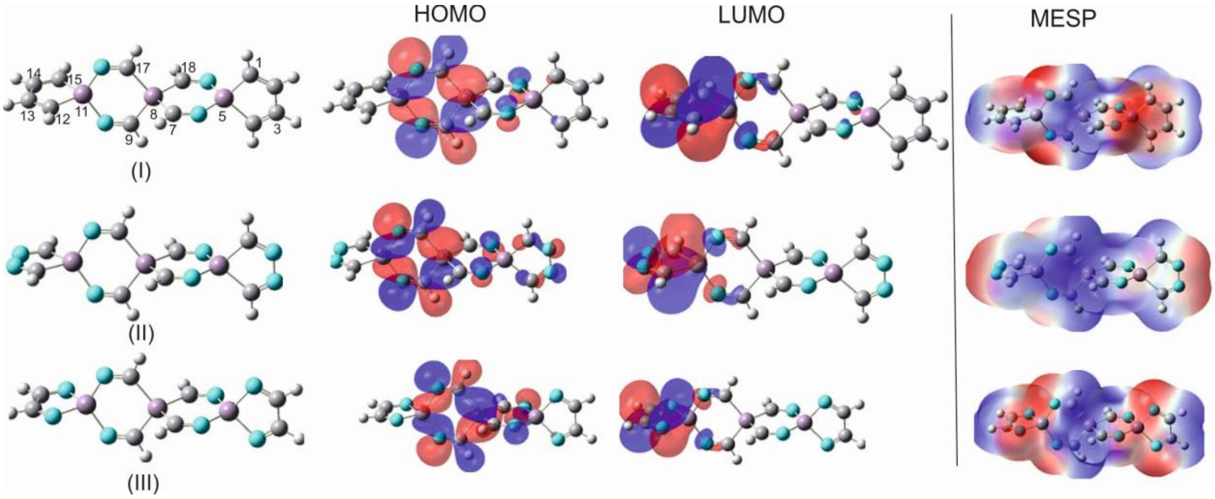

Figure S17: Comparison of the HOMO and LUMO frontier orbitals and the molecular electrostatic potenials (MESPs)
of terminally incorporated gelole and diminogelole in the TIGSNU-19 scaffold (C-Ge-C spiro center):

(I) 7,9,17,18-tetraimino-5,8,11-trigermaspiro[4.2.2.411.28.25]nonadeca-1,3,12,14-tetraene (TITGSNDTE-31).

(II) 1,4,7,9,12,15,17,18-octaimino-5,8,11-trigermaspiro[4.2.2.411.28.25]nonadecane (OITGSND-D-27).

(III) 2,3,7,9,13,14,17,18-octaimino-5,8,11-trigermaspiro[4.2.2.411.28.25]nonadecane (OITSSND-V-27). For these abbreviations see text.

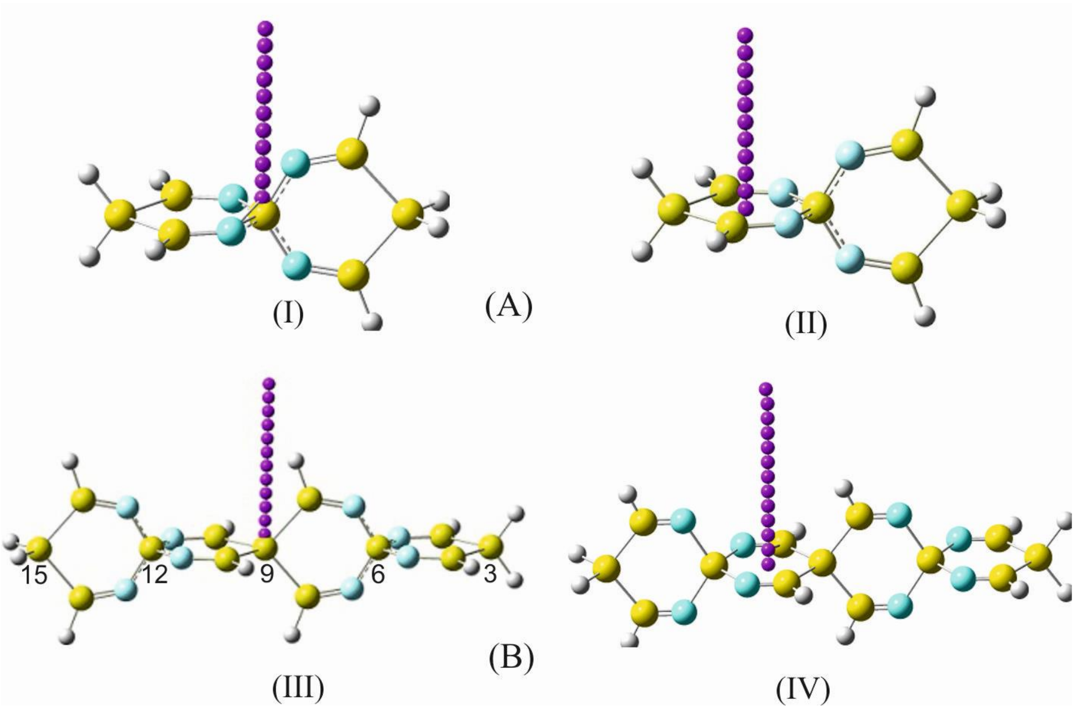

Figure S18: (A) Position of the Bq atoms in TSISSNU-19:
(I) above the spiro center Si6. (II) perpendicular to the center of a ring plane.
(B) Position of the Bq atoms in OSITSSHC-33:
(III) above the central spiro silicn Si9- (IV)perpendicular to the center of a ring plane.
